# Mitochondrial metabolism and epigenetic crosstalk drive SASP

**DOI:** 10.1038/s41586-026-10791-2

**Published:** 2026-07-29

**Authors:** Hélène Martini, Jodie Birch, Francisco D. M. Marques, Stella Victorelli, Anthony B. Lagnado, Nicholas Pirius, Ana Catarina Franco, Gung Lee, Yeaeun Han, Jennifer L. Rowsey, Wazim Mohammed Ismail, Amelia Mazzone, Tianna M. Espe, Taro Hitosugi, Ya Li, Alexander M. Washington, Aaron Havas, Rabi Murad, Xue Lei, Rebecca A. Porritt, Oliver D. K. Maddocks, Jair Machado Espindola-Netto, Dominik Saul, Sundeep Khosla, Diana Jurk, Enis Kostallari, Alexandre Gaspar-Maia, Peter D. Adams, João F. Passos

**Affiliations:** 1 Department of Physiology and Biomedical Engineering, Mayo Clinic, Rochester, MN USA; 2 Robert and Arlene Kogod Center on Aging, Mayo Clinic, Rochester, MN USA; 3 MRC Laboratory of Medical Sciences (LMS), London, UK; 4 Institute of Clinical Sciences (ICS), Faculty of Medicine, Imperial College London, London, UK; 5 Department of Biochemistry, Albert Einstein College of Medicine, Bronx, NY USA; 6 Division of Experimental Pathology, Department of Lab Medicine and Pathology, Mayo Clinic, Rochester, MN USA; 7 Epigenomics Program, Center for Individualized Medicine, Mayo Clinic, Rochester, MN USA; 8 Department of Oncology, Division of Oncology Research, Mayo Clinic, Rochester, MN USA; 9 Molecular Pharmacology and Experimental Therapeutics Graduate Program, Mayo Clinic Graduate School of Biomedical Sciences, Rochester, MN USA; 10 Division of Gastroenterology and Hepatology, Mayo Clinic, Rochester, MN USA; 11 Mayo Clinic Graduate School of Biomedical Sciences, Mayo Clinic, Rochester, MN USA; 12 Sanford Burnham Prebys Medical Discovery Institute, La Jolla, CA USA; 13 School of Cancer Sciences, Wolfson Wohl Cancer Research Centre, University of Glasgow, Glasgow, UK; 14 Department of Radiation Oncology, Mayo Clinic, Rochester, MN USA; 15 Robert Bosch Center for Tumor Diseases, Stuttgart, Germany; 16 Eberhard Karls University Tuebingen, Tuebingen, Germany; 17 Department of Biochemistry and Molecular Biology, Mayo Clinic, Rochester, MN USA

**Keywords:** Senescence, Epigenetics

## Abstract

Senescent cells promote tissue dysfunction in part through the senescence-associated secretory phenotype (SASP)^1^. Cytosolic mitochondrial nucleic acids activate innate immune signalling to initiate this inflammatory programme^2,3^. Here we show that mitochondrial metabolism provides a second layer of control that enables execution of the inflammatory programme. In senescent cells, the mitochondrial pyruvate–citrate–acetyl-CoA axis is upregulated, increasing the availability of acetyl-CoA to support histone acetylation at SASP genes. Whereas mitochondrial DNA-driven signalling activates inflammatory transcription factors, acetyl-CoA availability is required for robust transcription of SASP genes. Accordingly, enhancing acetyl-CoA levels promotes SASP gene expression, whereas inhibition of SLC25A1, the mitochondrial citrate exporter, reduces histone acetylation at SASP loci, limiting activity of this programme. In vivo, inhibition of SLC25A1 reduces chromatin accessibility at SASP loci, dampens inflammation and improves healthspan in aged mice. Together, these findings identify a mitochondrial metabolic checkpoint that enables the epigenetic execution of innate immune signalling, revealing a mechanism that selectively controls the inflammatory output of senescent cells.

## Abstract (web summary)

In senescent cells, mitochondria-derived acetyl-CoA promotes histone acetylation and increases chromatin accessibility at inflammatory gene loci. Inhibition of SLC25A1 attenuates these effects, underscoring the therapeutic potential of targeting mitochondrial metabolism and its epigenetic crosstalk to delay age-related functional decline.

## Main

Cellular senescence is a key response to various cellular stresses, characterized by a permanent cell cycle arrest and the secretion of a pro-inflammatory cocktail of factors known as SASP^1^. Senescence serves important roles in embryonic development^4,5^, tumour suppression^6^ and tissue repair^7,8^. However, persistent SASP activation can lead to chronic inflammation, contributing to tissue dysfunction, ageing and a broad spectrum of age-related diseases, including cancer^1^. This dual nature makes SASP a focal point for understanding both protective and detrimental effects of senescence.

Mitochondria, which are central to cellular metabolism, undergo substantial changes during senescence^9,10^. These include impaired bioenergetic function, disrupted mitochondrial dynamics and metabolic reprogramming, particularly involving the tricarboxylic acid (TCA) cycle^11^. Increased levels of TCA cycle intermediates, such as acetyl-CoA, fumarate and succinate, accumulate in senescent cells^11^.

Beyond metabolic remodelling, mitochondria in senescent cells generate innate immune signals. In senescence and ageing, mitochondrial dysfunction promotes cytosolic release of mitochondrial DNA (mtDNA), which activates cGAS–STING signalling and drives inflammatory responses and SASP^3,12,13^. How these inflammatory signals integrate with mitochondrial metabolic cues to regulate SASP gene expression remains unclear.

Here we show that mitochondrial metabolism is a key determinant of SASP expression through regulation of histone acetylation at inflammatory gene loci. In senescent cells, pathways that control cytosolic acetyl-CoA production, including mitochondrial citrate export and its conversion by ATP-citrate lyase, are upregulated. This metabolic rewiring sustains chromatin accessibility at SASP regulatory elements. Disrupting this axis suppresses SASP expression despite persistent cytosolic mtDNA signalling. Together, these findings indicate that mitochondrial innate immune signalling requires metabolic–epigenetic regulation to drive SASP gene expression. Pharmacological inhibition of this pathway in aged mice selectively suppresses SASP-associated inflammation without reversing cellular senescence, reducing tissue inflammation and improving healthspan.

## Mitochondria promote acetylation at SASP loci

Acetyl-CoA is the obligate donor for protein acetylation, including histone acetylation^14,15^, a modification associated with chromatin relaxation and increased accessibility to transcriptional machinery. Although acetyl-CoA can be generated through several metabolic pathways, mitochondrial metabolism represents a major hub of acetyl group production. However, the extent to which mitochondrial metabolism contributes to histone acetylation during cellular senescence is not known.

**Fig. 1:**
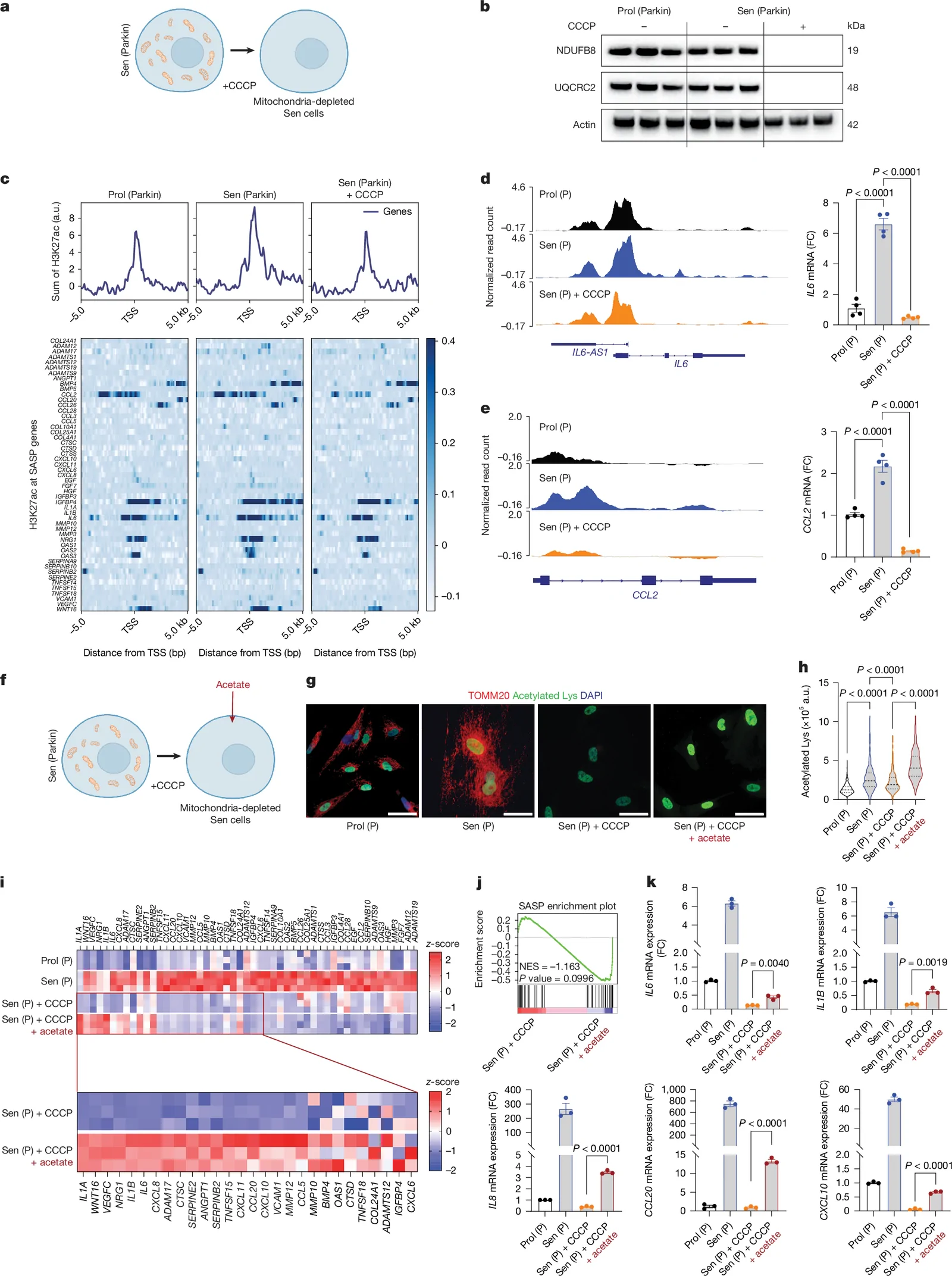
Mitochondria modulate histone acetylation at SASP loci. **a**, Schematic representation of the experimental setup. Sen, senescent cells. Created in BioRender; Martini, H. https://biorender.com/of88ogz (2026). **b**, Western blot showing mitochondrial proteins UQCRC2 and NDUFB8, confirming loss of mitochondria in IMR90 Parkin-expressing senescent cells following CCCP treatment (Sen + CCCP) (*n* = 3). Prol, proliferative cells. **c**, Metaplot (top) and heat map (bottom) of H3K27ac enrichment at SASP genes, demonstrating reduced histone acetylation in mitochondria-depleted senescent cells. Signals were normalized to total histone H3 across ±5 kb from transcription start sites (TSS). Average of *n* = 3. a.u., arbitrary units. **d**,**e**, Browser track (left) and mRNA expression (right) of *IL6* (**d**) and *CCL2* (**e**) genes, displaying H3K27ac distribution. qPCR data, *n* = 4. Parkin and (P) indicate Parkin-overexpressing cells. FC, fold change. **f**, Scheme of the experimental workflow. Created in BioRender; Martini, H. https://biorender.com/of88ogz (2026). **g**, Representative immunofluorescence images of nuclear acetylated lysine and mitochondria staining (TOMM20). Scale bars, 50 μm. **h**, Quantification of nuclear acetylated lysine per cell from *n* = 3 independent experiments. **i**, Top, heat map of SASP genes in proliferative cells, senescent cells, senescent cells in the absence of mitochondria and after acetate treatment. Bottom, heat map of SASP genes that show increased expression in cells without mitochondria after acetate treatment. The colour intensity represents column *z*-score. *n* = 3. **j**, Gene set enrichment analysis (GSEA) enrichment plot of the 51 SASP genes, comparing senescent cells lacking mitochondria without and after acetate treatment. NES, normalized enrichment score. **k**, mRNA expression levels of various SASP genes (*n* = 3). Data are mean ± s.e.m. One-way ANOVA with Tukey’s multiple comparison test (**d**,**e**,**h**); weighted Kolmogorov–Smirnov-like test (**j**); or two-sided Student’s *t*-test (**k**). Senescence was induced by X-ray irradiation (IR). *n* represents the number of independent experiments. NS, not significant (*P* > 0.05). Source data

**Extended Data Fig. 1:**
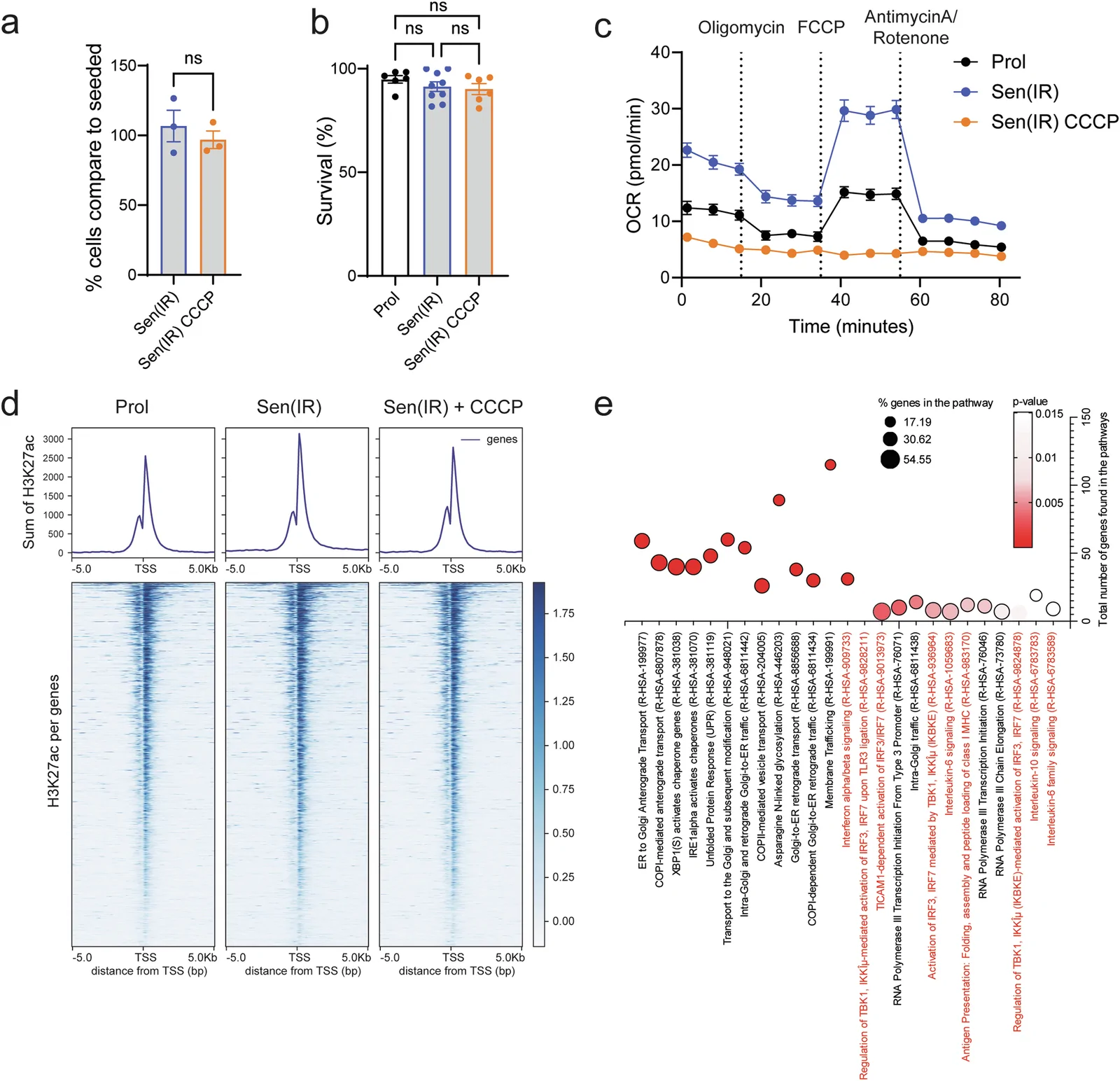
Mitochondrial control of histone acetylation is enriched in inflammatory pathways. (**a**) Percentage of cells day 12 post irradiation compared to the number of cells seeded at day 0 (n = 3). (**b**) % of survival cells assess by LDH assay (Prol n = 6, Sen(IR) n = 9, Sen(IR) + CCCP n = 6). (**c**) Seahorse analysis showing oxygen consumption rate (OCR) profiles (Prol n = 14, Sen(IR) n = 12, Sen(IR) + CCCP n = 12). (**d**) Metaplot and heatmap analysis of all the genes increasing in senescence (Sen(IR)) compared to proliferatives cell (Prol) and showing decreased histone acetylation (H3K27ac) in senescent cells lacking mitochondria (Sen(IR) + CCCP). Top: Metaplot depicting a composite sum of all normalized H3K27ac enrichment gene signals; Bottom: heatmap of H3K27ac enrichment normalized by total H3 (± 5 kb from TSS). Data are averaged from n = 3 independent experiments. (**e**) Reactome pathways of all the genes present in (d). In red, pathways related to inflammation. Data are expressed as Mean ± s.e.m. Two-sided Student’s t-test (a), One way ANOVA with Tukey’s multiple comparison test (b), Hypergeometric test with Benjamini–Hochberg FDR correction (e). (n = independent experiment). p-value as indicated in the figure, ns = non significant p > 0.05. Source data

To address this, we utilized a previously established model of mitochondrial clearance in which human fibroblasts overexpressing Parkin are treated with CCCP to induce mitophagy and generate cells depleted of mitochondria^16,17^ (Fig. 1a). Mitochondrial depletion did not affect senescent cell viability (Extended Data Fig. 1a,b), but abolished respiration and mitochondrial protein content (Fig. 1b and Extended Data Fig. 1c).

**Extended Data Fig. 2:**
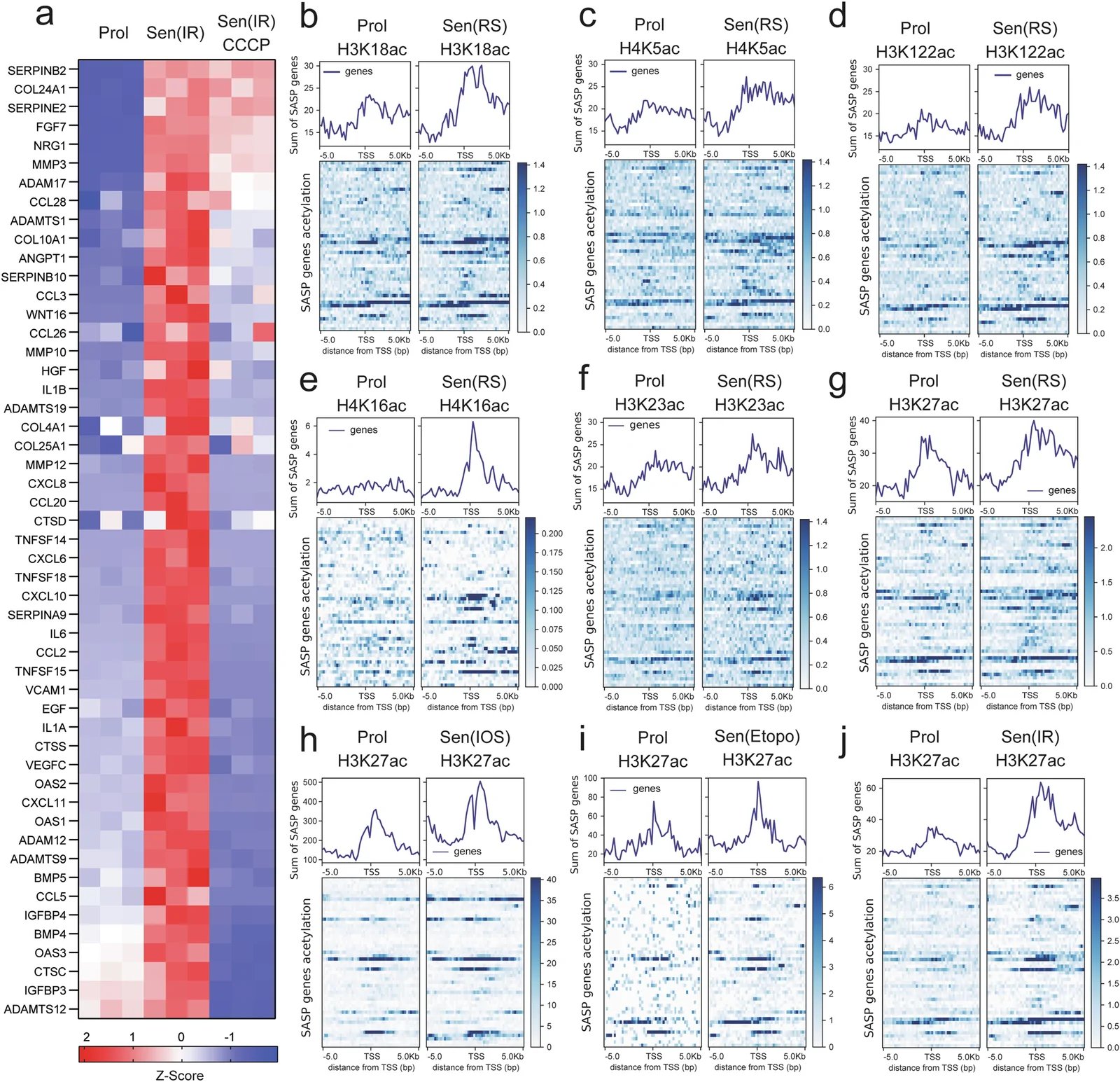
Mitochondria-dependent histone acetylation patterns at SASP genes. (**a**) Heatmap of 51 SASP genes differentially expressed in senescent IMR90 cells (Sen(IR)) and reduced following mitochondrial depletion via CCCP-induced mitophagy (Sen(IR) + CCCP). Color scale indicates column Z-score (red, high; blue, low expression) (n = 3 independant experiment). (**b-f**) Distribution of histone acetylation marks (H3K18ac, H3K5ac, H3K122ac, H4K16ac, and H3K23ac) at SASP genes in replicative senescent (RS) IMR90 fibroblasts. Top: Metaplot showing the composite signal across all 51 genes. Bottom: Heatmap of acetylation enrichment at individual genes (± 5 kb from transcription start sites, TSS). Data reanalyzed from GSE106146 and GSE56307. (**g-j**) H3K27ac enrichment at SASP genes in IMR90 cells induced into senescence by distinct triggers. Top: Metaplot showing the composite signal across all 51 genes. Bottom: Heatmap of acetylation patterns at individual genes (± 5 kb from TSS). Data reanalyzed from GSE74238, GSE103590, and GSE106146. Source data

Chromatin immunoprecipitation with sequencing (ChIP–seq) analysis of histone H3 acetylation at lysine 27 (H3K27ac) revealed that loci that gained acetylation during senescence were markedly reduced following mitochondrial clearance (Extended Data Fig. 1d). Pathway analysis showed that these regions were enriched for inflammatory and interleukin signalling pathways, central components of SASP (Extended Data Fig. 1e). Consistent with this, we identified a 51-gene SASP signature that was induced during senescence but suppressed upon mitochondrial removal (Extended Data Fig. 2a). H3K27ac enrichment at these loci was concordantly reduced following mitochondrial clearance (Fig. 1c), with representative genes such as *IL6* and *CCL2* showing coordinated decreases in chromatin acetylation and mRNA expression (Fig. 1d,e). These findings indicate that mitochondria are required to sustain histone acetylation at SASP genes.

Having established that H3K27ac at SASP loci is increased during senescence and reduced by mitochondrial clearance, we next explored whether this regulation extends across histone marks and senescence contexts. Analysis of multiple published ChIP–seq datasets spanning multiple acetylated histone marks and senescence models, including irradiation, etoposide, oncogene-induced and replicative senescence, revealed consistent enrichment of histone acetylation at SASP gene loci (Extended Data Fig. 2b–j).

**Extended Data Fig. 3:**
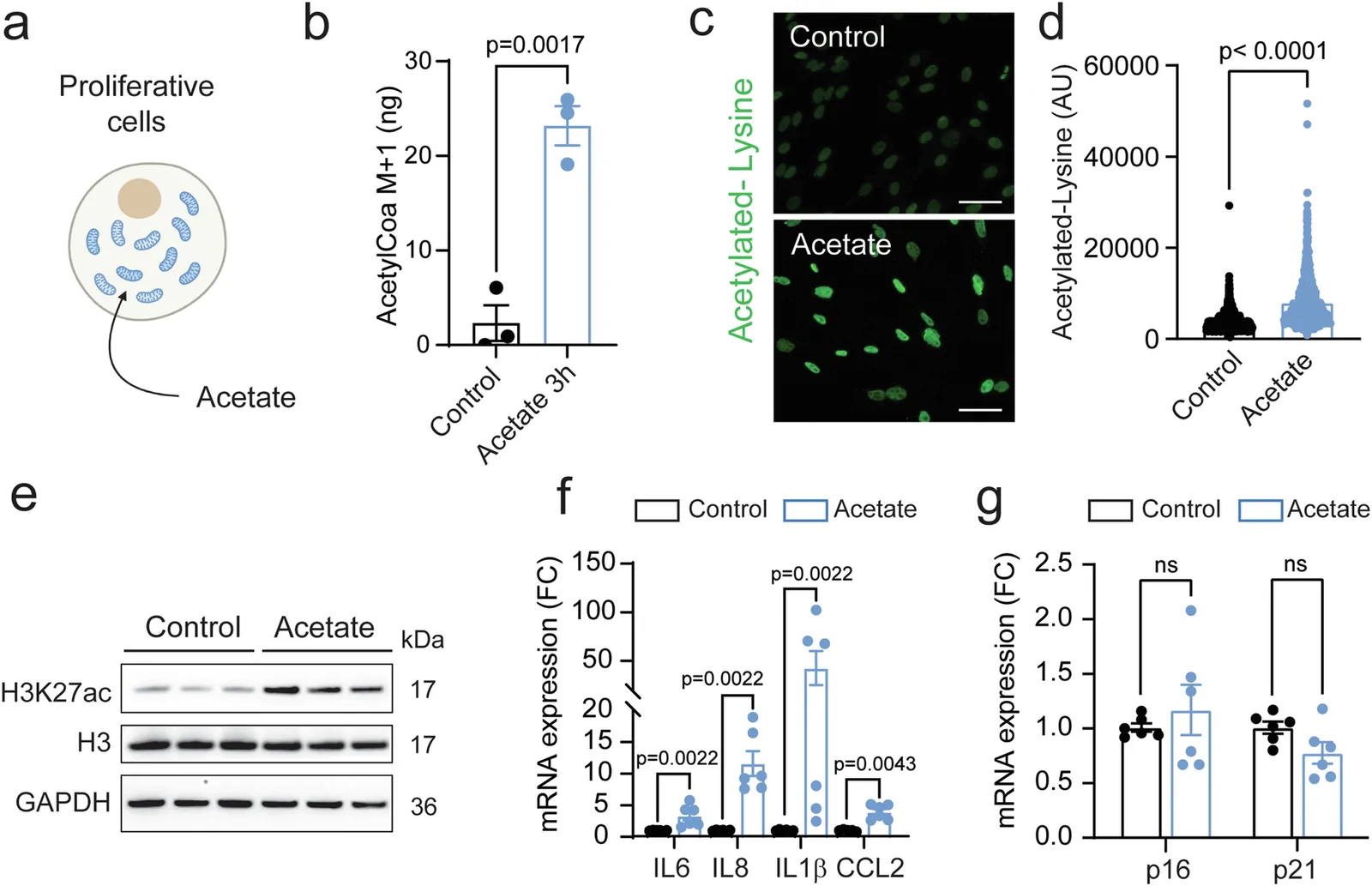
Exogenous acetate drives nuclear acetylation and expression of SASP genes. (**a**) Scheme of the experimental procedure. Created in BioRender; Passos, J. https://biorender.com/k32ksox (2026). (**b**) Incorporation of ^13^C-labeled acetate into acetyl-CoA (M + 1) measured 3 h after supplementation (n = 3). (**c**) Representative immunofluorescence images showing nuclear acetylated-lysine staining. (**d**) Quantification of nuclear acetylated-lysine (integrated density) per cell (from n = 3). (**e**) Western blot of histone acetylated (H3K27ac) and total histone H3, showing an increase of histone acetylation after acetate supplementation (n = 3). (**f-g**) mRNA expression of different SASP genes (**f**) and cell cycle regulators (*p16*, *p21*) (**g**) expressed in fold change compared to proliferative cells (Control) (n = 6). Data are expressed as Mean ± s.e.m. Statistical tests: Two-sided Student’s t-test (b), Two-tailed Mann Whitney test (d, f, g). (n = independent experiment). P-values as indicated in the figure. ns = non significant p > 0.05. Source data

Given that mitochondrial clearance suppresses histone acetylation at SASP genes, we next tested whether this effect reflects reduced availability of acetyl-CoA. Because acetyl-CoA is not membrane permeable, we supplemented cells with acetate (Extended Data Fig. 3a), which is converted intracellularly into acetyl-CoA by ACSS2 (ref. ^18^). Acetate isotope tracing experiments confirmed rapid incorporation of exogenous acetate into acetyl-CoA (Extended Data Fig. 3b). Moreover, acetate supplementation increased global nuclear acetylation, including H3K27ac (Extended Data Fig. 3c–e), and induced expression of pro-inflammatory genes, including *IL6*, *IL8*, *IL1B* and *CCL2*, without affecting cell cycle regulators p16 (also known as *CDKN2A*) and p21 (also known as *CDKN1A*) (Extended Data Fig. 3f,g).

To test whether acetyl-CoA is sufficient to restore SASP expression in the absence of mitochondria, we supplemented mitochondria-depleted senescent cells with acetate (Fig. 1f). Acetate supplementation increased histone acetylation (Fig. 1g,h) and partially restored a subset of the mitochondrial-dependent SASP programme, as determined by RNA sequencing (RNA-seq) and quantitative PCR (qPCR) (Fig. 1i–k).

Together, these findings demonstrate that mitochondria regulate SASP gene expression through acetyl-CoA-dependent histone acetylation, establishing acetyl-CoA as a key metabolic link between mitochondria and epigenetic regulation of inflammatory genes.

## Mitochondrial citrate–acetyl–CoA axis drives SASP

Having established that acetyl-CoA is sufficient to promote histone acetylation and SASP gene expression, and that mitochondria are required for this process, we next investigated how mitochondrial metabolic pathways contribute to this regulation.

**Extended Data Fig. 4:**
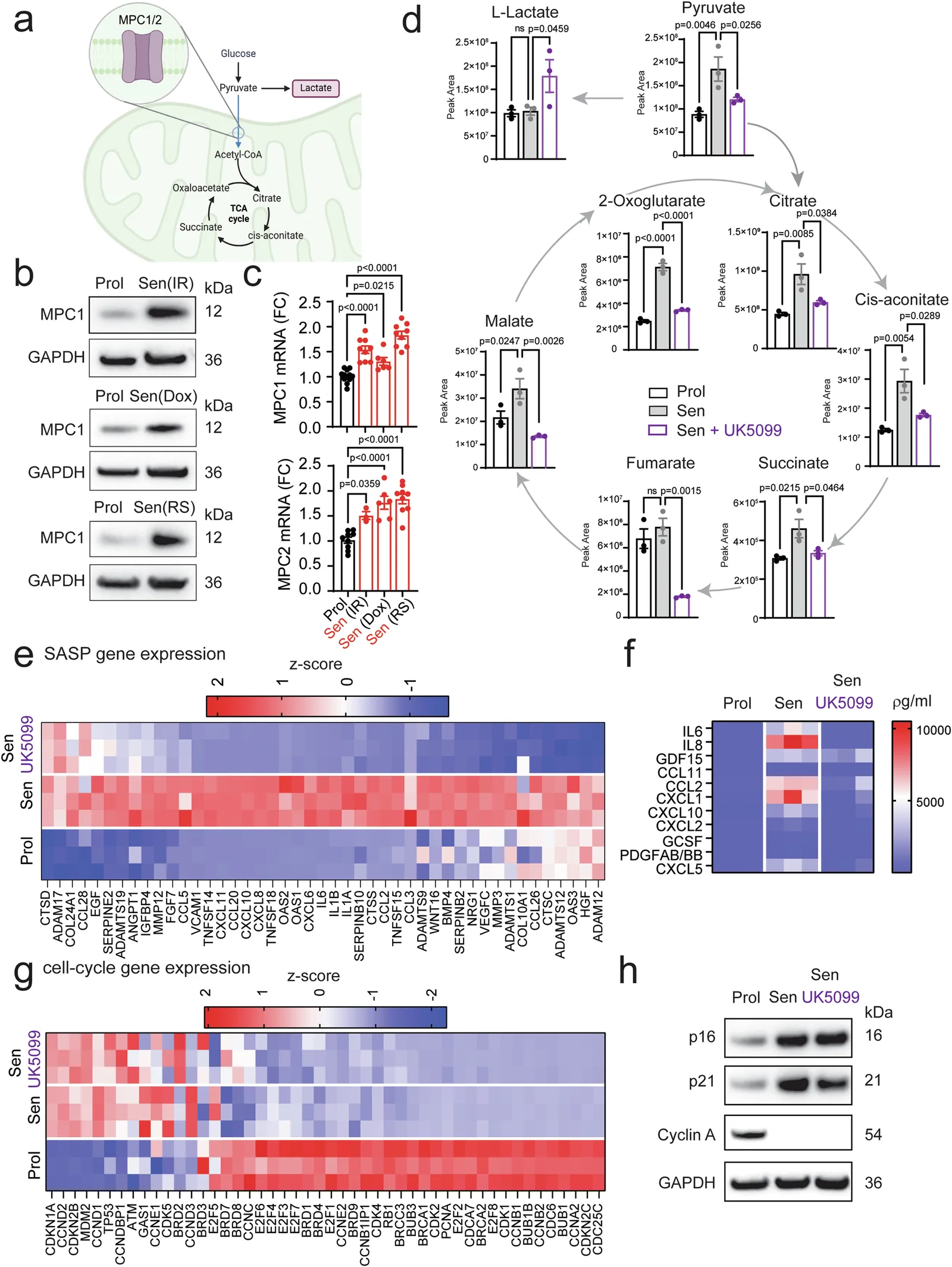
Upregulation of mitochondrial pyruvate carriers (MPC) in senescence modulates the SASP. (**a**) Schematic illustration of MPC localization and function within the mitochondria. Created in BioRender; Passos, J. https://biorender.com/t6ywwdm (2026). (**b**) Western blot analysis showing increased MPC1 protein levels in different models of senescence: irradiation-induced (Sen(IR)), doxorubicin-induced (Sen(Dox)), and replicative senescence (Sen(RS)). (**c**) mRNA expression levels of *MPC1* and *MPC2* across senescence models (MPC1: Prol n = 12, Sen(IR) n = 9, Sen(Dox) n = 6, Sen(RS) n = 9; MPC2: Prol n = 9, Sen(IR) n = 3, Sen(Dox) n = 6, Sen9RS) n = 9). (**d**) Mass spectrometry quantification of TCA cycle metabolites in senescent cells following treatment with UK5099 (MPC inhibitor) (n = 3) (**e**) Column-clustered heatmap showing differential expression of SASP genes in senescent cells, showing downregulation following UK5099 treatment. Color intensity represents column Zscores (red: high expression, blue: low expression). (**f**) Cytokine array heatmap of 24-h conditioned media from senescent cells, showing cytokine expression changes. (**g**) Column-clustered heatmap of cell cycle-associated genes in senescent cells, which are not affected by UK5099 treatment (color intensity as in panel e). (**h**) Western blot indicating that UK5099 does not reduce expression of p16 and p21, nor restore Cyclin A levels, confirming a lack of effect on cell cycle arrest. For RNA-seq and cytokine arrays, n = 3 independent conditions were analyzed. Data are presented as Mean ± s.e.m Statistical tests: One-way ANOVA with Tukey’s multiple comparison test or uncorrected Fisher’s LSD. (n = independent experiment). P-values as indicated in the figure. ns = non significant p > 0.05. Source data

**Extended Data Fig. 5:**
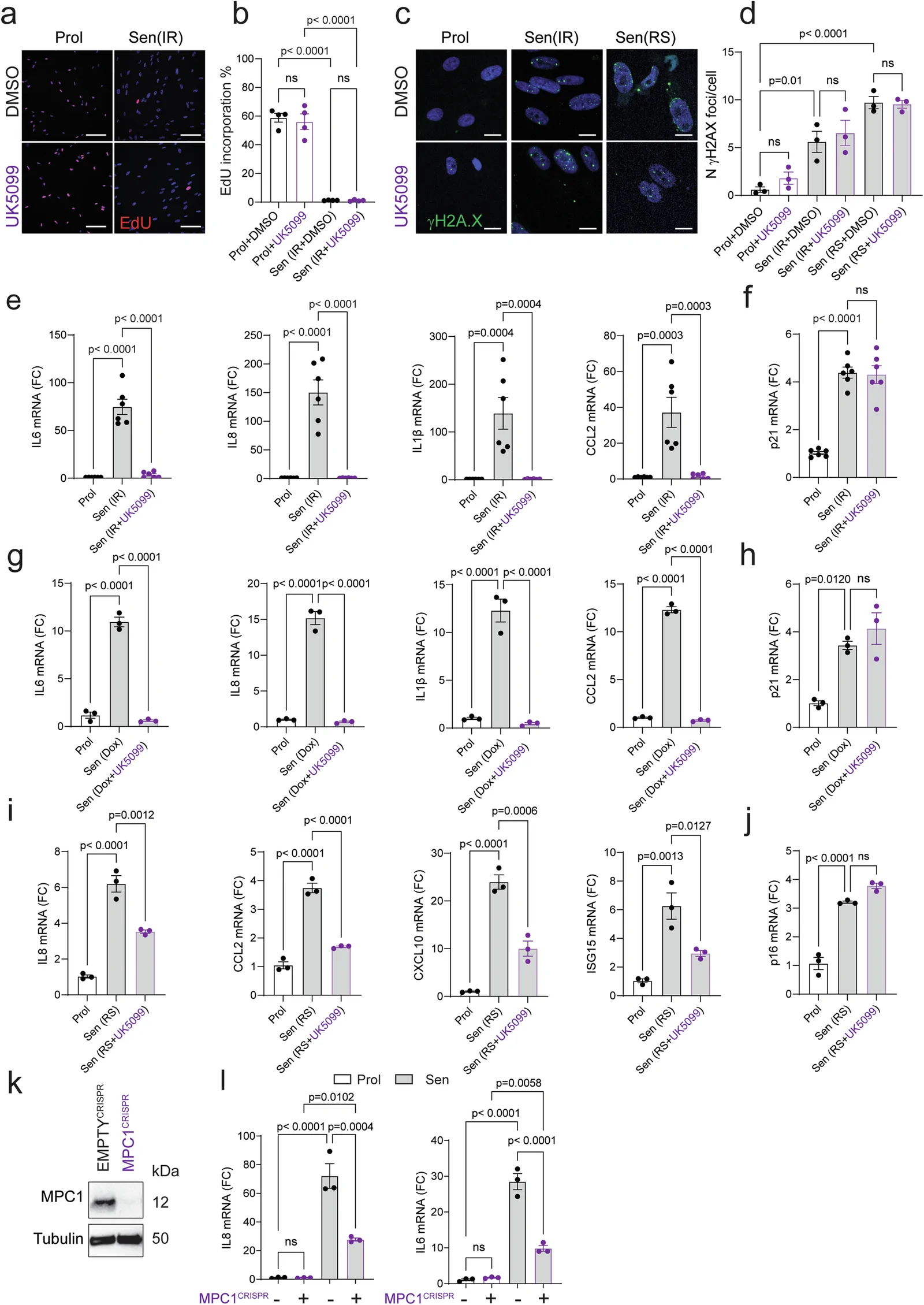
MPC inhibition reduces the SASP in multiple senescence models without affecting γH2A.X foci or cyclin-dependent kinase inhibitors. (**a**) Representative immunofluorescence images of EdU (red). Scale bar = 200 μm. (**b**) Quantification of EdU-positive cells per field in proliferative (Prol) and senescent cells induced by irradiation (Sen(IR)), with or without the MPC inhibitor UK5099 (n = 4). (**c**) Representative immunofluorescence images of γH2A.X foci staining. Sacle bar = 20 μm (**d**) Quantification of γH2A.X foci per nucleus in proliferative (Prol), irradiation-induced senescent (Sen(IR)), and replicative senescent (Sen(RS)) cells treated with or without UK5099 (n = 3). (**e-j**) mRNA expression levels of various SASP genes (**e, g, i**) and cyclin-dependent kinase inhibitors (CDKi) (**f, h, j**) expressed as fold change relative to proliferative cells (Prol) under different senescence inducers and following UK5099 treatment: irradiation-induced senescence (Sen(IR)) (n = 6) (**e, f**), doxorubicin-induced senescence (Sen(Dox)) (n = 3) (**g, h**), and replicative senescence (Sen(RS)) (n = 3) (**i, j**). (**k**) Western blot showing successful CRISPR-Cas9-mediated deletion of MPC1. (**l**) mRNA expression of *IL6* and *IL8* in MPC1 knock-out cells, presented as fold change relative to proliferative cells (Prol) (n = 3). Data are shown as Mean ± s.e.m. Statistical tests: One-way ANOVA with Tukey’s multiple comparison test. (n = independent experiment). P-values as indicated in the figure. ns = non significant p > 0.05. Source data

We first examined the mitochondrial pyruvate carrier (MPC), which links glycolysis-derived pyruvate to the TCA cycle (Extended Data Fig. 4a). Expression of MPC1 and MPC2 were increased across multiple senescence models, including irradiation-induced, doxorubicin-induced and replicative senescence (Extended Data Fig. 4b,c). Pharmacological inhibition of MPC with UK5099 reduced TCA cycle intermediates, which are upregulated in senescence, and increased lactate levels, consistent with impaired mitochondrial pyruvate import (Extended Data Fig. 4d). RNA-seq and cytokine profiling revealed that MPC inhibition suppressed SASP gene expression and secretion (Extended Data Fig. 4e,f), without affecting cell cycle arrest, as assessed by proliferation-associated gene expression, p16 and p21 levels, EdU incorporation and DNA damage foci (Extended Data Figs. 4g,h and 5a–d,f). Similar effects were observed across distinct senescence models (Extended Data Fig. 5e,g–j). Consistent with these findings, CRISPR–Cas9-mediated deletion of MPC1 reduced expression of SASP factors such as *IL6* and *IL8* (Extended Data Fig. 5k,l).

**Fig. 2:**
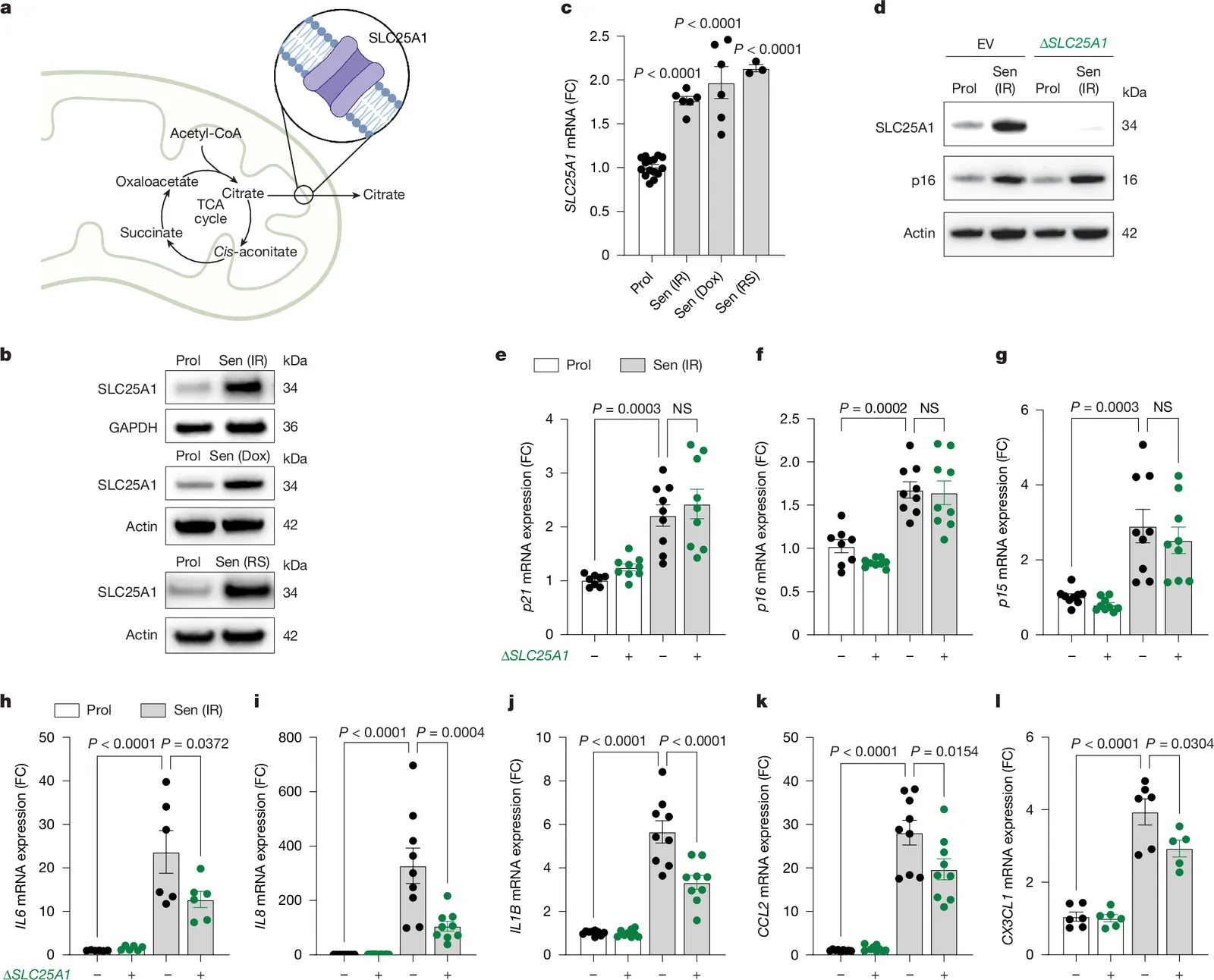
Upregulation of SLC25A1 modulates SASP in senescent cells. **a**, Schematic illustration of SLC25A1 localization and function in mitochondria. Created in BioRender; Passos, J. https://biorender.com/zq5sona (2026). **b**, Western blot showing increased SLC25A1 protein in senescence models. Sen (Dox) indicates doxorubicin-induced senescence; Sen (RS) indicates replicative senescence. **c**, mRNA expression of *SLC25A1* in these senescence models compared to in proliferative cells. Prol, *n* = 15; Sen (IR), *n* = 6; Sen (Dox), *n* = 6; Sen (RS), *n* = 3. **d**, Western blot confirming successful CRISPR–Cas9-mediated deletion of *SLC25A1* (Δ* SLC25A1*) in proliferative (Prol) and senescent (Sen (IR)) cells, with persistent p16 expression in senescent cells after knockout. EV, empty vector. **e**–**g**, Expression of mRNA encoding the CDK inhibitors p21 (**e**), p16 (**f**) and p15 (**g**) in irradiation-induced senescent cells with or without *SLC25A1*. *n* = 9. **h**–**l**, Expression of mRNA encoding the SASP components *IL6* (**h**), *IL8* (**i**), *IL1B* (**j**), *CCL2* (**k**) and *CX3CL1* (**l**) under the same conditions. Data are presented as fold change relative to proliferative cells (Prol). *n* = 6 (**h**); *n* = 9 (**i**–**k**); *n* = 6 (**l**). Data are mean ± s.e.m. One-way ANOVA with Tukey’s multiple comparison test. Source data

**Fig. 3:**
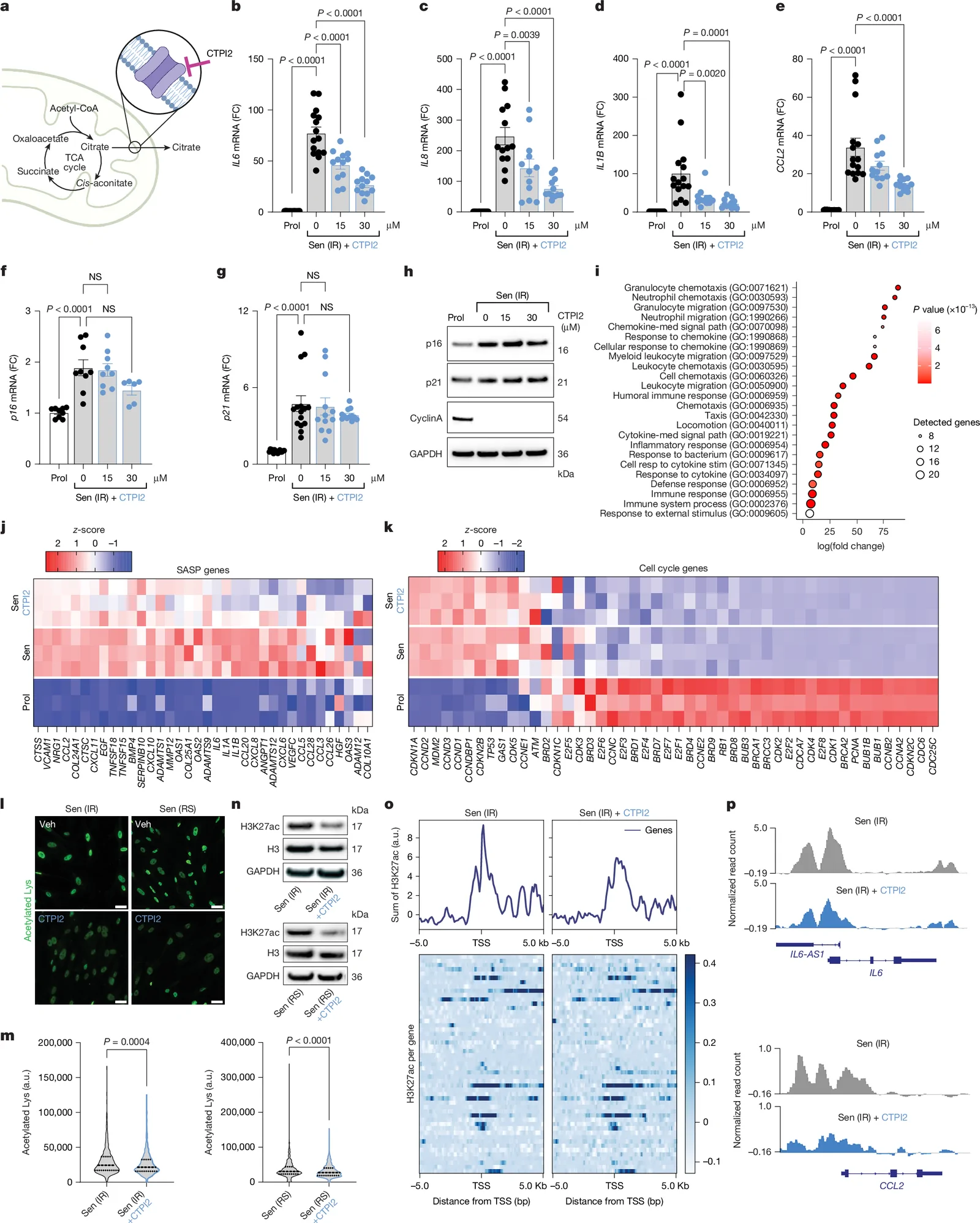
Pharmacological inhibition of SLC25A1 reduces SASP in senescent cells. **a**, Schematic illustrating the experimental approach. Created in BioRender; Passos, J. https://biorender.com/zq5sona (2026). **b**–**g**, Expression of mRNA encoding the SASP components IL6 (**b**), IL8 (**c**), IL1B (**d**) and CCL2 (**e**) and the CDK inhibitors p16 (**f**) and p21 (**g**) in irradiation-induced senescent cells treated with 15 μM or 30 μM CTPI2, expressed as fold change relative to proliferative cells. **b**–**e**,**g**, Prol, *n* = 15; Sen (IR), *n* = 15; Sen (IR) 15 μM, *n* = 12; Sen (IR) 30 μM, *n* = 12. **f**, Prol, *n* = 9; Sen (IR), *n* = 9; Sen (IR) 15 μM, *n* = 9; Sen (IR) 30 μM, *n* = 6. **h**, Western blot analysis showing that CTPI2 treatment does not alter expression of p16 or p21, and does not restore cyclin A levels. **i**, Reactome pathways of genes downregulated by CTPI2 compared to in untreated senescent cells. GO, Gene Ontology. **j**,**k**, Column-clustered heat maps showing SASP factor genes (**j**) and cell cycle-associated genes (**k**). Colour intensity represents column *z*-scores; *n* = 3. **l**, Representative immunofluorescence images of nuclear acetylated lysine in two models of senescence treated with or without CTPI2. Veh, vehicle. Scale bars, 50 μm. **m**, Quantification of integrated nuclear acetylated lysine density per cell. *n* = 3. **n**, Western blots showing reduced H3K27ac after CTPI2 treatment with irradiation-induced (top) or replicative (bottom) senescence. **o**, Metaplot (top) and heat map (bottom) of SASP genes showing reduced histone H3K27ac levels normalized to histone H3 around the transcription start sites (±5 kb) in irradiation-induced senescent after CTPI2 treatment (data averaged from *n* = 3). **p**, Browser tracks of *IL6* (top) and *CCL2* (bottom) genes, depicting reduced H3K27ac levels following CTPI2 treatment. Data are mean ± s.e.m. One-way ANOVA with Tukey’s multiple comparison test (**b**–**g**); hypergeometric test with Benjamini–Hochberg false discovery rate correction (**i**); two-tailed Mann–Whitney test (**m**). *n* represents the number of independent experiments. Source data

**Extended Data Fig. 6:**
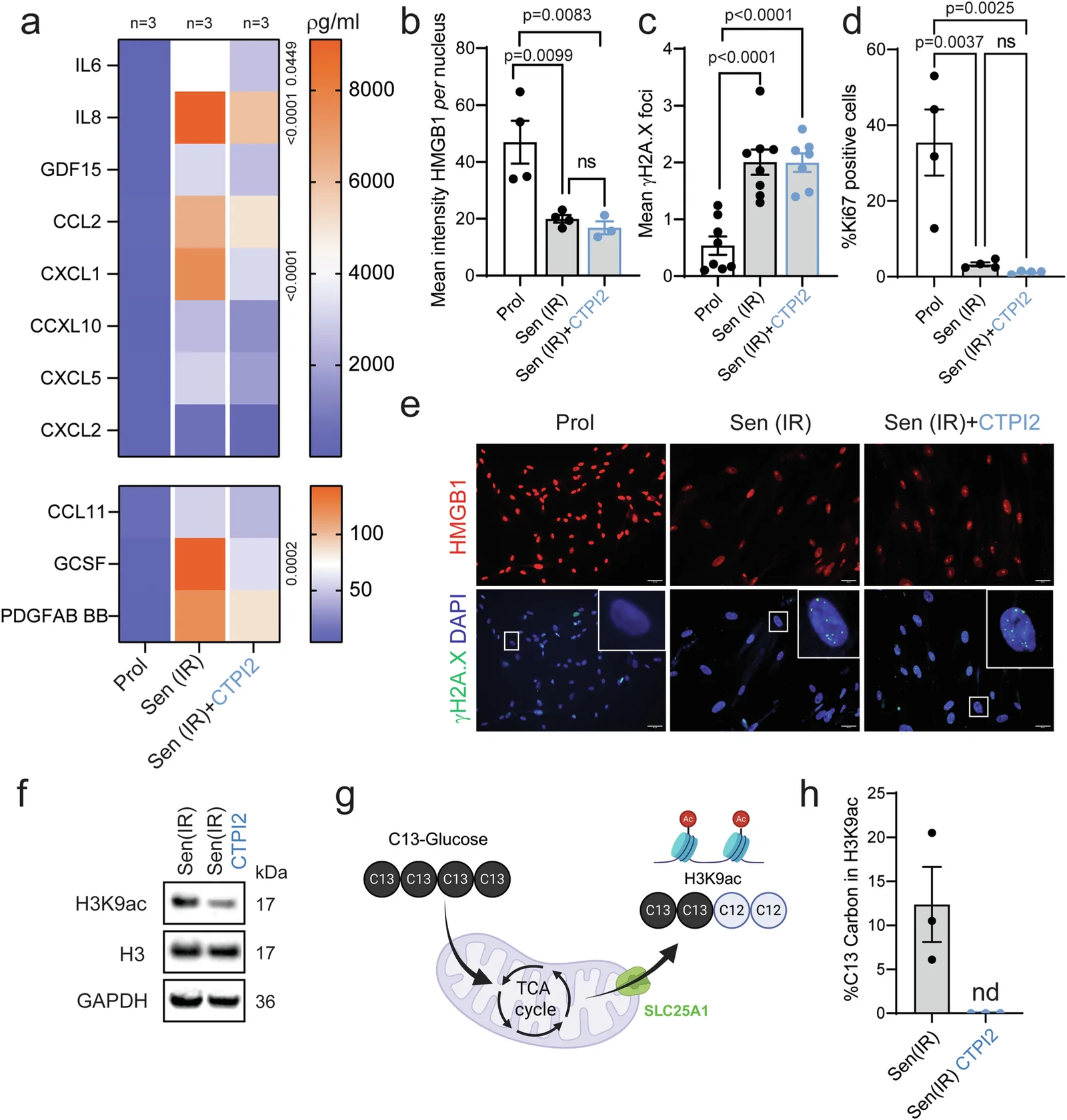
Pharmacological inhibition of Mitochondrial Citrate Carrier (SLC25A1) reduces the SASP without affecting other hallmarks of senescence. (**a**) Heatmap of cytokine array data from 24-h conditioned media, presented as the average of n = 3 independent experiments per condition. (**b-d**) Quantification of (**b**) nuclear HMGB1 mean intensity per nucleus, (**c**) mean number of γH2A.X foci per nucleus, and (**d**) percentage of Ki67-positive proliferating cells in proliferative, senescent, and CTPI2-treated senescent cells ((b) n = 4 Prol and Sen(IR), n = 3 Sen(IR) + CTPI2, (c) n = 8, (d) n = 4). (**e**) Representative immunofluorescence images of HMGB1 and γH2A.X staining in different conditions. Scale bar = 50 μm. (**f**) Western blot showing reduced histone acetylation (H3K9ac) following CTPI-2 treatment (representative from n = 3). (**g**) Schematic illustrating the ^13^C-glucose tracing experimental approach. Created in BioRender; Martini, H. https://biorender.com/u87b9lc (2026). (**h**) Percentage of ^13^C incorporation into H3K9ac after 24 h in irradiation-induced senescent cells (Sen(IR)) treated with or without CTPI-2 (n = 3). nd, not detected. Data are presented as mean ± s.e.m. Statistical analyses were performed using one-way ANOVA with Tukey’s multiple comparison test. (n = independent experiment). P-values as indicated in the figure. ns = non significant p > 0.05. Source data

**Extended Data Fig. 7:**
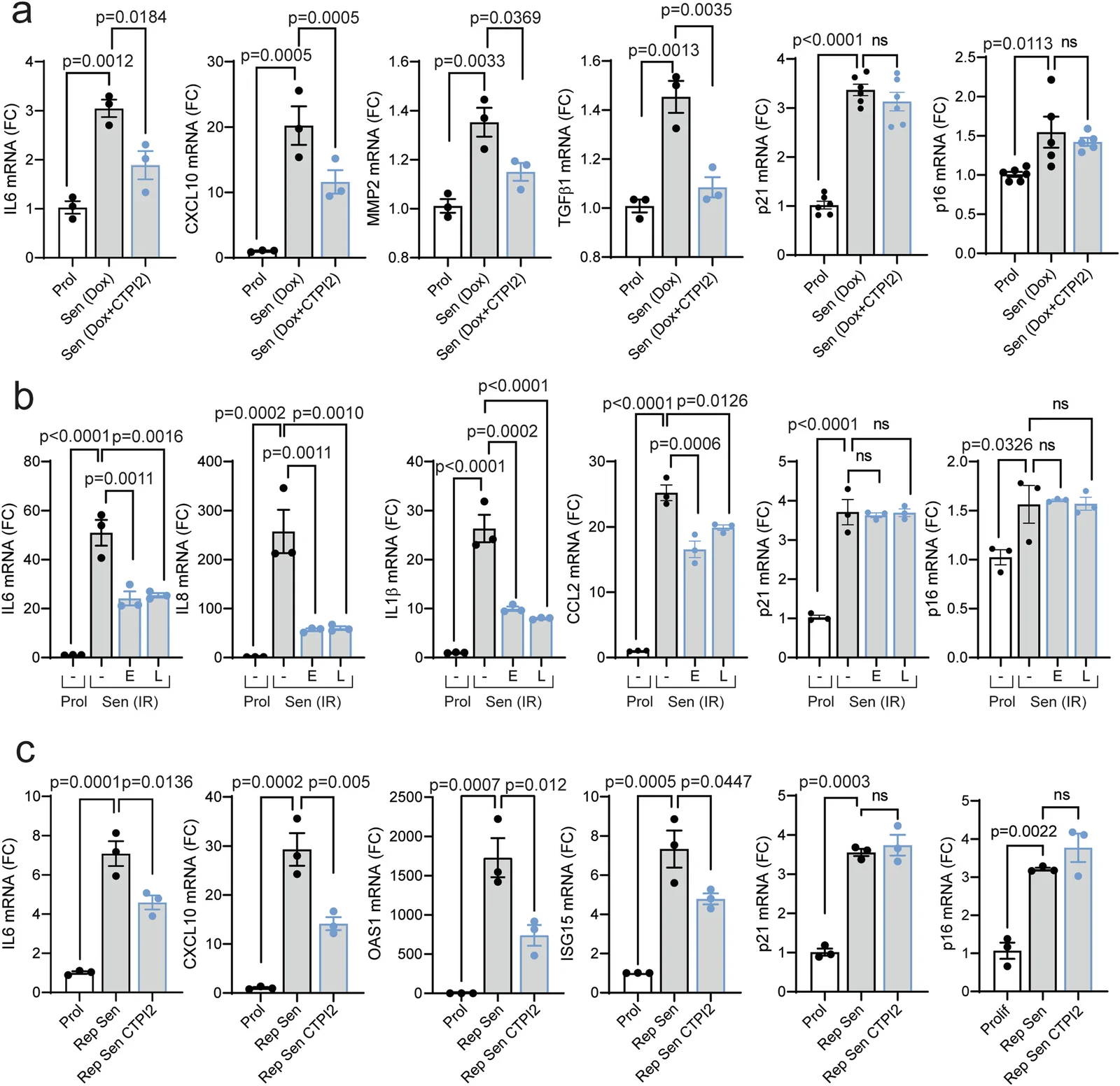
Pharmacological inhibition of the mitochondrial citrate carrier (SLC25A1) reduces the SASP across multiple senescence models. (**a**) Relative mRNA expression of SASP factors and cyclin-dependent kinase inhibitors (CDKIs) in doxorubicin-induced senescence (Sen(Dox)) with or without CTPI2 treatment (30 μM), expressed as fold change relative to proliferating cells (Prol). (**b**) Relative mRNA expression of SASP factors and CDKIs in irradiation-induced senescence (Sen(IR)) following early (day 2 post-irradiation) or late (day 8 post-irradiation) CTPI-2 treatment (30 μM), expressed as fold change relative to proliferating cells (Prol). (**c**) Relative mRNA expression of SASP factors and CDKIs in replicative senescence (Sen(RS)) with or without CTPI-2 treatment (30 μM), expressed as fold change relative to proliferating cells (Prol). Data represent mean ± s.e.m. from three independent experiments. Statistical significance was assessed using one-way ANOVA with Tukey’s multiple comparison test. P-values as indicated in the figure. ns = non significant p > 0.05. Source data

We next focused on the mitochondrial citrate carrier SLC25A1, which exports citrate to the cytosol, where it can be converted to acetyl-CoA^19^ (Fig. 2a). SLC25A1 expression was significantly increased at both mRNA and protein levels in multiple senescence contexts (Fig. 2b,c). Genetic ablation of SLC25A1 did not alter expression of CDK inhibitors or cell cycle arrest (Fig. 2d–g), but led to a marked reduction in SASP gene expression (Fig. 2h–l). Similarly, pharmacological inhibition of SLC25A1 using CTPI2, a third-generation inhibitor with high binding affinity and specificity^20^ (Fig. 3a), resulted in a dose-dependent suppression of SASP components at both the transcript and protein levels (Fig. 3b–e and Extended Data Fig. 6a). This effect occurred without alterations in cell cycle regulators, HMGB1 localization, DNA damage foci or proliferation markers (Fig. 3f–h and Extended Data Fig. 6b–e). RNA-seq and pathway analysis confirmed that CTPI2 downregulated inflammatory and mitochondrial-dependent SASP programmes, but had no effect on cell cycle-associated gene expression (Fig. 3i–k). These effects were consistent across senescence models and treatment windows, including treatment initiated shortly after senescence induction as well as in fully established senescent cells (Extended Data Fig. 7a–c).

**Extended Data Fig. 8:**
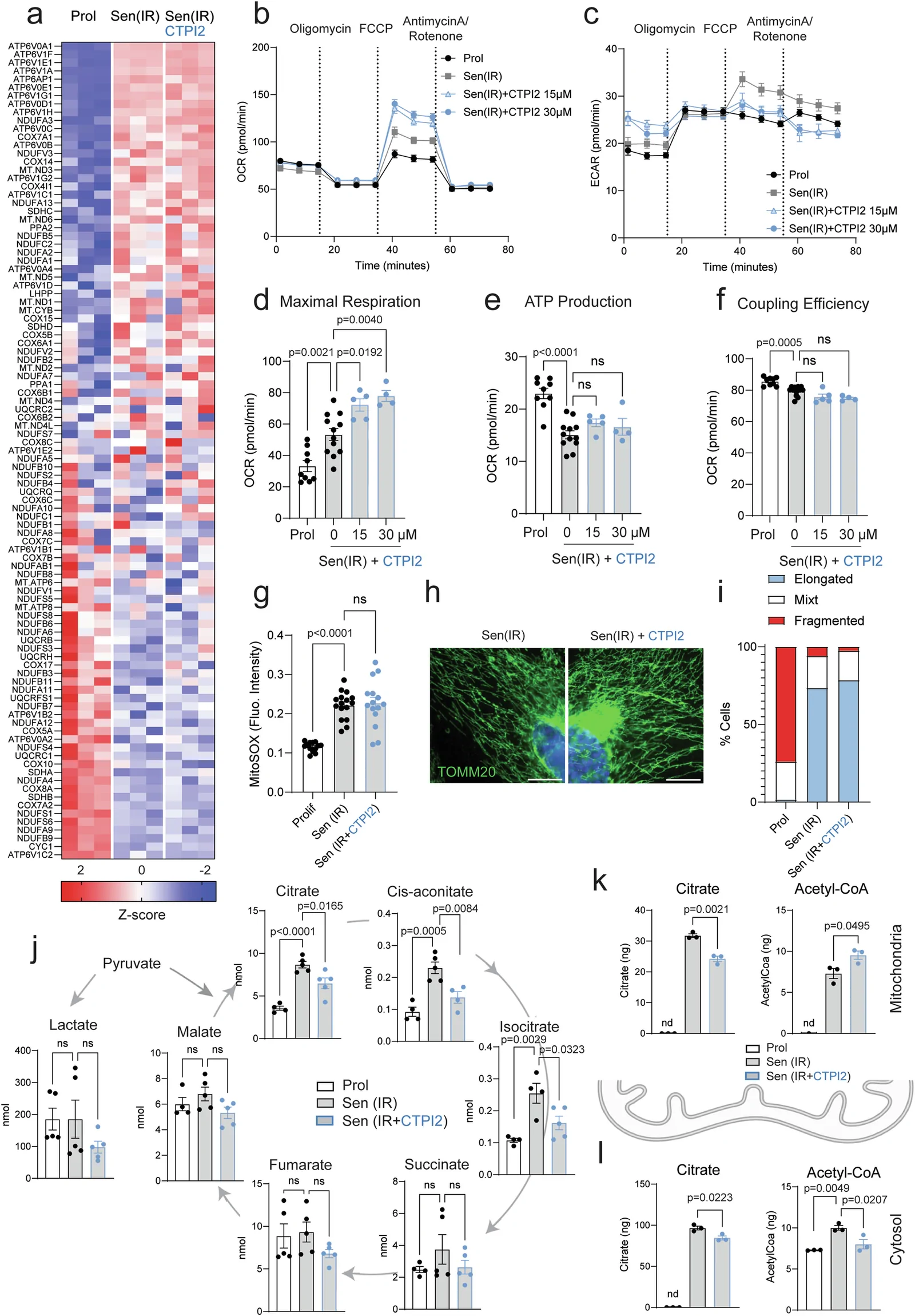
Effects of Mitochondrial Citrate Carrier (SLC25A1) inhibition on mitochondrial metabolism and function in senescent cells. (**a**) Clustered heatmap of oxidative phosphorylation (OXPHOS) genes that are differentially expressed in senescent cells and unaffected by CTPI2 treatment. The color intensity represents the column Z-score (n = 3). (**b, c**) Seahorse analysis showing oxygen consumption rate (OCR) (**b**) and extracellular acidification rate (ECAR) (**c**) profiles. (**d-f**) Quantification of various OCR parameters: maximal respiration (**d**), ATP production (**e**), and coupling efficiency (**f**). (**g**) MitoSOX fluorescence intensity, indicating mitochondrial reactive oxygen species (ROS) levels (Prol n = 13, Sen(IR) n = 16, Sen(IR) CTPI2 n = 15).(**h**) Representative immunofluorescence images of the mitochondrial network (TOMM20, green) in senescent cells treated with or without CTPI2. Scale bar 20 μm (**i**) Quantification of cells displaying fragmented, mixed, or elongated mitochondrial morphology. (**j**) TCA cycle metabolite levels measured in proliferative and senescent cells with or without CTPI2 treatment, expressed in nmol per 5 million cells (Prol n = 4, Sen(IR) n = 5, Sen(IR) CTPI2 n = 5). (**k-l**) Citrate and AcetylCoA measurement in the mitochondrial (**k**) or cytosolic (**l**) fraction in proliferative and senescent cells treated or not by CTPI2 (n = 3). Data are shown as Mean ± s.e.m. Drawing created in BioRender; Martini, H. https://biorender.com/5ty77zv (2026). For panel b-f, Prol n = 9, Sen(IR) 0 μm n = 12, Sen(IR) 15 μm n = 5, Sen(IR) 30 μm n = 4. Statistical tests: One-way ANOVA with Tukey’s multiple comparison test (**d**-**g**,**j**,**l**) or Two-sided Student’s t-test (**k**,**l**). (n = independent experiment). P-values as indicated in the figure. ns = non significant p > 0.05. Source data

Mechanistically, SLC25A1 inhibition reduced global nuclear acetylation, including pan-acetylated lysine staining and H3K27ac levels (Fig. 3l–n), and decreased H3K27ac at SASP loci as determined by ChIP–seq (Fig. 3o,p). Additional histone marks, including H3K9ac, were similarly reduced. Metabolic tracing demonstrated that incorporation of glucose-derived carbon into histone acetylation was abolished upon SLC25A1 inhibition (Extended Data Fig. 6f–h). Notably, CTPI2 did not broadly impair mitochondrial function, as oxidative phosphorylation (OXPHOS) gene expression, ATP production, coupling efficiency, mitochondrial reactive oxygen species levels and mitochondrial morphology were largely unchanged (Extended Data Fig. 8a–i). Metabolomic analysis revealed that inhibition of SLC25A1 reduced citrate, isocitrate and *cis*-aconitate in whole-cell extracts (Extended Data Fig. 8j). As SLC25A1 mediates citrate export to the cytosol, we performed subcellular fractionation and found that acetyl-CoA levels were selectively reduced in the cytosolic fraction (Extended Data Fig. 8k,l), indicating impaired generation of nucleocytosolic acetyl-CoA required for histone acetylation.

**Fig. 4:**
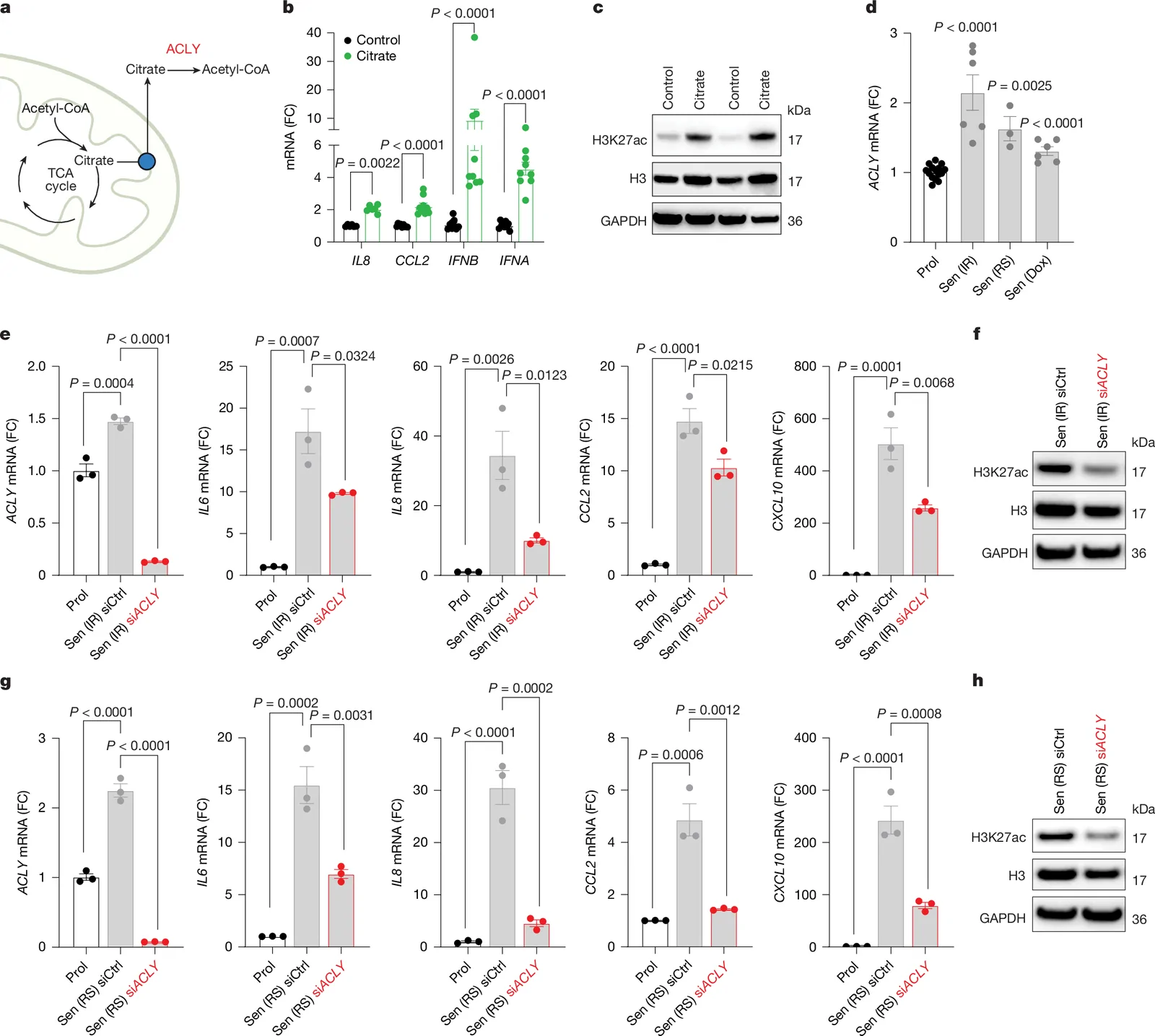
Mitochondria-derived citrate drives SASP through ACLY activity. **a**, Schematic representation illustrating the export of citrate from mitochondria and conversion to acetyl-CoA. Created in BioRender; Passos, J. https://biorender.com/zq5sona (2026). **b**, Relative mRNA expression of SASP components in proliferative cells treated with 10 mM citrate compared to in untreated proliferative cells (control), shown as fold change. *n* = 9. **c**, Western blot analysis of H3K27ac and total histone H3, demonstrating increased histone acetylation in citrate-treated cells. **d**, Relative mRNA expression of *ACLY* in cells undergoing senescence induced by various stimuli. Prol, *n* = 9; Sen (IR), *n* = 6; Sen (RS), *n* = 3; Sen (Dox), *n* = 6. **e**, mRNA expression in irradiation-induced senescence, showing successful *ACLY* knockdown by small interfering RNA (siRNA) and subsequent effects on genetic expression of SASP components, expressed as fold change relative to proliferative cells. *n* = 3. **f**, Western blot analysis showing reduced H3K27ac following ACLY silencing in irradiation-induced senescent cells. **g**, mRNA expression in replicative senescence, showing effective *ACLY* knockdown by siRNA and effects on SASP gene expression, presented as fold change relative to proliferative cells. *n* = 3. **h**, Western blot analysis indicating decreased H3K27ac in cells displaying replicative senescence, following *ACLY* silencing. Data are mean ± s.e.m. Two-sided Mann–Whitney test (**b**,**d**); two-sided Student’s *t*-test (**d** (Dox)); one-way ANOVA with Tukey’s multiple comparison test (**e**,**g**). *n* represents the number of independent experiments. Source data

Finally, we assessed the downstream conversion of mitochondria-derived citrate into acetyl-CoA via ATP-citrate lyase (ACLY) (Fig. 4a). Because citrate exported from mitochondria serves as the principal substrate for cytosolic acetyl-CoA production, we tested whether increasing citrate availability is sufficient to drive histone acetylation and inflammatory gene expression. Citrate supplementation increased histone acetylation and induced pro-inflammatory gene expression in proliferating cells (Fig. 4b,c). *ACLY* expression was increased across multiple senescence models (Fig. 4d), and its silencing reduced both H3K27ac levels and SASP gene expression in irradiation-induced and replicative senescence (Fig. 4e–h).

Together, these findings identify the mitochondrial citrate–acetyl-CoA axis as a central regulator of SASP gene expression. Multiple nodes within this pathway, from pyruvate import to citrate export and acetyl-CoA generation, converge to control histone acetylation at SASP loci, thereby selectively modulating inflammatory gene expression without affecting the senescence-associated cell cycle arrest.

## mtDNA and metabolism co-regulate SASP

Having established that mitochondrial metabolism regulates SASP gene expression through acetyl-CoA-dependent histone acetylation, we next explored whether this pathway intersects with mtDNA-driven innate immune signalling, a known regulator of SASP. Cytosolic DNA species activate cGAS–STING signalling in senescent cells^21^, and mtDNA leakage has emerged as a key mitochondrial input driving inflammatory programmes during senescence and ageing^3,12,13^. We therefore examined whether mitochondrial regulation of histone acetylation and mtDNA–cGAS–STING signalling are mechanistically linked or represent distinct inputs into SASP control.

**Fig. 5:**
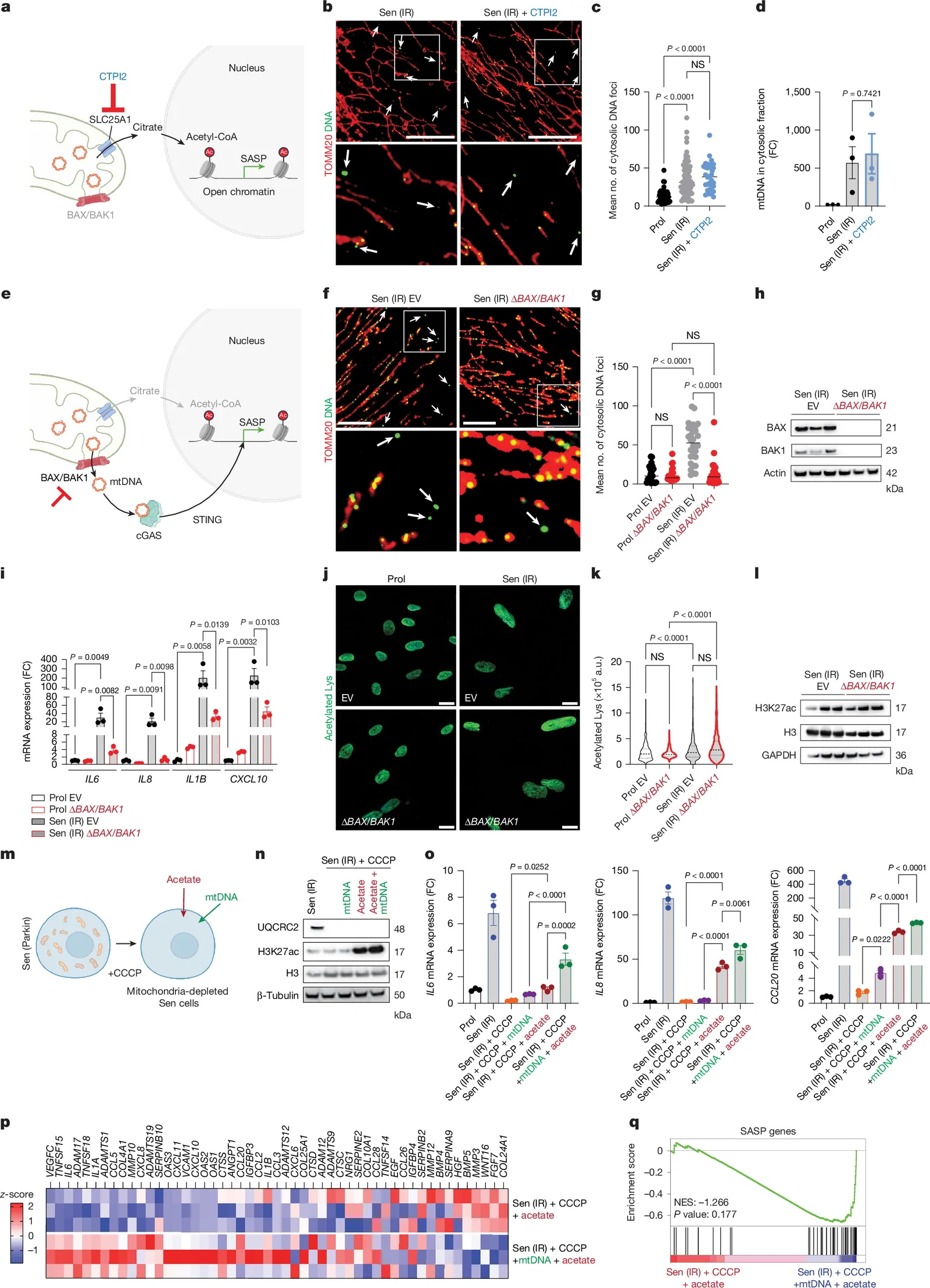
Mitochondrial citrate metabolism and mtDNA–cGAS–STING signalling independently regulate SASP. **a**, Schematic of mitochondrial citrate export influencing histone acetylation. Created in BioRender; Passos, J. https://biorender.com/uvwu372 (2026). **b**, Representative immunofluorescence images of mitochondrial network (TOMM20) and DNA staining in MRC5 cells. Arrows indicate DNA foci outside mitochondria. Scale bars, 20 μm. **c**, Quantification of DNA foci outside the mitochondrial network per cell. *n* = 3. **d**, Cytosolic mtDNA levels expressed as fold change compared with proliferative cells. *n* = 3. **e**, Schematic illustrating mtDNA leakage and activation of the cGAS–STING pathway driving SASP expression. Created in BioRender; Passos, J. https://biorender.com/uvwu372 (2026). **f**, Immunofluorescence of the mitochondrial network (TOMM20) and DNA staining with empty vector or BAX and BAK1 knockout (Δ*BAX/BAK1*). Arrows indicate DNA foci outside the mitochondria. Scale bars, 10μm. **g**, Quantification of DNA foci outside the mitochondrial network per cell. *n* = 3. **h**, Western blot showing the efficiency of BAX and BAK knockout in irradiation-induced senescent cells. **i**, mRNA expression of SASP genes in proliferative or irradiation-induced senescent Δ*BAX/BAK1* cells and controls. Data are expressed as fold change relative to proliferative cells. *n* = 3. **j**, Immunofluorescence of nuclear acetylated lysine. Scale bars, 30 μm. **k**, Quantification of nuclear acetylated lysine integrated density per cell. *n* = 3. **l**, Western blot showing no change in H3K27ac in irradiation-induced Δ*BAX/BAK1* cells compared with controls. **m**, Schematic representation of the experimental setup. Created in BioRender; Martini, H. https://biorender.com/of88ogz (2026). **n**, Western blot showing H3K27ac and the absence of mitochondria (UQCRC2) in mitochondria-depleted conditions. **o**, mRNA expression of SASP genes under the indicated conditions. Data are expressed as fold change relative to proliferative cells. *n* = 3. One-way ANOVA with Tukey’s multiple comparison test performed on groups with Sen (IR) + CCCP. **p**, Heat map of SASP factors in irradiation-induced senescent cells lacking mitochondria that were supplemented with acetate alone or acetate plus mtDNA. *z*-score representation. *n* = 3. **q**, GSEA enrichment plot of SASP factors. NES, normalized enrichment score. Data are mean ± s.e.m. Statistical tests: One-way ANOVA with Tukey’s multiple comparison test (**c**,**g**,**k**,**o**), uncorrected Fisher’s LSD (**i**), or two-tailed Student’s *t*-test (**d**). *n* represents the number of independent experiments. Source data

**Extended Data Fig. 9:**
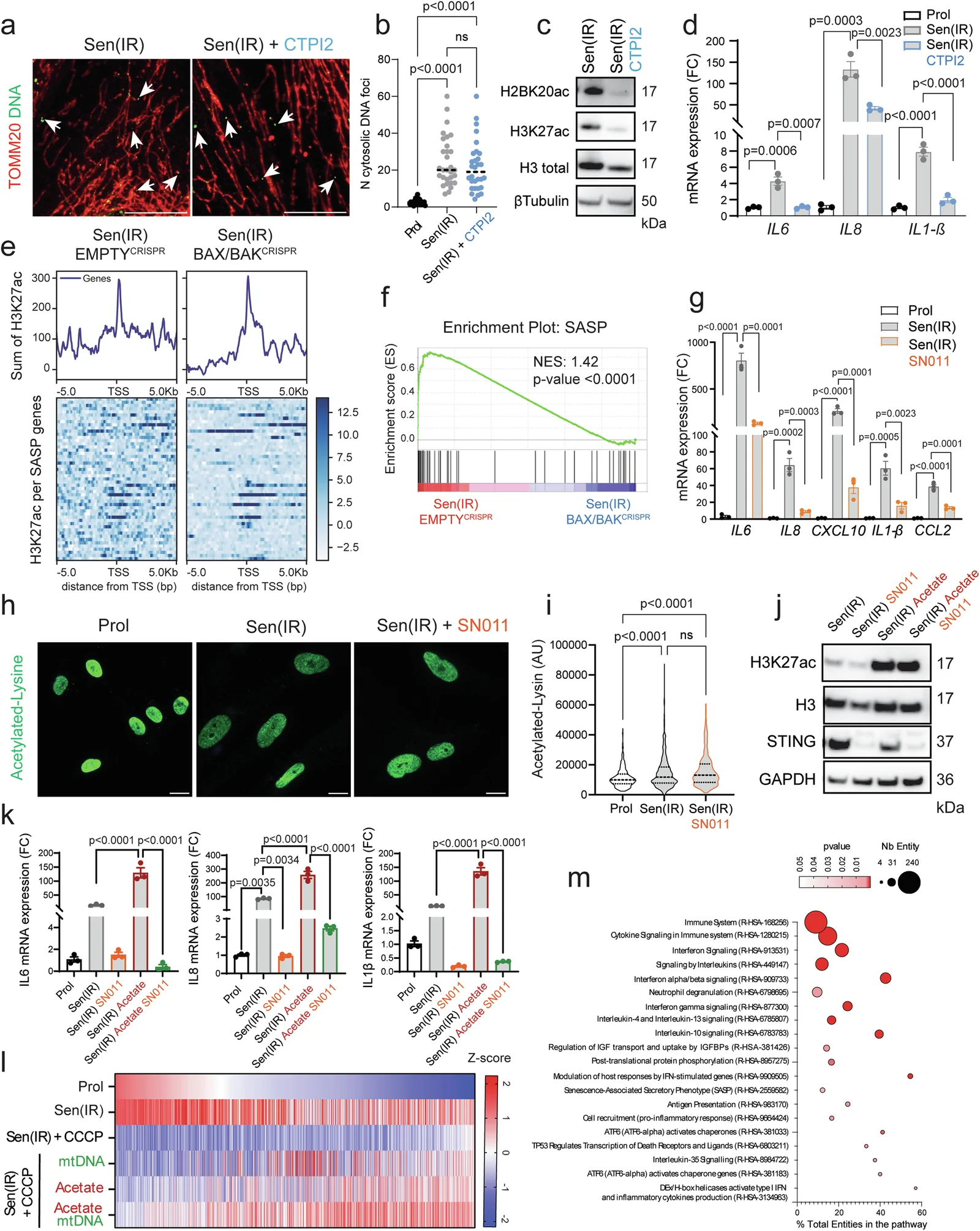
Mitochondrial citrate metabolism and mtDNA/cGAS/STING signaling independently regulate the SASP. (**a**) Immunofluorescence (IF) of the mitochondrial network (TOMM20) and DNA staining in senescent cells treated or not with CTPI2 (IMR90). Arrows show DNA foci outside the mitochondria network. Scale bar = 20 μm (**b**) Quantification DNA foci outside the mitochondria network per cell, from n = 3. (**c**) Western blot showing reduced histone acetylation after CTPI2 treatment in Sen(IR) IMR90. (**d**) mRNA expression of SASP genes in Prol or senescent (Sen(IR)) IMR90 treated or not with CTPI2. Fold change relative to Prol. (n = 3). (**e**) Metaplot and heatmap of H3K27ac at SASP genes in senescent cells lacking BAX/BAK, and control. Top: Metaplot of sum of normalized H3K27ac at SASP gene signals; Bottom: heatmap of H3K27ac enrichment normalized by total H3 (±5 kb from TSS). Averaged from n = 2. (**f**) GSEA enrichment plot for the SASP factors. (**g**) mRNA expression of SASP genes in Prol or Sen(IR) MCR5 treated or not by STING inhibitor SN011. Fold change relative Prol. (n = 3). (**h**) IF of nuclear acetylated lysine in Prol and Sen(IR) treated or not with SN011. Scale bar = 20 μm. (**i**) Quantification of nuclear acetylated-lysine per cell (n = 3). (**j**) WB showing increased histone acetylation (H3K27ac) by acetate treatment, not modifed after SN011 treatment. (**k**) mRNA expression of SASP genes in Prol or senescent (Sen(IR)) IMR90 treated or not by SN011 or acetate (n = 3). (**l**) Heatmap of all genes increasing by acetate and mtDNA transfection compared to senescent cells lacking mitochondria. Z-score representation from the average of n = 3. (**m**) Reactome pathways analysis of genes in (l). Data are shown as Mean ± s.e.m. Statistical tests: One-way ANOVA with Tukey’s multiple comparison test (b,d,g,I,k), weighted Kolmogorov Smirnov-like test (f) and Hypergeometric test with Benjamini–Hochberg FDR correction (m). (n = independent experiment). Source data

To determine whether suppression of SASP through inhibition of mitochondrial-dependent histone acetylation involves altered mtDNA signalling, we quantified cytosolic mtDNA following SLC25A1 inhibition (Fig. 5a). Cytosolic mtDNA levels were unchanged in senescent MRC5 and IMR90 fibroblasts following SLC25A1 inhibition (Fig. 5b–d and Extended Data Fig. 9a–d), indicating that metabolic regulation of histone acetylation restrains SASP independently of mtDNA release.

We next tested the reciprocal relationship. Senescent BAX- and BAK1-deficient fibroblasts, which exhibit impaired cytosolic mtDNA release, showed reduced SASP gene expression (Fig. 5e–i and Extended Data Fig. 9f), as previously reported^11^. However, global nuclear acetylation and H3K27ac levels, as well as H3K27ac enrichment at SASP loci, were unchanged (Fig. 5j–l and Extended Data Fig. 9e). Similarly, pharmacological inhibition of cGAS–STING signalling using the STING inhibitor SN011 suppressed SASP gene expression without affecting histone acetylation (Extended Data Fig. 9g–i). Together, these findings indicate that mtDNA–cGAS–STING signalling and mitochondrial control of histone acetylation operate as independently regulated pathways.

Despite this independence, we next explored whether these pathways functionally cooperate to drive SASP expression. Acetate supplementation increased H3K27ac levels in senescent cells independently of STING inhibition (Extended Data Fig. 9j), but acetate-induced SASP gene expression was suppressed by SN011 (Extended Data Fig. 9k), indicating that chromatin accessibility alone is not sufficient to drive SASP transcription in the absence of innate immune activation.

We therefore tested whether combined activation of these pathways is sufficient to reconstitute SASP expression. Senescent cells lacking mitochondria were treated with acetate, mtDNA, or both (Fig. 5m). mtDNA transfection did not alter H3K27ac levels, whereas acetate increased histone acetylation, with no additional effect of mtDNA co-treatment (Fig. 5n), confirming that mtDNA signalling does not directly regulate histone acetylation. Transcriptomic and pathway analyses revealed enrichment of inflammatory and cytokine signalling programmes following either treatment alone, with markedly greater enrichment when both were combined (Extended Data Fig. 9l,m). Consistently, qPCR and RNA-seq analyses showed that acetate or mtDNA alone partially induced SASP gene expression, whereas their combination resulted in a stronger activation of the SASP programme (Fig. 5o–q).

Together, these findings demonstrate that mitochondrial metabolic control of histone acetylation and mtDNA–cGAS–STING signalling represent distinct but complementary pathways that converge to enable full SASP gene expression.

## SLC25A1 inhibition improves healthspan

Given that mitochondrial metabolism regulates SASP gene expression through acetyl-CoA-dependent chromatin remodelling, we next tested whether this pathway can be targeted in vivo to suppress inflammation and improve tissue function during ageing. As an initial step, we assessed the effects of SLC25A1 inhibition ex vivo using stromal cells isolated from young and aged mouse tissues. Aged stromal cells from heart and kidney exhibited increased expression of p16 and genes encoding SASP factors compared with young controls, which were selectively reduced by treatment with the SLC25A1 inhibitor CTPI2 without altering p16 expression (Supplementary Fig. 1a–f). These findings suggested that targeting mitochondrial citrate metabolism can attenuate SASP in aged cells, prompting us to test this approach in vivo.

**Fig. 6:**
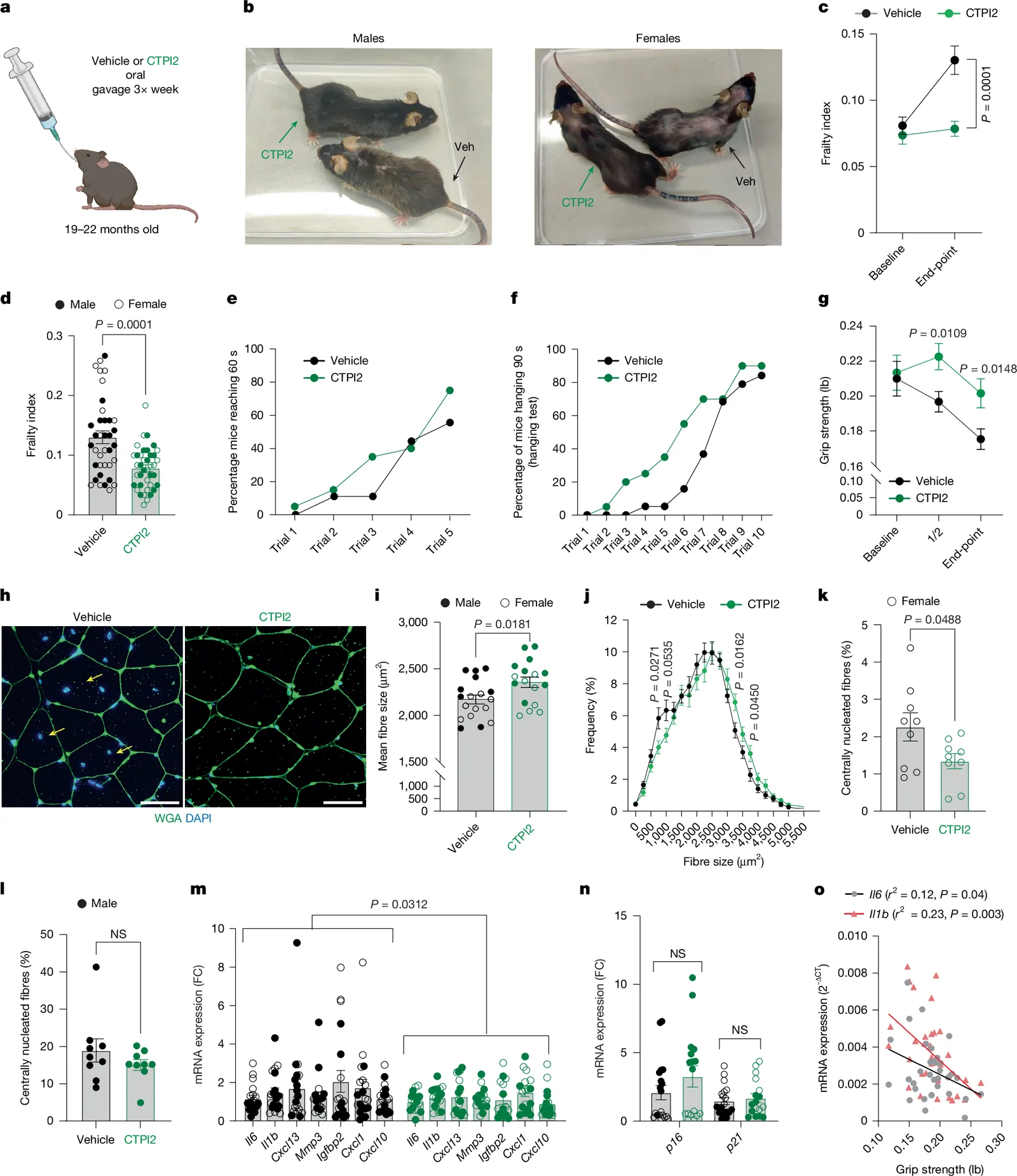
Pharmacological inhibition of SLC25A1 during ageing improves frailty and muscle function. **a**, Schematic representation of the in vivo experimental procedure. Created in BioRender; Passos, J. https://biorender.com/zjxy02g (2026). **b**, Representative images of male and female mice treated with either vehicle or CTPI2. **c**, Frailty index scores at baseline (0 months) and after 3 months of treatment (end-point) in vehicle-treated (male, *n* = 18; female, *n* = 19) and CTPI2-treated (male, *n* = 19; female, *n* = 20) mice. **d**, Histogram of frailty index scores after 3 months of treatment. Vehicle: male, *n* = 18; female, *n* = 19. CTPI2: male, *n* = 19; female, *n* = 20. **e**, Cumulative percentage of mice that passed the 60-s tightrope test. *n* = 18 vehicle and *n* = 20 CTPI2-treated mice; male and females were combined. **f**, Cumulative percentage of mice that passed the 90-s hanging test. *n* = 19 vehicle, *n* = 20 CTPI2-treated mice; male and female mice were combined. **g**, Forelimb grip strength was measured at baseline, 1.5 months and 3 months (end-point). Vehicle: male, *n* = 10; female, *n* = 9. CTPI2: male, *n* = 9; female, *n* = 10. **h**, Wheat germ agglutinin (WGA) staining, marking the membrane of cross-sectional myofibres. Scale bars, 50 μm. **i**, Quantification of mean cross-sectional myofibre area per mouse. Vehicle: male, *n* = 9; female, *n* = 9. CTPI2: male, *n* = 9; female, *n* = 8. **j**, Distribution of cross-sectional myofibre area per mouse. Number of mice as in **i**. **k**,**l**, Percentage of centrally nucleated fibres per mouse in female (**k**; *n* = 9 per condition) and male (**l**; *n* = 9 per condition) mice. **m**,**n**, mRNA expression of SASP genes (**m**) and cell cycle inhibitors (**n**) in quadriceps muscle, expressed as fold change relative to vehicle. Vehicle: male, *n* = 10; female, *n* = 9. CTPI2: male, *n* = 9; female, *n* = 8. **o**, Correlation curves of *Il1b* or *Il6* expression with forelimb grip strength for each mouse. Vehicle: male, *n* = 10; female, *n* = 9. CTPI2: male, *n* = 9; female, *n* = 8). Data are mean ± s.e.m. Two-tailed Student’s *t*-test. Males are represented by filled dots and females are represented by open dots. *n* represents the number of mice. Source data

We therefore administered CTPI2 to aged mice and assessed its effect on tissue function and systemic inflammation (Fig. 6a). CTPI2-treated mice (both male and female) exhibited a more lustrous and uniform fur coat, reduced alopecia and less fur greying (Fig. 6b), accompanied by delayed onset of frailty as measured by a 31-parameter frailty index^22^ (Fig. 6c,d).

Functional assessment revealed enhanced neuromuscular performance, including improved coordination and balance (tightrope test), increased strength (hanging and grip strength tests) and preservation of muscle integrity, as indicated by increased myofibre cross-sectional area and reduced centrally nucleated fibres (Fig. 6e–l). Despite these improvements in musculoskeletal phenotypes, CTPI2 treatment did not improve spine and femur bone microarchitecture (Supplementary Table 1).

**Extended Data Fig. 10:**
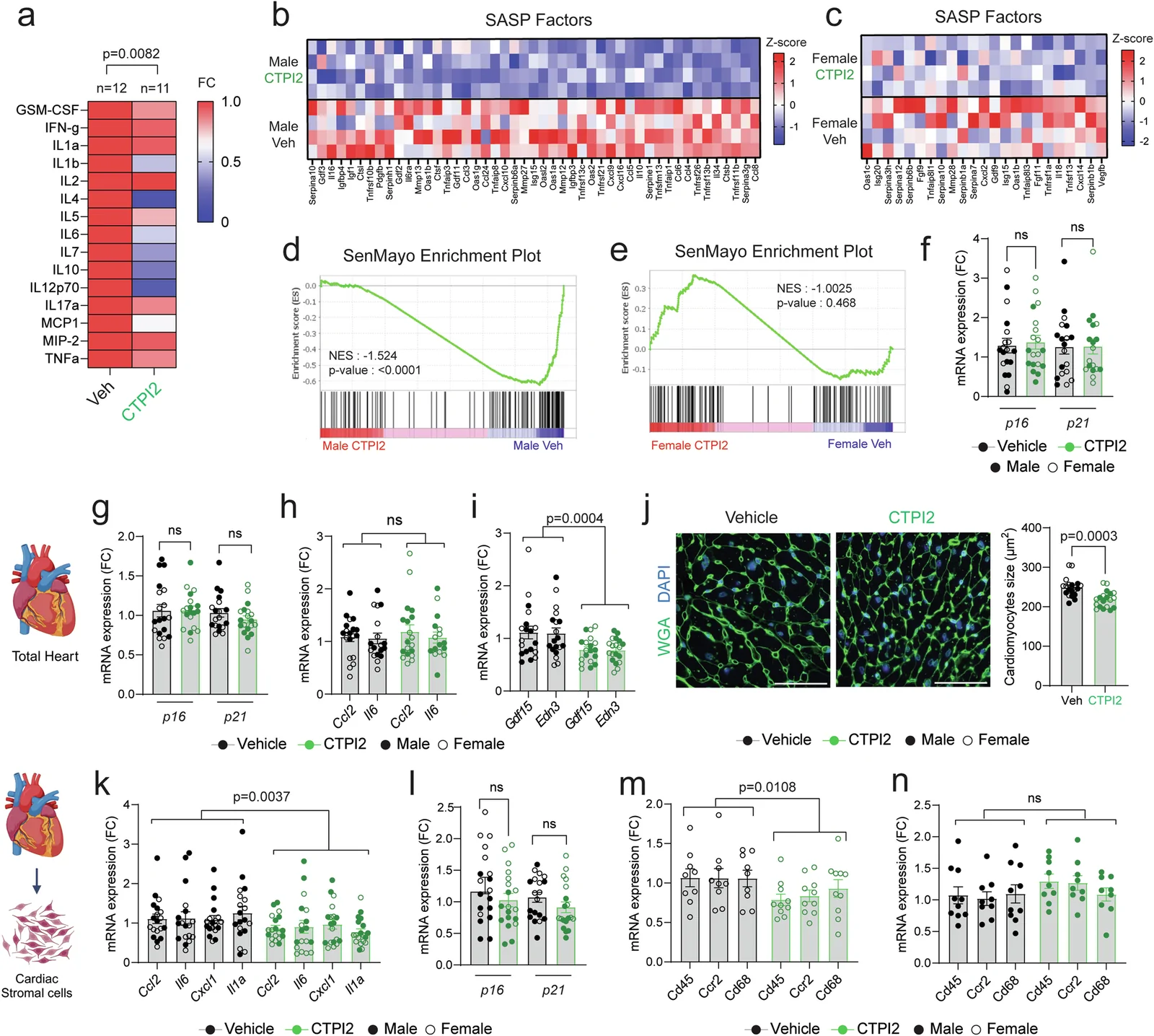
Inhibition of the mitochondrial citrate carrier SLC25A1 attenuates age-associated SASP and cardiac hypertrophy. (**a**) Heatmap of plasma cytokine levels from vehicle- or CTPI2–treated mice. Data are expressed as fold change compared to vehicle-treated animals (n = 12 vehicle, n = 11 CTPI2, male and female combined). (**b-c**) Clustered heatmap of SASP gene expression in mouse liver showing lower SASP expression in CTPI2–treated males (b) and females (c) (RNA-sequencing). Color intensity represents column Z-score (n = 4 mice per condition). (**d-e**) GSEA plots of SenMayo genes for CTPI2–treated versus vehicle males (d) or females (e). (**f**) Relative mRNA expression of cell cycle inhibitors p16 and p21 in total liver tissue from male and female mice, expressed as fold change compared to vehicle-treated. (**g-i**) Relative mRNA expression of cell cycle inhibitors *p16* and *p21* (g), classical SASP components (h), and cardiomyocyte-associated SASP factors (i) in total heart tissue, expressed as fold change compared to vehicle-treated mice. (**j**) Left: Representative immunofluorescence images of WGA staining marking cardiomyocyte membranes, scale bar = 50 μm. Right: Quantification of mean cross-sectional cardiomyocyte area per field and per mouse. (**k-l**) mRNA expression of SASP components (k) and cell cycle inhibitors *p16* and *p21* (l) in ex vivo cardiac stromal cells from male and female mice, expressed as fold change compared to vehicle-treated. Drawings in **g**,**k** created in BioRender; Martini, H. https://biorender.com/ttn9alp (2026). (**m-n**) Relative mRNA expression of pro-inflammatory macrophage markers in ex vivo cardiac stromal cells from female (m) and male (n) mice, expressed as fold change compared to vehicle-treated mice. Data are presented as mean ± s.e.m. Statistical analysis was performed using Two-tailed Student’s t-test (f,g,j,l) or Two-tailed Nested Student’s t-test (g,I,k,m,n). Vehicle: n = 10 males and 9 females; CTPI2: n = 9 males and 10 females (f-n). Males are represented by filled dots and females by open dots. (n = mice). Source data

Consistent with suppression of inflammatory signalling, CTPI2 treatment reduced SASP gene expression in skeletal muscle without altering expression of p16 or p21 (Fig. 6m,n). Expression of pro-inflammatory cytokines *Il6* and *Il1b* negatively correlated with muscle strength (Fig. 6o), suggesting that reduced inflammation contributes to improved tissue function. Circulating inflammatory cytokines were also reduced (Extended Data Fig. 10a), indicating a systemic attenuation of inflammatory burden.

To determine whether these effects extend to other organs, we performed RNA-seq of liver tissue. CTPI2 reduced expression of SASP-associated genes and decreased enrichment of the SenMayo senescence signature (Extended Data Fig. 10b–e), with more pronounced effects in male mice, whereas expression of p16 and p21 remained unchanged (Extended Data Fig. 10f).

In the heart, p16 and p21 expression was similarly unchanged (Extended Data Fig. 10h). However, CTPI2 specifically reduced cardiomyocyte-associated secretory factors (*Gdf15* and *Edn3*)^23^ in the whole heart, consistent with reduced cardiomyocyte hypertrophy. This occurred alongside with suppression of classical SASP factors in cardiac stromal cells (Extended Data Fig. 10g–l). These changes were further associated with reduced expression of pro-inflammatory CCR2^+^ macrophages markers in female mice (Extended Data Fig. 10m,n), consistent with decreased SASP-driven immune recruitment previously described in cardiac ageing^24^.

Together, these findings demonstrate that targeting mitochondrial citrate metabolism suppresses inflammation across tissues and improves tissue function and healthspan during ageing, without detectable changes in p16 or p21 expression, consistent with SASP modulation rather than senescent cell clearance.

## SLC25A1 inhibition remodels SASP chromatin

**Fig. 7:**
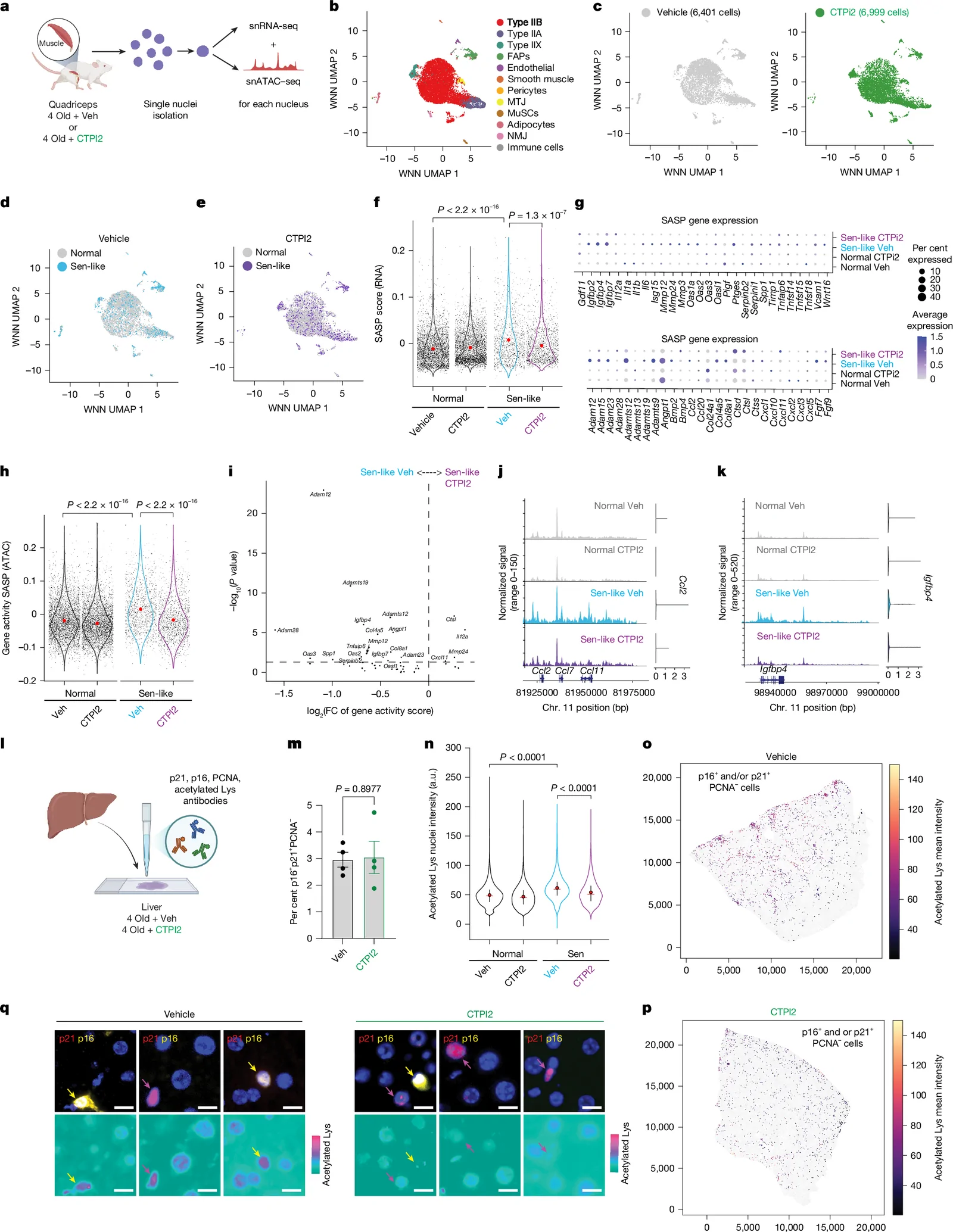
Pharmacological inhibition of SLC25A1 suppresses SASP programmes and chromatin accessibility in senescent cells in vivo. **a**, Schematic of the multiome experiment workflow. Created in BioRender; Martini, H. https://biorender.com/34h0t62 (2026). **b**, Weighted nearest neighbour (WNN) uniform manifold approximation and projection (UMAP) showing cell-type clusters. FAPs, fibro-adipogenic progenitors; MTJ, myotendinous junction; MuSCs: muscle stem cells; NMJ: neuromuscular junction. **c**, WNN UMAP illustrating the number and distribution of cells from vehicle-treated and CTPI2-treated samples. **d**,**e**, WNN UMAP showing the distribution of senescent-like cells across clusters in vehicle-treated (**d**) and CTPI2-treated (**e**) samples. **f**, Violin plot of per-nucleus SASP score based on RNA expression. **g**, Dot plot showing mean SASP gene expression and the proportion of cells expressing each SASP factor across the four conditions. **h**, Violin plot of per-nucleus gene activity scores for SASP factors derived from assay for transposase-accessible chromatin with sequencing (ATAC–seq). **i**, Volcano plot comparing gene activity scores of individual SASP genes in senescent-like cells from vehicle-treated versus CTPI2-treated samples. **j**,**k**, Coverage plots for the representative SASP genes *Ccl2* (**j**) and *Igfbp4* (**k**). **l**, Schematic of the experimental workflow for histological analyses. Created in BioRender; Martini, H. https://biorender.com/34h0t62 (2026). **m**, Percentage of cells classified as p16^+^ and/or p21^+^ and PCNA^−^ (non-proliferative) per sample. **n**, Nuclear acetylated lysine intensity in p16^+^p21^+^PCNA^−^ (senescent) and normal (proliferative) cells in vehicle-treated and CTPI2-treated groups. **o**,**p**, Representative spatial plots of liver sections from vehicle-treated (**o**) and CTPI2-treated (**p**) mice, showing the distribution of p16^+^p21^+^PCNA^−^ cells and their nuclear acetylated lysine intensity. **q**, Representative immunofluorescence images of p21 and p16 and acetyl lysine staining in vehicle-treated and CTPI2-treated livers from *n* = 4 mice per condition (one time staining). Scale bars, 35 μm. Data in **f**,**h**,**n** are shown as mean values (red dots); data in **m** are mean ± s.e.m. Two-tailed Wilcoxon test (**f**,**h**); two-tailed Wilcoxon rank sum test (**i**); one-way ANOVA with Bonferroni’s multiple comparison test (**n**); or two-tailed Student’s *t*-test (**m**). Source data

Given that SLC25A1 inhibition attenuates SASP through reduced cytosolic acetyl-CoA and histone acetylation in vitro, we next tested whether similar epigenetic mechanisms operate in vivo. Because SLC25A1 inhibition improves muscle function in aged mice, we focused on quadriceps muscle as a physiologically relevant tissue. To address this, we performed single-nucleus multiome profiling of quadriceps muscle from aged mice treated with the SLC25A1 inhibitor CTPI2, enabling simultaneous assessment of transcriptional output and chromatin accessibility within the same nucleus (Fig. 7a).

**Extended Data Fig. 11:**
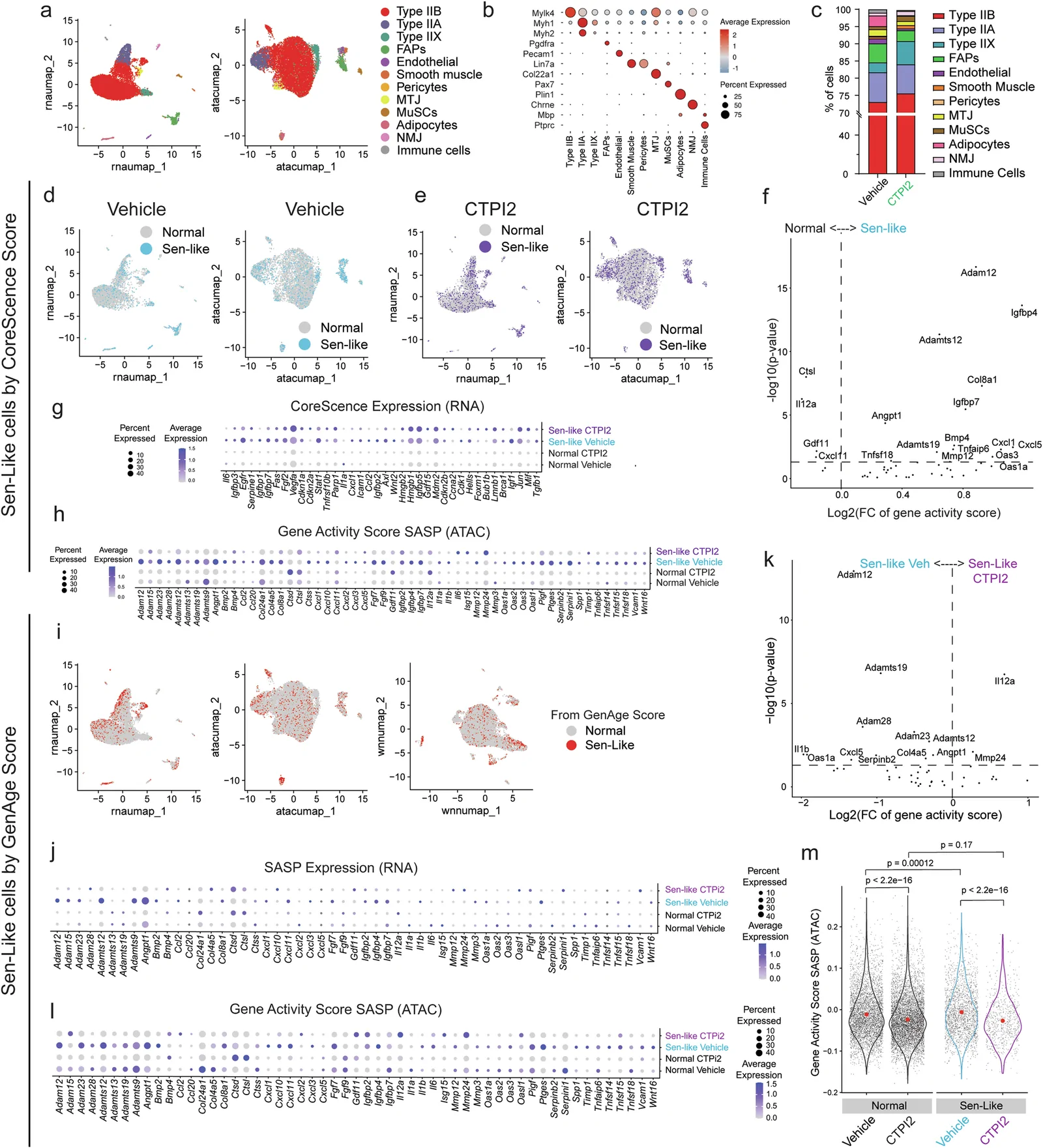
Pharmacological inhibition of SLC25A1 suppresses SASP programs and chromatin accessibility in senescent cells in vivo. (**a**) UMAPs representing the different cell type clusters from sn-RNA data set (left panel) or sn-ATAC-seq (right panel) (**b**) Dot plot showing the experession of the differents markers used for cell type identification (**c**) Histogram showing the cell type repartition between Vehicle or CTPI2 sample. (**d-e**) UMAPs showing the repartition of Sen-like cells in Vehicle (**d**) or CTPI2 (**e**) samples from sn-RNA data set (left panel) or sn-ATAC-seq (right panel). (**f**) Volcano plot of gene activity of the SASP genes in normal vs Sen-like cells in the vehicle group. (**g**) Dot plot of average expression and abundance of the different CoreScence genes between normal and Sen-like cells in the samples treated or not by CTPI2. (**h**) Dot plot of average gene activity and abundance of different SASP genes. (**i**) UMAPs abundance and repartition of senescent cells likes identified by high GeneAge Score. (**j-l**) Dot plot of average and abundance of gene expression (**j**) or gene activity (**l**) of SASP genes in Sen-like cells identified by GeneAge Score. (**k**) Volcano plot of gene activity of the SASP genes in Sen-like cells vehicle vs CTPI2 group identified by GeneAge score. (**m**) Violin plot of individual nuclei gene activity of SASP genes. Data are represented as Mean (red dots). Statistical analysis was performed using Two-tailed Wilcoxon Rank Sum test (f, k) and Two-tailed Wilcoxon test (m). Source data

Cell-type annotation revealed a predominance of type IIA and IIB myofibres, with comparable cellular composition between vehicle- and CTPI2-treated groups (Fig. 7b,c and Extended Data Fig. 11a–c). Immune cell abundance was reduced in CTPI2-treated muscles, consistent with diminished inflammatory burden (Extended Data Fig. 11c). Senescent-like nuclei, defined using a validated CoreScence gene signature^25^, were present at similar abundance and distribution across conditions (Fig. 7d,e and Extended Data Fig. 11d,e,g), consistent with unchanged expression of p16 and p21 (Fig. 6n).

Despite unchanged senescent cell abundance, CTPI2 treatment significantly reduced SASP-associated transcription within senescent-like nuclei (Fig. 7f,g). This transcriptional suppression was accompanied by decreased chromatin accessibility at SASP loci, which were selectively enriched in senescent-like cells and attenuated by CTPI2 treatment (Fig. 7h,i and Extended Data Fig. 11f,h). Representative loci, including *Ccl2* and *Igfbp4*, showed increased accessibility in senescent cells that was reduced upon treatment (Fig. 7j,k). Similar results were obtained using an independent senescence gene set^26^ (Extended Data Fig. 11i–m).

To further assess histone acetylation in vivo, we analysed the liver, an organ in which CTPI2 treatment markedly reduced inflammation. Iterative indirect immunofluorescence imaging (4i) was performed using antibodies against p21, p16, PCNA and pan-lysine acetylation (Fig. 7l). Given concerns regarding p16 antibody specificity, the signal was validated using p16-knockout mice treated with the senescence-inducing agent doxorubicin (Supplementary Fig. 2). Across treatment conditions, the abundance of p16^+^ and/or p21^+^PCNA^−^ cells remained unchanged (Fig. 7m), consistent with transcriptional data. By contrast, these cells exhibited increased nuclear histone acetylation compared to non-senescent cells, which was reduced following CTPI2 treatment (Fig. 7n–q).

**Extended Data Fig. 12:**
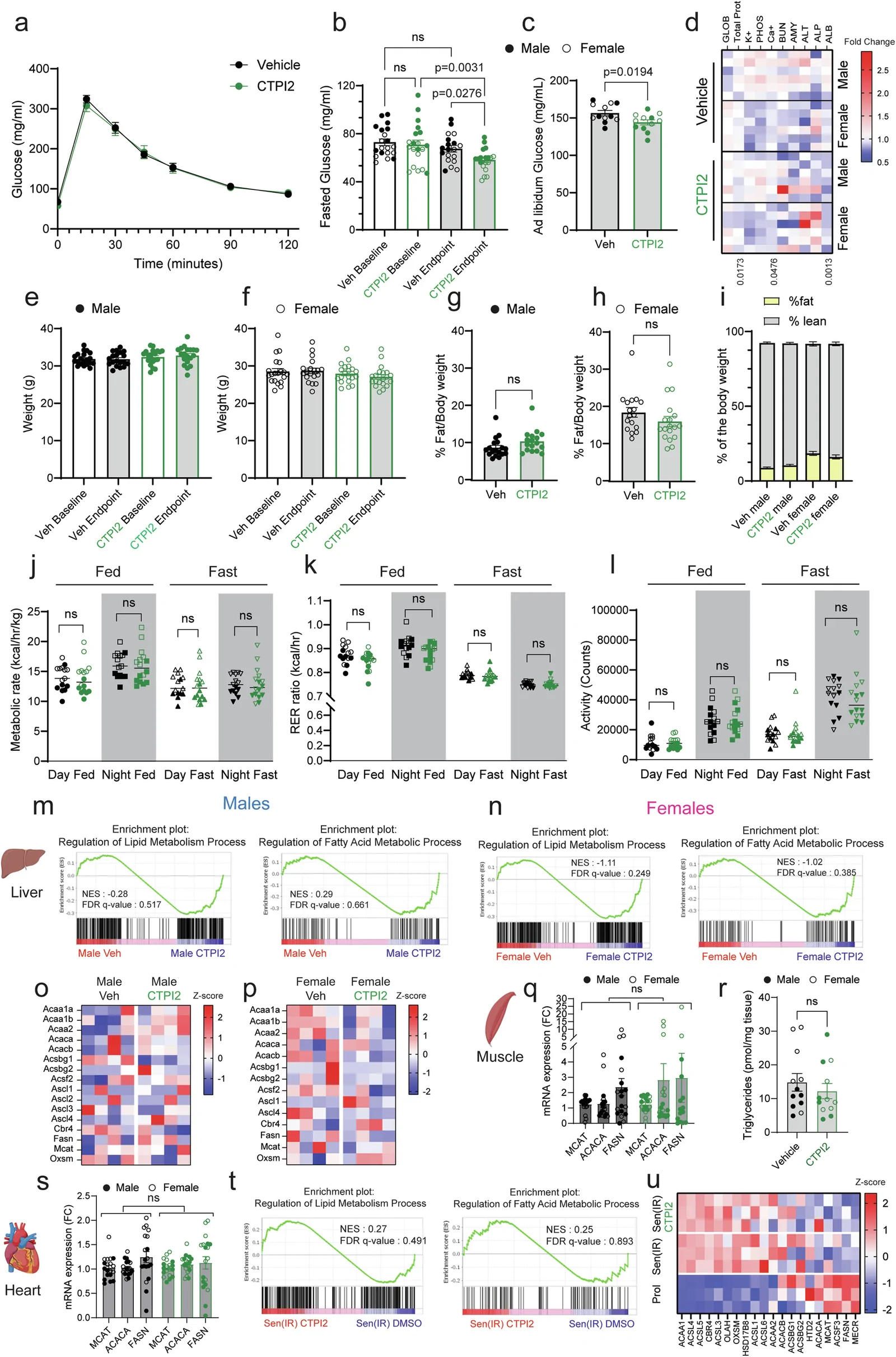
Pharmacological inhibition of the mitochondrial citrate carrier (SLC25A1) does not induce systemic metabolic reprogramming in mice. (**a**) Intraperitoneal glucose tolerance test (IP-GTT) (Vehicle n = 19; CTPI2 n = 20; males and females combined). (**b**) Fasting blood glucose levels at baseline and after 3 months treatment (Endpoint) (Vehicle n = 19; CTPI2 n = 20; males and females combined). (**c**) Ad libitum blood glucose levels (n = 6 per sex). (**d**) Plasma chemistry parameters (n = 6 per sex). (**e–f**) Body weight of male (e) and female (f) mice at baseline and endpoint (n = 20 at baseline per sex, and n = 19 at endpoint for all except Female CTPI2 endpoint n = 20). (**g-h**) Fat mass normalized to body weight in male (g) (Vehicle n = 18, CTPI2 n = 17) and female (h) (Vehicle n = 17, CTPI2 n = 18) mice. (**i**) Whole-body composition normalized to body weight. (**j-l**) Comprehensive Lab Animal Monitoring System (CLAMS) measurements, including metabolic rate (j), respiratory exchange ratio (RER) (k), and locomotor activity (l), across light/dark cycles under fed and fasting conditions (n = 8 per sex). (**m-n**) GSEA plots comparing liver transcriptomes of Vehicle- and CTPI2-treated males (m) and females (n). (**o-p**) RNA-seq heatmaps of key lipid metabolism genes in male (o) and female (p) livers (Z-score representation; n = 4). (**q**) Relative mRNA expression of key lipid metabolism enzymes in quadriceps. (Vehicle: male n = 10, female n = 9; CTPI2: male n = 9, female n = 8) (**r**) Triglyceride content in quadriceps (n = 6 per sex). (**s**) Relative mRNA expression of lipid metabolism enzymes in heart tissue (Vehicle: male n = 10, female n = 9; CTPI2: male n = 9, female n = 10). (**t**) GSEA plot comparing senescent cells ± CTPI2. (**u**) RNA-seq heatmap of lipid metabolism genes in Prol and senescent cells ± CTPI2 (Z-score representation; n = 3). Data are presented as mean ± s.e.m. Statistical analyses were performed using one-way ANOVA with Tukey’s multiple comparison test (b) or Two-tailed Student’s *t*-test. Males are represented by filled dots and females by open dots. (n = mice). Drawings in **m**,**s** created in BioRender; Martini, H. https://biorender.com/ttn9alp (2026). Drawing in **q** created in BioRender. Martini, H. (2026) https://biorender.com/ssht0mx (2026). Source data

To determine whether these effects are accompanied by broader metabolic alterations, we performed comprehensive metabolic profiling. CTPI2-treated aged mice displayed glucose tolerance comparable to controls, with modest reductions in fasting and fed glucose levels (Extended Data Fig. 12a–c). Other circulating biochemical parameters, including total protein, calcium and albumin, were largely unchanged and remained within physiological ranges (Extended Data Fig. 12d). Body weight, body composition, metabolic rate, respiratory exchange ratio and activity were also unaffected (Extended Data Fig. 12e–l). Lipid and fatty acid metabolic pathways in liver, muscle and heart were similarly unchanged, consistent with in vitro observations showing no effect of CTPI2 on lipid metabolic gene expression in senescent fibroblasts (Extended Data Fig. 12m–u).

**Extended Data Fig. 13:**
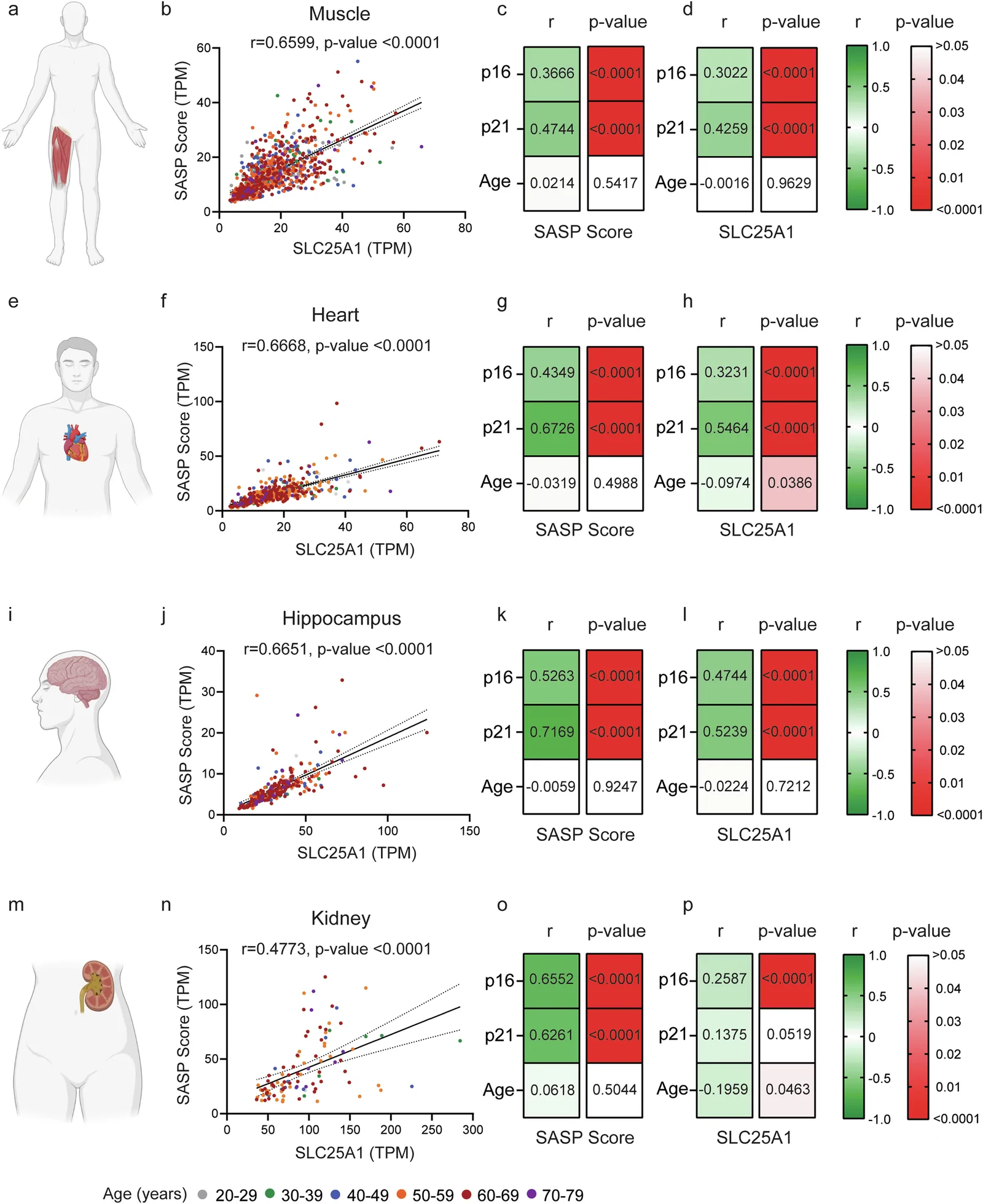
SLC25A1 expression correlates with SASP and senescence markers in human tissues. Schematic illustrations of the analyzed organs: skeletal muscle (**a**), heart (**e**), hippocampus (**i**), and kidney (**m**). Correlation analysis between *SLC25A1* expression and mitochondrial dependent SASP score in each human tissue (**b, f, j, n**). Two-tailed Pearson correlation coefficients and associated p values between SASP score and p16, p21, and age in each tissue (**c, g, k, o**). Two-tailed Pearson correlation coefficients and associated p values between *SLC25A1* expression and *p16*, *p21*, and age in each tissue (**d, h, l, p**). Sample sizes: muscle (n = 818), heart (n = 452), hippocampus (n = 255), kidney (n = 104). Drawings in **a**,**e**,**i**,**m** created in BioRender; Martini, H. https://biorender.com/gf8pzeh (2026). Source data

Finally, to assess the relevance of our findings in humans, we analysed transcriptomic datasets from multiple human tissues. Across muscle, heart, hippocampus and kidney, expression of the mitochondrial citrate transporter gene *SLC25A1* positively correlated with a mitochondrial-dependent SASP gene signature and with canonical senescence markers p16 and p21, whereas chronological age alone showed little or no association (Extended Data Fig. 13a–p). These observations suggest that mitochondrial citrate metabolism is linked to senescence-associated inflammatory programmes across diverse human tissues.

Together, these data indicate that CTPI2 treatment in aged mice suppresses SASP-associated transcription through selective epigenetic remodelling at inflammatory loci, without broadly altering systemic metabolism or senescent cell abundance. This epigenetic suppression of SASP provides a mechanistic basis for the observed improvements in tissue function and healthspan during ageing.

## Discussion

In this study, we identify mitochondrial metabolism as a regulator of histone acetylation and inflammatory gene expression during senescence. Using genetic and pharmacological perturbations across multiple nodes of the mitochondrial citrate–acetyl-CoA axis, including MPC, SLC25A1 and ACLY, we show that mitochondrial metabolism is required to sustain histone acetylation at SASP loci and maintain inflammatory gene expression. These findings position mitochondrial metabolism as an upstream determinant of chromatin accessibility in senescent cells. This aligns with previous reports linking citrate metabolism to SASP regulation^27^.

Although acetyl-CoA can be generated from alternative sources, including acetate, fatty acid oxidation and amino acid metabolism, our data indicate that in senescent cells, mitochondria constitute a dominant source of acetyl-CoA for chromatin modification. This suggests that the metabolic wiring of senescent cells creates a dependence on mitochondrial acetyl-CoA to sustain epigenetic activation of inflammatory gene programmes.

An important feature of our findings is the modulation of SASP, rather than the cell cycle arrest component of senescence. One explanation for this specificity lies in the chromatin architecture of SASP genes. Previous work has shown that many SASP-associated genes are linked to super-enhancer-like regions that are particularly sensitive to changes in histone acetylation^28^. Such loci probably require sustained acetyl-CoA availability to maintain high levels of transcriptional output. Our data support a model in which SASP loci are highly responsive to fluctuations in acetyl-CoA levels, rendering them especially vulnerable to interventions that limit acetyl-CoA supply.

An important question raised by our work is whether mitochondrial metabolic regulation and mtDNA-driven innate immune signalling^3,12,13^ act through a single linear pathway to control SASP, or instead function as separate but cooperative inputs. Our data support the latter model, demonstrating that mitochondrial control of histone acetylation and mtDNA–cGAS–STING signalling function as independent pathways that converge to enable SASP gene expression. While mitochondrial acetyl-CoA availability allows chromatin accessibility at SASP loci, mtDNA-dependent innate immune signalling provides the inflammatory activation required to engage these transcriptionally permissive regions. Disruption of either pathway is sufficient to suppress SASP, yet activation of either alone is not sufficient to fully restore SASP expression, indicating that both signals are required.

CTPI2, a selective inhibitor of SLC25A1 (ref. ^20^), has previously been shown to improve metabolic dysfunction in diet-induced steatohepatitis^29^, a context associated with obesity and altered systemic metabolism, yet to our knowledge, its effects on ageing-associated phenotypes had not been explored prior to this study. Here we find that CTPI2 improves functional outcomes in aged mice while broadly suppressing SASP expression across tissues, without evidence of large-scale metabolic remodelling or reduced senescent cell abundance. This response differs from obesity-associated states, in which SLC25A1 inhibition influences glucose and lipid metabolism^29^, consistent with chronic nutrient excess and increased anabolic demand. Although both ageing and obesity involve metabolic alterations, our findings suggest that mitochondrial citrate export serves distinct roles in these settings. In aged tissues, citrate export appears to be less critical for sustaining bulk metabolic flux and instead contributes to acetyl-CoA-dependent chromatin acetylation and inflammatory gene expression. Accordingly, SLC25A1 inhibition selectively attenuates SASP-associated transcriptional programmes while preserving core metabolic functions.

These findings position SLC25A1 inhibition as a novel therapeutic target that modulates the inflammatory output of senescent cells through metabolic–epigenetic coupling. More broadly, they suggest that targeting metabolic inputs into chromatin regulation may represent a tractable strategy to mitigate age-associated inflammation and functional decline.

## Methods

### Cell culture and treatments

Human embryonic lung MRC5 fibroblasts (ATCC) and IMR90 fibroblasts (ATCC) were cultured in Dulbecco’s modified Eagle’s medium (Sigma Aldrich, D5796) supplemented with 10% heat-inactivated fetal bovine serum (FBS), 100 U ml^−1^ penicillin, 100 μg ml^−1^ streptomycin and 2 mM l-glutamine. The cultures were maintained at 37 °C in an atmosphere of 5% CO_2_. MRC5 fibroblasts were grown under atmospheric oxygen conditions, and IMR90 fibroblasts were cultured under low-oxygen (3%) conditions. Cells were tested regularly for mycoplasma contamination.

For lentiviral transduction HEK293T cells (ATCC) were used, cultured in DMEM without antibiotic and supplemented with 10% heat-inactivated fetal bovine serum (FBS) and 2 mM l-glutamine.

Stress-induced senescence was triggered by exposing cells to 20 Gy of X-ray irradiation and collected between 10 and 12 days post-irradiation. Chemotherapy-induced senescence was performed by treated cells with 250 nM of doxorubicin (MedChemExpress, HY-15142) for 24 h and collected 12 days after treatment. Replicative senescence was performed through serially passaging until the cells reached their replicative limit. Senescence was verified through the presence of p16 and p21, lack of proliferation and the expression of SASP genes.

For chronic acetate treatment, MRC5 or Parkin IMR90 cells were treated with 20 mM of sodium acetate solution (Sigma, S7899) for 10 to 12 days, with media refreshing every 48–72 h.

For chronic citrate treatment, MRC5 were supplemented with 10 mM of sodium citrate (Sigma, W302600) for 10 to 12 days, with media refreshing every 48–72 h.

For SLC25A1 and MPC pharmacological inhibition, MRC5 fibroblasts were irradiated with 20 Gy X-ray irradiation and treated with CTPI2 (Selleckchem, S2968) or UK5099 (Sigma, PZ0160) at the indicated concentrations (15 or 30 μM for CTPI2 and 100 μM for UK5099). CTPI2 and UK5099 were added one day after irradiation and maintained in the cell culture medium for 12 days (refreshed every 48–72 h). The same protocol was used for STING pharmacological inhibition where cells were treated with SN011 (Cayman Chemical, NC2044999) at concentration of 10 μM. For acetyl-CoA measurements, proliferative MRC5 were treated or not with 20 mM of sodium acetate-1-C13 (Sigma, 279293) or sodium acetate C12 (Sigma, S7899) and collected after 3 h. The analysis was performed in 4 million cells per condition.

### Parkin-mediated mitochondrial clearance

Parkin-mediated mitochondrial clearance was performed as previously described. In summary, proliferating or irradiated Parkin-overexpressing IMR90 fibroblasts were treated with 12.5 μM CCCP (Sigma Aldrich, C2759) one day post-irradiation (day 1) for a duration of 48 h, with CCCP being replenished every 24 h (day 1, day 2). Acetate was added at day 3 when mitochondria were cleared, and cells were collected at day 12 (media refreshed every 48–72 h).

### Lactate dehydrogenase cytotoxicity assay

Cytotoxicity was assessed using a lactate dehydrogenase (LDH) assay (Abcam, ab65939) following the manufacturer’s instructions. Cells were seeded in 24-well plates, irradiated on day 0, and mitochondrial clearance was induced on days 1 and 2 (day 1 and day 2) using CCCP. At day 11 post-irradiation, the culture medium was refreshed. On day 12, 50 µl of the medium was collected and mixed with 50 µl of LDH reaction mixture. Absorbance was measured at 450 nm using a plate reader.

### Subcellular fractionation and mtDNA extraction

Subcellular fragmentation and mtDNA extraction were performed following the previously described protocol^3^.

In summary, IMR90 were collected by trypsinization and washed once with PBS. Cells were then pelleted by centrifugation at 300*g* for 5 min at 4 °C. Cells were resuspended in mitochondrial isolation solution (MIS) (20 mM HEPES-KOH pH 7, 220 mM mannitol, 70 mM sucrose, 1 mM EDTA, 0.5 mM PMSF, 2 mM DTT) and transferred into a glass homogenizer on ice. Cells were broken open by strokes and efficiency was assessed under microscope with trypan blue staining. Cells were stroked until the homogenization was sufficient. The homogenate was then centrifuged twice at 800*g* for 5 min at 4 °C to remove all the cell debris and membranes. The supernatant was centrifuged at 16,100*g* for 10 min at 4 °C. The supernatant, which is the cytosolic fraction, was discarded and the pellet, which is the mitochondrial fraction, was washed once with MIS and centrifuged again at 16,100*g* for 10 min at 4 °C and the pellet was resuspended in 200 μl PBS.

DNA extraction was performed in the mitochondrial fraction using the DNeasy Blood & Tissue Kit (Qiagen, 69504) according to the manufacturer’s instructions.

### mtDNA transfection and acetate supplementation following Parkin-mediated mitochondrial clearance

Mitochondrial clearance was performed in Parkin-mediated cells one day after irradiation as previously described^3^. Cells were treated or not with 20 mM of sodium acetate solution (Sigma, S7899) every other day. On day 10 post-irradiation, cells were transfected with mtDNA with a concentration of 10 μg of mtDNA for 500,000 cells using DharmaFECT kb DNA transfection reagent (Horizon, T-2006-01), according to the manufacturer’s instructions.

### Measurement of mtDNA in the cytosolic fraction

Cytosolic fractionation was performed on 5 × 10^5^ cells per condition, and mtDNA levels were quantified using the Absolute Human Mitochondrial DNA Copy Number Quantification qPCR Assay Kit (ScienCell, 8948) according to the manufacturer’s instructions.

### CRISPR–Cas9-based genome editing

The following plasmids were used:

hSLC25a1 CRISPR (sgRNA #231; Vector-Builder, VB900058-0699rvd), hMPC1 CRISPR (5′-CACCGGGGCTACTTCATTTGTTGCG-3′ AND 5′-AAACCGCAACAAATGAAGTAGCCCC-3′), hBAK CRISPR (Addgene, 129579), hBAX CRISPR (Addgene, 129580), Puro CRISPR (Addgene, 52961) (Plenti control).

For lentiviral transduction, HEK293FT cells were transfected with the plasmids above together with the packaging and envelope plasmids VSVG and Gag-Pol (Sigma Aldrich) using Lipofectamine 3000 (Invitrogen, L3000015) according to the manufacturer’s instructions. Then, 2 days later, the supernatant from the transfected HEK293FT cells containing viral particles was filtered using a 0.45-μm pore PVDF filter, mixed with 10 μg ml^−1^ of polybrene and used to infect the cells of interest. After infection, cells were selected for successful CRISPR–Cas9 deletion using the following antibiotics: 1 μg ml^−1^ of puromycin.

### siRNA transfection

si*ACLY* (Sigma, SASI_Hs01_00239323) and a scrambled control siRNA (Sigma, SIC001) were used. Cells were transfected with siRNA at a final concentration of 30 nM using DharmaFECT 2 transfection reagent (Horizon, T-2002-03) at a ratio of 0.3 μl per 100 μl transfection medium, according to the manufacturer’s instructions.

#### Irradiation-induced senescence

Cells were first transfected in T75 flasks for 24 h, then exposed to 20 Gy X-ray irradiation the following day. Cells were reseeded into 6-well plates 1 day post-irradiation (day 1) and transfected a second time at day 8 post-irradiation for 24 h. Cells were collected at day 10 post-irradiation for analysis.

#### Replicative senescence

Replicatively senescent cells were transfected twice, at day 1 and day 4, each for 24 h using the same conditions, and collected at day 6 for analysis.

### ^13^C-glucose tracing of H3K9ac

MRC5 cells (3 × 10^6^) were exposed to 20 Gy X-ray irradiation and maintained under standard culture conditions for 11 days. Cells were then incubated for 24 h in either standard DMEM (Gibco, 11965-092) containing unlabelled glucose (4.5 g per 500 ml) or glucose-free DMEM (Gibco, 11966-025) supplemented with ^13^C-d-glucose (4.5 g per 500 ml; Cambridge Isotope Laboratories, CLM-1396-5). Both media were supplemented with 10% heat-inactivated fetal bovine serum, 100 U ml^−1^ penicillin, 100 μg ml^−1^ streptomycin and 2 mM l-glutamine.

After 24 h, cells were collected and histones were acid-extracted with 0.4 M HCl for peptide analysis. Histones were digested with sequencing-grade trypsin (Promega, V5113) at a 1:20 enzyme-to-substrate ratio in ammonium bicarbonate (Sigma Aldrich, A6141) at 37 °C for 6 h. Digestion was quenched by acidification to pH 4 with acetic acid and neutralized with ammonium hydroxide (Sigma Aldrich, 338818). Samples were concentrated to 100 μl under nitrogen gas and desalted using C18 ZipTip pipette tips (MilliporeSigma).

Peptides were analysed by liquid chromatography–mass spectrometry (LC–MS) using standard procedures. Peak apex intensities for H3K9ac peptides were obtained from extracted ion chromatograms at mass to charge ratio (*m*/*z*) 637 and 654. An internal acetyl-CoA standard was used for quantification. ^13^C enrichment was calculated using IsoPat2 (ref. ^30^), with unlabelled samples serving as natural-abundance controls.

### Cytosolic and mitochondrial fractionation for mass spectrometry

For subcellular fractionation, 4 × 10^6^ cells per sample were collected, washed with PBS and pelleted by centrifugation at 1,300 rpm for 5 min at 4 °C. Fractionation was performed as previously described^31^. Cell pellets were resuspended in KPBS buffer and homogenized using a Dounce homogenizer. Homogenization efficiency was assessed microscopically using trypan blue staining.

Homogenates were centrifuged at 1,000*g* for 10 min at 4 °C to remove nuclei and unlysed cells. The resulting supernatant was centrifuged at 17,000*g* for 10 min at 4 °C to separate mitochondria from the cytosolic fraction. The supernatant was collected as the cytosolic fraction. The pellet (mitochondrial fraction) was washed once with KPBS buffer and centrifuged again at 17,000*g* for 10 min. Aliquots of each fraction were reserved for western blot analysis to assess fraction purity prior to mass spectrometry.

### LC–MS analysis of acetyl-CoA in cells, mitochondria and cytosolic fractions

Mitochondria or cell pellets were resuspended in 200 μl of 2.5% 5-sulfosalicylic acid (SSA) spiked with 50 ng of acetyl-CoA-d_3_ standard (Cayman: 40458) per sample and vortexed for lysis. Fifty microlitres of cytosolic fraction was mixed with 200 μl of 2.5% SSA containing 50 ng of acetyl-CoA-*d*_3_ and vortexed. All the samples were centrifuged at 17,000*g* at 4 °C for 5 min, and the supernatants were transferred into glass auto-sampler vials. The samples were analysed with a 1260 Infinity II HPLC coupled to an Agilent 6150 single quadrupole LC–MS. Ten microlitres of each sample was injected onto an Agilent InfinityLab Poroshell 120 EC-C18 column (699675-742). Temperatures for the auto-sampler set at 4 °C and the column compartment at 40 °C, respectively. The mobile phase was composed of solvent A (50 mM formic acid in LC–MS-grade H2O, adjusted to pH 8.2 with ammonium hydroxide) and solvent B (100% LC–MS grade methanol). The chromatographic gradient was run at a flow rate of 0.3 ml min^−1^ as follows: 0–1 min: hold at 20% B; 1–11 min: linear gradient from 20% to 100% B; 11–12 min: hold at 100% B; 12–13 min: linear gradient from 100% to 0% B; 13–20 min: hold at 100% B. The mass spectrometer was operated in selected ion monitoring (SIM), positive ion mode with the capillary voltage set to 3.5 kV, the nozzle voltage set to 2 Kv. The sheath gas was held at 250 °C at the flow rate 10 l min^−1^ and the drying gas was held at 300 °C at the flow rate 5 l min^−1^. The nebulizer pressure was set to 20 psig. The retention time and position of the peaks were confirmed using pure appropriate standard compounds freshly prepared in LC–MS grade water. The peak area/height was quantified using Agilent ChemStation. *m*/*z* of the following ions were detected: acetyl-CoA (*m*/*z* 810.6–811.6) and acetyl-CoA-*d*_3_ (*m*/*z* 813.6). IsoPat2 software was used to adjust for natural abundance^30^.

### Citrate quantification by GC–MS for mitochondria and cytosolic fraction

Mitochondria pellets were resuspended in 500 μl 80% methanol spiked with 100 ng of ^13^C_6_ citrate standard and vortexed for lysis. Fifty microlitres of cytosolic fraction was mixed with 200 μl of 100% methanol containing 100 ng of ^13^C_6_ citrate and vortexed. All the samples were centrifuged at 17,000*g* at 4 °C for 5 min, and the supernatants were dried with nitrogen gas, dissolved in 75 μl dimethylformamide (DMF), then derivatized with 75 μl *N*-methyl-*N*-(*tert*-butyldimethylsilyl)trifluoroacetamide (MTBSTFA) + 1% tertbutyldimetheylchlorosilane (TBDMCS) (Regis). Samples were incubated at room temperature for 30 min and were analysed using an Agilent 7890B GC coupled to a 5977 A mass detector. Three microlitres of derivatized sample were injected into an Agilent HP-5ms Ultra Inert column, and the GC oven temperature increased at 15 °C min^−1^ up to 215 °C, followed by 5 °C min^−1^ up to 260 °C, and finally at 25 °C min^−1^ up to 325 °C. The mass spectrometer was operated in split-less mode with electron impact mode at 70 eV. Mass range of 50–700 was analysed, recorded at 1,562 mass units per second. Data were analysed by Agilent MassHunter Workstation Analysis and Agilent MSD ChemStation Data Analysis software to measure peak area/height of citrate for quantification. Citrate was detected by gas chromatography–mass spectrometry (GC–MS) as TBDMS derivatives at the following *m*/*z* values: citrate (*m*/*z* 591) and ^13^C_6_ citrate (*m*/*z* 597).

### TCA metabolites quantification

TCA cycle metabolites were quantified by GC–MS. Briefly, cell pellets were lysed in 50 μl PBS after addition of 20 μl internal standard solution containing uniformly ^13^C-labelled metabolites. Proteins were precipitated by adding 300 μl chilled methanol-acetonitrile, and samples were centrifuged to collect the supernatant. The supernatant was dried under vacuum and derivatized sequentially with ethoxime and MTBSTFA + 1% TBDMCS. Samples were analysed on an Agilent 5977B GC–MS (Agilent Technologies, Santa Clara, CA) under electron impact ionization in single-ion monitoring mode.

Metabolite concentrations were determined using 12-point calibration curves processed in parallel. The following ions (*m*/*z*) were monitored: lactate (261.2), fumarate (287.1), succinate (289.1), α-ketoglutarate (360.2), malate (419.3), aspartate (418.2), 2-hydroxyglutarate (433.2), *cis*-aconitate (459.3), citrate (591.4), isocitrate (591.4) and glutamate (432.4).

### Metabolomics by mass spectrometry

Metabolomic analyses following UK5099 treatment were performed. Cells were seeded in 6-well plates (5 × 10^5^−1.5 × 10^6^ cells per ml; triplicate wells per condition) in complete DMEM and allowed to adhere overnight for proliferating controls, or cultured and treated as previously described for Sen (IR) and Sen (IR) + UK5099 conditions. Cells were washed with PBS and incubated in the appropriate experimental medium for the indicated times.

Duplicate wells were used for cell counting, and counts were used to normalize extraction volumes (equivalent to 2 × 10^6^ cells per ml). Cells were rapidly washed with PBS, and ice-cold extraction solvent (methanol 50%, acetonitrile 30%, water 20%) was added before scraping on ice. Lysates were transferred to 1.5-ml tubes, vortexed, and centrifuged at 15,000 r.p.m. for 10 min at 4 °C. Supernatants were collected and stored at −80 °C until LC–MS analysis.

Chromatographic separation was performed using an isocratic program (80% solvent A, 20% solvent B) at a flow rate of 200 μl min^−1^ with a total run time of 3 min and an injection volume of 20 μl. Mass spectrometry was conducted on an Exactive instrument (Thermo Fisher Scientific) operating in positive ion mode, full-scan acquisition (*m*/*z* 50–800) at a resolution of 50,000.

### ChIP–seq

Chromatin immunoprecipitation was performed as described^32^. Cells were formalin-fixed for 10 min in 4% paraformaldehyde (PFA), then lysed and chromatin was sheared to between 250–500 bp as confirmed by agarose gel electrophoresis in size using sonication (Biorupter pico, B01060010). Protein G Dynabeads (Invitrogen, 1000D) were prepared with 5 μg of primary antibody of interest: histone H3 (Abcam ab1791), histone H3K27ac (Abcam, ab4729). 1.7 μg chromatin as quantitated by qubit dsDNA HS Kit (Invitrogen Q32854) were used per chromatin immunoprecipitation and immunoprecipitation was performed overnight 4 °C with inversion. After washing steps, DNA was eluted using phenol chloroform isoamyl alcohol (Thermo Scientific J62336) isolation and ethanol precipitation then quantitated using qubit dsDNA HS Kit (Invitrogen Q32854) and submitted to Sanford Burnham Prebys genomic core for library preparation and sequencing on Element Biosciences AVITI sequencer.

ChIP–seq was performed as following described: Library preparation of ChIP DNA was performed with the Watchmaker DNA Library Prep Kit (Watchmaker Genomics, 7K0103) with xGEN Stubby adaptors (IDT, 10005924) and xGEN 10nt UDI Primers (IDT, 10008052). Libraries were sequenced (2× 76 bp) with the Element Biosciences AVITI Sequencing platform using the AVITI 2× 75 High Output Cloudbreak Freestyle Kit (Element Biosciences, 860-00015).

### Generation of ChIP–seq analysis files

For ChIP–seq in Parkin cells and Sen (IR) cells treated by DMSO or CTPI2: H3K27ac and H3 ChIP–seq datasets were processed using the nf-core/chipseq Nextflow pipeline (v2.0.0) with Singularity containers and the parameters --aligner bowtie2, --read_length 75, and --genome GRCh38. H3K27ac bigWig signal tracks were normalized to H3 signal using deepTools (v3.5.5)^33^ bigwigCompare with the parameters --operation subtract and --binSize 10.

H2K27ac and H3 ChIP–seq samples were processed with nf-core chipseq nextflow pipeline version 2.0.0 (ref. ^34^) using singularity containers and parameters “--aligner bowtie2 --read_length 75 --genome GRCh38”. H3K27ac bigwig signal files were normalized by H3 signal using Deeptools bigwigCompare version 3.5.5 (ref. ^33^) with parameters “—operation subtract --binSize 10”.

For the Sen (IR) *BAX*/*BAK1* CRISPR and Sen (IR) CRISPR empty vector experiment: Paired-end H3K27ac and H3 samples were trimmed using Cutadapt v5.2 with parameters “-m 25 -q 30,30 --nextseq-trim=30 -a AGATCGGAAGAGCACACGTCTGAACTCCAGTCAC -A AGATCGGAAGAGCGTCGTGTAGGGAAAGAGTGT”. Trimmed reads were processed using nf-core chipseq^34^ nextflow pipeline version 2.0.0 with apptainer containers and parameters “-profile singularity --aligner bowtie2 --read_length 150 --genome GRCh38 --macs_fdr 0.01 --skip_trimming true”. The nf-core chipseq pipeline performed alignment, alignment filtering, and peak-calling. Normalized signal files for H3K27ac samples were generated in bigwig format using bamCoverage from deepTools v3.4.3 (ref. ^35^) with parameters “--binSize 5 --normalizeUsing RPGC --extendReads --ignoreForNormalization chrX --effectiveGenomeSize 2913022398 --scaleFactor {sample-specific-scale-factor}”. Sample-specific scale factors were calculated for each sample by dividing number of reads within peaks (obtained from nf-core pipeline featureCounts summary table) by number of de-duplicated reads (fraction of reads in peaks, also called FriP). Scale factors were computed relative to sample with highest FRiP. Normalized signal files for H3 samples were generated using bamCoverage with parameters “--binSize 5 --normalizeUsing RPGC --extendReads --ignoreForNormalization chrX --effectiveGenomeSize 2913022398 –scaleFactor 1.0”. H3K27ac bigwig signal files were normalized by H3 signal using deepTools bigwigCompare with parameters “--operation subtract --binSize 5”. H3K27ac bigwig signal average files for biological replicates were generated using deepTools bigwigCompare with parameters “--operation mean --binSize 5”.

### ChIP–seq databases analysis, track visualization and Heat map generation

Previously published ChIP–seq datasets (GSE106146, GSE56307, GSE74238 and GSE103590), together with newly generated data from this study (GSE279410), were analysed. ChIP–seq experiments performed in this work represent the average signal from three biological replicates per condition.

Genome browser tracks for selected loci were visualized using Integrative Genomics Viewer (IGV). Metaplots and heat map were generated using deepTools (computeMatrix and plotHeatmap) within the Galaxy platform.

### Mitochondrial superoxide measurements

Cells were cultured in black 96-well plates suitable for fluorescence measurements. Experiments were performed 12 days post-irradiation (day 12). Proliferating cells were seeded into assay plates one day prior to measurement to ensure optimal adherence and recovery. Mitochondrial superoxide levels were assessed using MitoSOX Red Mitochondrial Superoxide Indicator (Thermo Fisher Scientific, M36008). Cells were incubated with 5 µM MitoSOX in serum-free medium for 10 min at 37 °C, protected from light. Following staining, cells were washed twice with phosphate-buffered saline (PBS) to remove excess dye. Fluorescence was then measured in PBS at 37 °C every 2 min for 50 min using a Varioskan LUX multimode microplate reader (Thermo Fisher Scientific).

### Seahorse analysis

Cellular oxygen consumption rate (OCR) and extracellular acidification rate (ECAR) were measured using an XFe96 Extracellular Flux Analyzer (Agilent Technologies) with the Seahorse XF Cell Mito Stress Test Kit (Agilent, 103015-100), following the manufacturer’s instructions. Cells were seeded at 5,000 cells per well in Seahorse XF96 cell culture microplates and irradiated the following day. Measurements were performed 12 days post-irradiation (day 12). For proliferating control conditions, cells were seeded one day prior to the assay.

On the day of the assay, culture medium was replaced with Seahorse XF DMEM assay medium supplemented with 1 mM sodium pyruvate, 2 mM l-glutamine, and 10 mM glucose. Where indicated, CTPI2 inhibitors were maintained at the specified concentrations throughout the assay. Plates were incubated at 37 °C in a non-CO_2_ incubator for equilibration prior to measurement.

Mitochondrial function was assessed by sequential injection of the following compounds: oligomycin (1.5 µM), FCCP (1 µM), and a mixture of rotenone and antimycin A (0.5 µM each). OCR and ECAR were recorded according to the standard Mito Stress Test protocol.

### Multiplex immunoassay for cytokine quantification in conditioned media

Conditioned media were generated by culturing 80,000 cells per condition in serum-free medium for 24 h. Media were collected and centrifuged at 2,000 rpm for 5 min at 4 °C to remove cellular debris. The clarified supernatant was aliquoted and stored at −80 °C until analysis. On the day of the assay, aliquots were thawed on ice and processed immediately.

Cytokine concentrations were quantified using Luminex xMAP multiplex technology with commercially available antibody panels (Bio-Techne; FCSTM18B, LXSAHM), following the manufacturer’s instructions. Samples were diluted 1:2 in the kit-provided diluent. Minimum fluorescence intensity (MFI) was acquired using a Luminex IntelliFlex system (Luminex).

Standard curves for each analyte were generated using a five-parameter logistic (5-PL) regression model, and cytokine concentrations were calculated with Quantist software (Bio-Techne). Final cytokine levels were expressed in pg ml^−1^.

### Western blotting

Cells were lysed in lysis buffer (RIPA: 150 mM NaCl, 1% NP40, 0.5% sodium deoxycholate, 0.1% SDS, 50 mM Tris pH 7.4, 1× phosphatase and protease inhibitors cocktail in H_2_O) and the protein concentration was determined using the Bio-Rad protein assay (Bio-Rad, reagent A, 500-0113; reagent B, 500-0114; reagent C, 500-0115). Proteins were deposed by equal amount on each well and separated by molecular weight on NuPAGE 4 to 12% Bis-Tris gels (Invitrogen, NP0323). Proteins were secondary blotted on PVDF membrane using Power Blotter XL machine (Invitrogen, PB0010) and Power Blotter Select Transfer Stacks, PVDF, regular size (Invitrogen, PB5310). Membranes were blocked with TBS-Tween (TBS-T) blocking buffer (5% milk powder, 0.05% Tween-20 in TBS) and incubated with primary antibodies at 4 °C overnight (a list of the antibodies Supplementary Table 3). After washes in TBS-T, the membranes were incubated with a peroxidase-conjugated secondary antibody for at least 1 h at room temperature. The membranes were then incubated with either SuperSignal West Pico PLUS Chemiluminescent Substrate (Thermo Scientific, 34577) or the KwikQuant Western blot detection kit (Kindle Bioscience, R1100) according to manufacturer’s instructions, and visualized using iBright 1500 system from Invitrogen.

### RT–qPCR

Total RNA was extracted using QIAshredder column (Qiagen, 79656) following by RNeasy Mini Kit (Qiagen, 74106). mRNAs were quantified by spectrophotometry (NanoDrop One, Thermo Fisher Scientific). cDNAs were synthesized with the MultiScribe reverse transcriptase (High-Capacity cDNA Reverse Transcription Kit, Applied Biosystem). PCR was performed for 40 cycles with TaqMan Probes (IDT) in Perfecta qPCR Tough mix (Quantabio95112.012) and run on CFX Opus 384 Real Time PCR Detection System (Bio-Rad). Different Probes used are listed in Supplementary Table 2. TBP for human cells and HPRT for mice sample were used as a reference gene for normalization and relative gene expression, compared to the control group, was calculated using the comparative cycle threshold (CT) method (2^−∆∆CT^).

### RNA sequencing

RNA-seq was performed by Azenta Life Sciences using an Illumina platform with a paired-end configuration (2× 150 bp), generating approximately 30 million read pairs per sample. Raw sequencing reads were processed to remove adapter sequences and low-quality bases using Trimmomatic (v0.36). Quality-filtered reads were aligned to the Homo sapiens reference genome (GRCh38, Ensembl) using the STAR aligner (v2.5.2b), a splice-aware aligner capable of detecting exon–exon junctions to improve mapping accuracy. Alignment outputs were generated in BAM format.

Gene-level read counts were obtained using featureCounts from the Subread package (v1.5.2), summarizing reads overlapping annotated exon regions based on gene identifiers in the reference annotation file. Only uniquely mapped reads were included. For strand-specific libraries, reads were counted in a strand-aware manner as appropriate.

Differential gene expression analysis was performed in R using DESeq2. Comparisons were made between Sen (IR) and Sen (IR) + CTPI2 conditions. The Wald test was used to estimate statistical significance and log_2_ fold changes. Genes with a nominal *P* value < 0.05 and an absolute log_2_ fold change > 0.5 were considered differentially expressed.

For visualization, transcripts per million (TPM) values were used to generate z-score–normalized expression matrices for heat map representation across samples.

### Immunocytochemistry

Cells were cultured on coverslip and fixed for 10 min using either 4% PFA in PBS for usual staining or 4% PFA with 0.2% of glutaraldehyde for mitochondrial network staining. After 3 PBS washes, cells were blocked and permeabilized for at least 1 h at room temperature with blocking buffer (PBS 0.3% Tritonx100, 5% BSA and 1:60 normal goat serum). Cells were incubated with primary antibodies overnight at 4 °C in humid chamber. After PBS washes, cells were incubated with secondary antibodies for at least 1 h at room temperature. After final washes, coverslips were mounted onto glass microscope slides with ProLong Gold Antifade Mountant with DAPI (Invitrogen). A list of the antibodies used is provided in Supplementary Table 3.

Pictures were taken with Leica widefield microscope DMi8 either at ×20 or ×63 magnification. Images were quantified using ImageJ software (v1.54p).

Formalin-fixed, paraffin-embedded tissue sections (3 µm) were deparaffinized in xylene (2 times for 5 min each) and hydrated using sequentially 5 min batch of 100% ethanol (twice), 90% ethanol, 70% ethanol and distilled water (twice). Antigen retrieval was performed by heating the sections to 98 °C in citrate buffer at pH 6.0 for 15 min. The slides were allowed to cool down for 30 min and were then rinsed in PBS twice for 5 min. To avoid nonspecific binding, tissue sections were blocked for at least 30 min at room temperature in the blocking solution (PBS, 0.1% BSA with 1:60 of normal goat serum). For membrane staining, WGA was applied for 30 min at room temperature and wash 3 times with PBS. Primary antibodies were incubating in blocking solution overnight at 4 °C in humid chamber. After several PBS washes, tissue sections were incubated with secondary antibodies for at least 1 h at room temperature. Finally, sections were mounted with ProLong Gold Antifade Mountant with DAPI (Invitrogen). A list of the antibodies used is provided in Supplementary Table 3.

Pictures were taken with Leica widefield microscope DMi8 either at ×20 or ×40 magnification. Images were quantified using ImageJ software (v1.54p).

### Mouse models and treatments

All animal experiments were performed according to protocols approved by the Institutional Animal Care and Use Committee (IACUC) at Mayo Clinic. Male and female aged wild-type C57BL/6 mice (aged 19 months) were acquired from the National Institute on Aging (NIA) and were maintained in a pathogen-free facility under a 12 h:12 h light:dark cycle at 23–24 °C with free access to regular chow and water. The mice were housed in same-sex cages in groups of 5. The animals were randomly assigned into the vehicle or treatment group. Mice were gavaged with 50 mg kg^−1^ of CTPI2 (MedChem Express, HY-123986) diluted in corn oil 3 times a week for 3 months (from 19 months to 22 months old), at which point the animals were euthanized and tissues were collected for analysis. Frailty assessment, tightrope test and hanging test was conducted before and after the 3 months treatments. Grip strength was asset before, at mid-point (1.5 months) and after 3 months of treatment. The mice were euthanized and considered to be dead if they met humane end points.

For the validation of the p16 antibody in mice, p16 luciferase reporter mice (*p16*^*Luc/Luc*^) Alb.C57Bl6 mice and littermate control *p16*^*+/+*^ mice were used^36^. Mice were intraperitoneally injected with a single dose doxorubicin at 10 mg kg^−1^ or vehicle (NaCl), and tissues were collected at day 45 post-injection.

### Stromal cells isolation

Hearts and kidneys were collected from mice after intraventricular perfusion of 10 ml PBS, minced with scalpels and digested with Liberase TM (Roche) diluted in RPMI1640 (GIBCO) as described previously^24^. Briefly, digestion of tissue fragments was performed by two successive incubations for 10 min with enzymatic solution at 37 °C under shaking and stopped by the addition of heat-inactivated fetal bovine serum (Gibco). Single-cell suspensions were obtained by filtrations on 100 µm and 40 µm cell strainers (BD Falcon). Cells were either pelleted and lysed for RNA extraction or seeded for in vitro experiment.

### Frailty measurements

Frailty was assessed based on a 31-items performance-based frailty index that reflects on clinical signs of deterioration in mice, as described^22^. These clinical assessments include evaluation of integument, the musculoskeletal system, the vestibulocochlear/ auditory systems, the ocular and nasal systems, the digestive system, the urogenital system, the respiratory system, signs of discomfort, body weight, and body surface temperature. The severity of each parameter was rated as follows: a score of 0 was given for mice displaying no sign of frailty, 0.5 was given for mild deficits, and a score of 1 was given if severe deficits were observed.

### Neuromuscular coordination analysis

Neuromuscular coordination and balance were assessed using the tightrope test. Mice were placed on a horizontal bar (1.5 cm diameter) elevated 60 cm above the floor. The latency to fall was recorded, with a maximum cut-off time of 60 s per trial. A trial was considered successful if the mouse remained on the bar for the full 60 s without falling. Each mouse underwent up to five trials separated by 30 s rest intervals. Testing was terminated once the mouse either completed five trials or achieved a cumulative time of 60 s on the bar.

### Forelimb grip strength analysis

Forelimb grip strength was assessed by allowing mice to grasp a suspended wire coat hanger measuring 2 mm in diameter and 30 cm in length. The time each mouse was able to hang from the wire using its forelimbs was recorded. Each mouse was given 10 attempts, with 20 s of rest between attempts, to reach a cumulative hanging time of 90 s. Successful performance was defined as achieving a total of 90 s of hanging time, while a trial was considered a failure if the mouse fell from the wire.

### Skeletal imaging

All bone imaging and analysis was performed in a blinded manner. Quantitative analysis of the lumbar spine (L4–L6) and distal femoral metaphysis were performed using the Viva Scan 40 μCT scanner (Scanco Medical AG, Basserdorf, Switzerland) with the following parameters: 55 kVp, 145 mA, high resolution, 21.5 diameter, 10.5 μm voxel size, 300 ms integration time. Using two dimensional (2D) data from scanned slices, 3D analysis was used to calculate morphometric parameters at both the lumbar spine (200 slices) and distal femoral metaphysis (100 slices) defining trabecular bone mass and microarchitecture, including trabecular bone volume fraction (BV/TV; %), trabecular number (Tb.N; 1/mm), trabecular thickness (Tb.Th; mm), trabecular separation (Tb.Sp; mm [higher values are associated with weaker bone]), and the structure model index (SMI), which indicates whether trabeculae are stronger, plate-like (lower values) or weaker, rod-like (higher values). Cortical thickness (Ct.Th; mm) was assessed at the distal femoral metaphysis (50 slices). Micro-finite element analysis (μFEA) was performed at the femoral metaphysis to assess failure load (N (bone strength)) using the manufacture’s software (Scanco Medical; Finite Element-Software v1.13). All μCT parameters were derived using the manufacturer’s protocols.

### Plasma cytokine array

Blood was collected via cheek bleeding into EDTA-coated tubes. Samples were centrifuged at 4,000*g* for 20 min at 4 °C and supernatant (plasma) was collected. Plasma cytokine and chemokines levels were quantified by Eve Technologie using the Mouse High Sensitivity 18-Plex Discovery Assay.

### Intra peritoneal glucose tolerance test

Mice were fasted for 16 h, after which fasting glucose levels were measured from tail blood. They then received an intraperitoneal injection of d-glucose (Sigma, 68270) at 2 g kg^−1^, and blood glucose levels were recorded with a glucometer at 15, 30, 45, 60, 90 and 120 min following the injection.

### Metabolic analyses using whole-body indirect calorimetry

A 16-chamber Comprehensive Animal Monitoring System (CLAMS, Columbus Instruments) was used to measure oxygen consumption (VO_2_) and carbon dioxide production (VCO_2_) of individual mice, as previously described^37^. The mice were maintained under a 12:12 h light:dark cycle in a temperature-controlled room (22–24 °C). Oxygen and carbon dioxide analysers were calibrated with certified primary standard calibration gas. Mice were placed individually into the CLAMS chambers and allowed to acclimate for 14–18 h followed by the start of the 48-h experimental period beginning at 6 AM. Measurements were conducted for 24 h of fed and 24 h of fasted conditions. The respiratory exchange ratio (RER) was calculated from VO_2_ and VCO_2_ values. Energy expenditure was calculated using the equation [(3.815 + (1.232 × RER)) × VO_2_]. Food intake was measured as part of the cage calorimetry experiments using an integrated balance accounting for spillage of food. Physical activity levels were measured by photocells.

### VetScan

Blood was collected via cheek bleeding into lithium/heparin-coated tubes. Samples were centrifuged at 4,000*g* for 20 min at 4 °C and supernatant (plasma) was collected. One hundred microlitres of plasma was analysed using the Comprehensive Diagnostic Profile (Zoetis, 10023220) according to the manufacturer’s protocol and read on the VETSCAN VS2 Chemistry Analyzer.

### Triglyceride assay in muscle

Triglyceride levels were measured in quadriceps muscle samples using a minimum of 10 mg of tissue per sample, according to the manufacturer’s instructions for the Triglyceride Assay Kit (Abcam, ab178780).

### Multiome

#### Nuclei isolation

For the multiome experiment, quadriceps muscles were collected from 2 pooled groups of four 22-month-old C57Bl/6 mice, treated for 3 months with either vehicle or CTPI2 (50 mg kg^−1^). Approximately 25 mg of frozen tissue from each pooled sample was used for nuclei isolation. The nuclei isolation was performed at 4 °C using a Singulator 200 instrument (S2 Genomics) with a regular cartridge, according to the manufacturer’s instructions. The Single Shot Extended Nuclei Isolation program was used with a 10 min incubation time and the mixing type modified for trituration. Nuclei isolation was performed in the presence of 1 U μl^−1^ of RNase inhibitor (Roche). After incubation, the samples were passed through a 40 µm Flowmi cell strainer (Electron Microscopy Sciences), and the flow through was then centrifuged at 500*g* for 5 min at 4 °C. After centrifugation, the nuclei were resuspended in diluted nuclei buffer. The nuclei concentration was assessed by PI staining using a Cellometer K2 cell counter. Between 8,000 and 10,000 nuclei were targeted for capture and used for single-nucleus ATAC–seq (snATAC–seq) plus single-nucleus RNA-seq (snRNA-seq).

#### snATAC–seq and snRNA-seq

snATAC–seq plus snRNA-seq was performed using the 10x Genomics platform. Between 8,000 and 10,000 nuclei per sample were subjected to transposase assays before proceeding to single-cell partitioning into gel beads in emulsion, barcoding and pre-amplification according to the manufacturer’s instructions. ATAC library construction and cDNA, followed by GEX library construction, were also done following established 10x Genomics protocols. The libraries’ concentration was measured using Qubit High Sensitivity assays (Thermo Fisher Scientific), and library profiles were assessed in a fragment analyser (Agilent) before sequencing. The snATAC and snRNA libraries were sequenced for 50 bp and 100 bp paired-end sequencing, respectively, on a NovaSeq X 10B instrument (Illumina) before demultiplexing and alignment to the reference mouse genome.

### Multiome analysis

The snATAC–seq + snRNA-seq data were processed using CellRanger-ARC (v2.0.2), Seurat (v5.3.1) and Signac (1.16.0). Sequenced reads from the GEX and ATAC droplet libraries were aligned and quantified using 10x Genomics Cell Ranger ARC v2.0.2. The reads were aligned to the pre-built mouse reference genome GRCm39-2024-A provided by 10X Genomics. GEX and ATAC count matrices from each sample were merged independently using Seurat. GEX count matrix was log-normalized, scaled to mean 0 and variance 1, and dimensionality reduction was performed using principal components analysis on the top 2,000 most highly variable genes. UMAP for GEX was calculated using the top 50 principal components. Open chromatin peaks called per sample were merged using reduce function from GenomicRanges (v1.56.2) R package, then the ATAC fragment count matrix was recalculated using Signac. Merged peaks that were smaller than 20 base pairs or larger than 10,000 base pairs were removed from analysis. The ATAC count matrix was normalized using term frequency − inverse document frequency (TF-IDF) and dimensionality reduction performed using singular value decomposition (SVD) using only peaks with non-zero counts in at least 20 cells - together known as latent semantic indexing (LSI) that generates LSI components. The UMAP for ATAC was calculated using LSI components 2 to 50. Seurat’s WNN algorithm was used on principal components 1 to 50 (GEX) and LSI components 2 to 50 (ATAC) together to obtain a combined UMAP projection of both modalities. Cells with more than 20% of reads mapped to mitochondrial genes, those with less than 200 unique genes detected (GEX), those with less than 200 unique peaks detected (ATAC) and those with TSS enrichment score (as calculated by Signac) less than 1 were removed for quality control. After QC filtering, the resulting data included 13,400 cells from 2 samples.

Cell-type identification was performed by unsupervised clustering using a shared nearest neighbour modularity optimization-based clustering algorithm performed by Seurat’s FindNeighbors and FindClusters functions followed by marker validation. Markers for each cluster were identified using Seurat’s FindAllMarkers function. Cell type identification followed commonly used markers for key cell types^38,39^.

Sen-like cells were identified by scoring each cell based on their expression of either CoreScence^25^ gene set or GenAge^26^ using AddModuleScore function. Cells in the top quartile of the CoreScence score distribution or on the top 20% of the GenAge score distribution were classified as Sen-like cells.

Gene activity scores (the chromatin accessibility associated with each gene) were calculated using Signac’s GeneActivity function. Differential analysis of gene expression (GEX) and gene activity (ATAC) was performed using FindMarkers function with parameters: min.pct = 0.

### Multiplex immunohistochemistry

Paraffin-embedded sections were dewaxed, rehydrated, and subjected to antigen retrieval in citrate buffer (pH 6.0). Endogenous peroxidase was quenched using Dako Dual Endogenous Enzyme Block (Agilent, S2003) for 10 min. Sections were blocked in TSA buffer and photobleached with H_2_O_2_ under LED illumination for 5 min. Slides were incubated overnight at 4 °C with anti-p16 (ab211542, Abcam; 1:1,000), followed by SignalStain Boost HRP secondary (Cell Signaling Technology) for 15 min and Opal fluorophore (Akoya) for 15 min. After washing, antigen retrieval (citrate buffer, pH 6.0) was repeated. Sections were blocked and incubated for 1 h at room temperature with anti-p21 (ab188224, Abcam; 1:1,000), followed by HRP secondary and Opal fluorophore as above. Nuclei were counterstained with DAPI, and slides were imaged on a Leica DMi8 microscope.

#### Subsequent cycles without TSA amplification

Slides were washed, photobleached (H_2_O_2_, LED, 5 min), and blocked in PBS containing BSA and normal goat serum for 30 min. Sections were incubated for 1 h at room temperature with anti-PCNA (mouse, ab29, Abcam; 1:500) and anti-acetyl-lysine (rabbit, 9441S, Cell Signaling Technology; 1:200), followed by Alexa Fluor-conjugated secondary antibodies (goat anti-mouse 594, goat anti-rabbit 488; 1:1,000) for 1 h. After washing, nuclei were stained with DAPI and slides were imaged on a Leica DMi8 microscope. Multiplex images were analysed using SenoQuant, an automated image analysis pipeline for senescence marker quantification (GitHub: https://github.com/HaamsRee/senoquant).

### Human data analysis

Bulk RNA-seq data for human tissues were obtained from the GTEx Portal (Analysis V11; gene-level TPM values)^40^. A mitochondrial-dependent SASP score was computed for each sample as the mean TPM expression of the 51 SASP genes included in this study. Correlation *P* values controlling for age were obtained using multiple linear regression performed in Prism software (v10.0).

### Graphics

Schematic illustrations for experimental design and organ-specific diagrams were created with biorender.com for Figs. 1a,f, 2a, 3a, 4a, 5a,e,m, 6a and 7a,l. Extended Data Figs. 3a, 4a, 6g, 8k, 10g,k, 12m,q,s and 13a,e,i,m and Supplementary Fig. 1a,b.

### Statistical analysis

GraphPad Prism v.10.0 was used for statistical analysis; the results were considered to be statistically significant when *P* ≤ 0.05; NS was used to indicate non-significant results. For normally distributed data, the differences between two groups were tested for statistical significance using an independent-sample two-tailed *t*-tests. For data that were normally distributed and when there was more than one group, one-way ANOVA was used, with Tukey’s comparison post hoc test. Where data were not normally distributed, Mann–Whitney *U* tests were used to determine statistical significance.

### Ethics statement

All animal experiments were performed according to protocols approved by the Institutional Animal Care and Use Committee (IACUC) at Mayo Clinic.

### Reporting summary

Further information on research design is available in the Nature Portfolio Reporting Summary linked to this article.

## Online content

Any methods, additional references, Nature Portfolio reporting summaries, source data, extended data, supplementary information, acknowledgements, peer review information; details of author contributions and competing interests; and statements of data and code availability are available at 10.1038/s41586-026-10791-2.

## Supplementary information

Supplementary Information Supplementary Figs. 1 and 2 and Supplementary Tables 1–3. Supplementary Fig. 1: Pharmacological inhibition of the mitochondrial citrate carrier SLC25A1 reduces SASP expression in aged stromal cells ex vivo. **a**,**b**, Schematic representation of the experimental procedure, including isolation of cardiac stromal cells and renal cells from young and aged mice. **c**,**d**, Relative mRNA expression of SASP components *Il6* and *Cxcl1* (**c**) and cell cycle inhibitor p16 (**d**) in cardiac stromal cells from young and aged hearts treated with two concentrations of CTPI2 (15 and 30 μM), expressed as fold change relative to young stromal cells (Young) (*n* = 5). **e**,**f**, Relative mRNA expression of SASP components *Ccl2* and *Tnf* (**e**) and p16 (**f**) in renal cells from young and aged kidneys treated with two concentrations of CTPI2 (15 and 30 μM), expressed as fold change relative to young renal cells (Young) (*n* = 5). Data are presented as mean ± s.em. from 5 mice per group. Statistical tests: one-way ANOVA with Tukey’s multiple comparison test (*n* = independent experiment). *P* values as indicated in the figure. ns, non significant, *P* > 0.05. Supplementary Fig. 2: Validation of p16 immunostaining specificity in mouse liver using p16-knockout mice. Representative immunofluorescence staining for p16 and p21 in liver sections from wild-type (WT) and p16-knockout (p16KO) mice 45 days after intraperitoneal doxorubicin administration, demonstrating the specificity of the p16 antibody. Scale bar, 50 μm (experiment repeated once).

Reporting Summary

Peer Review File

## Source data

Source Data Figs. 1–7 and Source Data Extended Data Figs. 1–13

## Data Availability

Datasets generated and analysed during the current study are available in the GEO repository: GSE279410 and GSE326624 for the ChIP–seq, GSE279411 and GSE319937 for the RNA-seq, and GSE324238 for the multiome (snATAC–seq and snRNA-seq). Bulk RNA-seq data for human tissues were obtained from the GTEx Portal (Analysis V11; gene-level TPM values). Source data are provided with this paper.
