# Supplementary material for: Mitochondrial metabolism and epigenetic crosstalk drive SASP
Source: Nature. 2026 Jul 29;656(8129):980–92. doi: 10.1038/s41586-026-10791-2 (PMC13518223; doi:10.1038/s41586-026-10791-2)
Supplement: Supplementary file 1 [file 41586_2026_10791_MOESM1_ESM.pdf]

nature portfolio

https://doi.org/10.1038/s41586-026-10791-2

Supplementary information

Mitochondrial metabolism and epigenetic crosstalk drive SASP

In the format provided by the authors and unedited

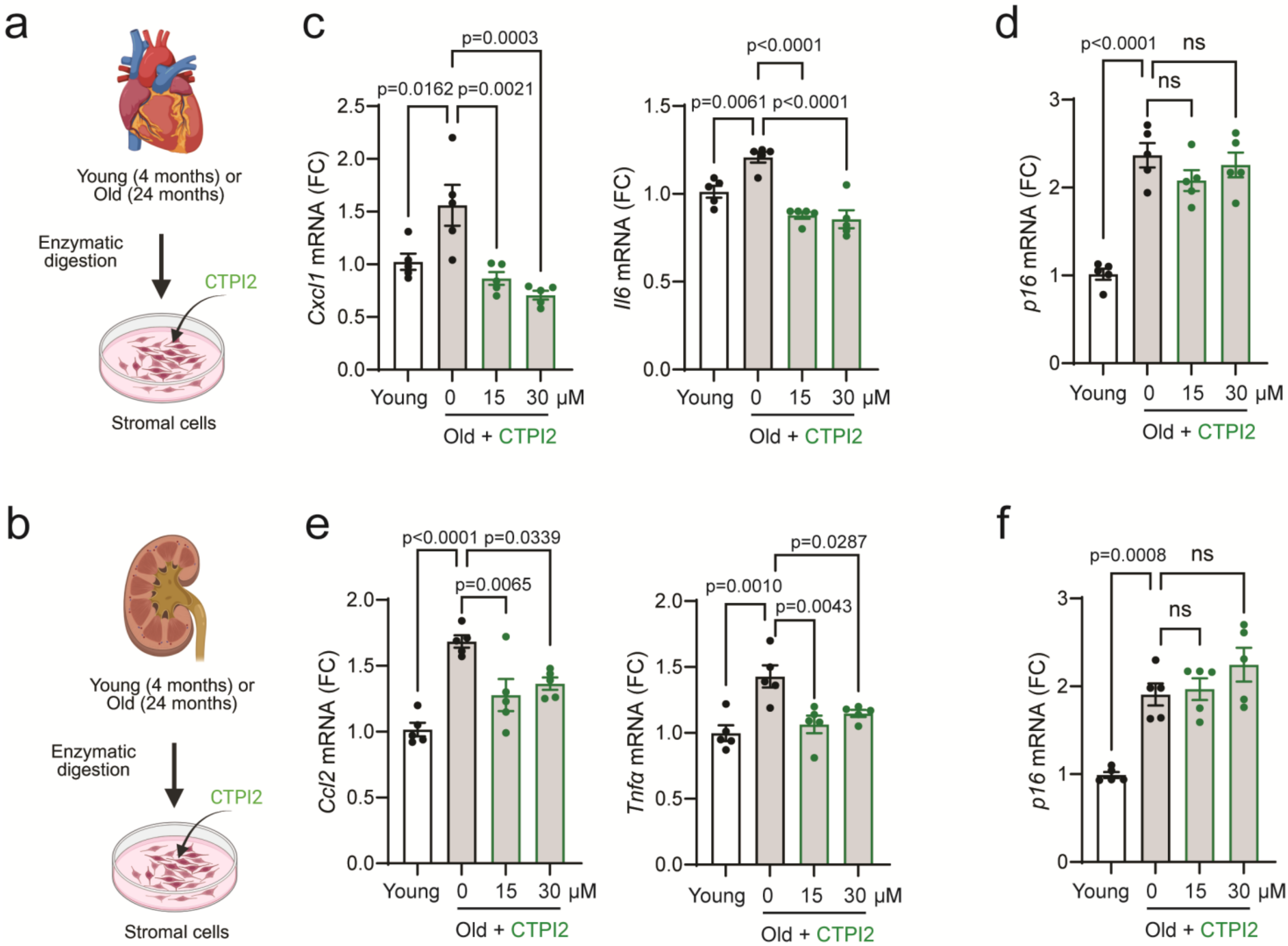

Supplementary Figure 1: Pharmacological inhibition of the mitochondrial citrate carrier SLC25A1 reduces SASP expression in aged stromal cells *ex vivo.*

(a-b) Schematic representation of the experimental procedure, including isolation of cardiac stromal cells and renal cells from young and aged mice. **(c-d)** Relative mRNA expression of SASP components *Il6* and *Cxcl1* **(c)**, and cell cycle inhibitor p16 **(d)** in cardiac stromal cells from young and aged hearts treated with two concentrations of CTPI2 (15 and 30µM), expressed as fold change relative to young stromal cells (Young) (n=5). **(e-f)** Relative mRNA expression of SASP components *Ccl2* and *Tnfα* **(e)**, and p16 **(f)** in renal cells from young and aged kidneys treated with two concentrations of CTPI2 (15 and 30µM), expressed as fold change relative to young renal cells (Young) (n=5). Data are presented as Mean ± s.em. from 5 mice per group. Statistical tests: One-way ANOVA with Tukey's multiple comparison test. (n= independent experiment). P-values as indicated in the figure. ns= non significant p>0.05.

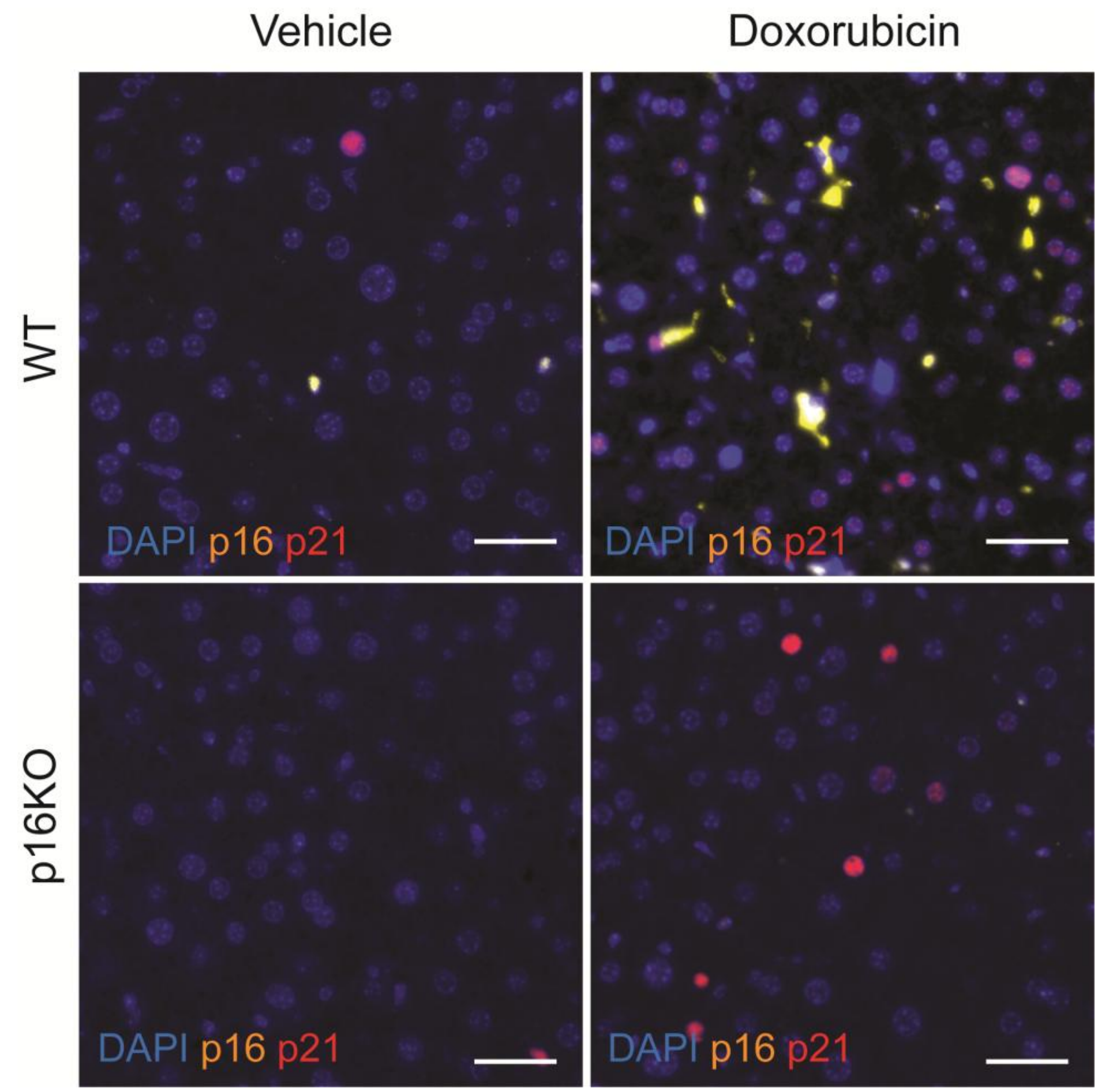

Supplementary Figure 2: Validation of p16 immunostaining specificity in mouse liver using p16 knockout mice. Representative immunofluorescence staining for p16 and p21 in liver sections from wild-type (WT) and p16 knockout (p16KO) mice 45 days after intraperitoneal doxorubicin administration, demonstrating the specificity of the p16 antibody. Scale bar= 50μm (experiment repeated once).

a

	Female			Male		
	DMSO	CTPI2		DMSO	CTPI2	
	Mean (n=9)	Mean (n=10)	p-value	Mean (n=10)	Mean (n=9)	p-value
**Femur Metaphyse Trabecular Bone**						
Bone volume fraction (BV/TV)	1.018	0.862	0.515	5.540	5.506	0.963
Trabecular Number (Tb.N)	1.405	1.400	0.947	2.307	2.348	0.790
Trabecular Thickness (Tb.Th)	0.066	0.068	0.692	0.065	0.059	0.125
**Femur Metaphyse Cortical Bone**						
Volume bone mineral density (vBMD)	933.990	926.853	0.755	895.805	882.831	0.593
Cortical Thickness (Ct.Th. mm)	0.144	0.140	0.723	0.143	0.126	0.038
Endocortical Circumference (EC, mm)	4.912	4.975	0.622	5.343	5.553	0.233
Periostal Circumference (PC, mm)	5.816	5.858	0.633	6.243	6.345	0.481
**Femur Diaphyse Cortical Bone**						
Volume bone mineral density (vBMD)	1131.089	1114.828	0.035	1074.985	1088.039	0.562
Cortical Thickness (Ct.Th. mm)	0.209	0.204	0.439	0.205	0.204	0.913
Endocortical Circumference (EC, mm)	3.774	3.757	0.750	4.081	4.165	0.416
Periostal Circumference (PC, mm)	5.089	5.042	0.450	5.368	5.449	0.353
**Spine Trabecular Bone**						
Bone volume fraction (BV/TV)	13.030	14.250	0.536	15.807	15.577	0.892
Trabecular Number (Tb.N)	2.075	2.270	0.216	3.166	3.330	0.458
Trabecular Thickness (Tb.Th)	0.065	0.069	0.355	0.059	0.059	0.788

Supplementary Table 1: Pharmacological inhibition of the mitochondrial citrate carrier SLC25A1 during aging does not affect bone microarchitecture.
Table summarizing various bone parameters analyzed in vehicle- and CTPI2-treated mice.

	Human	
	Name	ref
CDKi	*CDKN2a (p16)*	Hs.PT.58.40743463.g
	CDKN2b (p21)	HS.PT.58.38492863.g
	CDKN2c (p15)	Hs.PT.58.4919581
SASP	*IL6*	Hs.PT.58.40226675
	IL8	Hs.PT.58.1518186
	IL1b	HS.PT.58.1518186
	CCL2	HS.PT.58.45467977
	CCL20	Hs.PT.58.19600309
	CXCL10	Hs.PT.58.3790956.g
	CX3CL1	Hs.PT.58.19601997
	MMP2	Hs.PT.58.39114006
	TGFb1	Hs.PT.58.39813975
	OAS1	Hs.PT.58.2338899
	ISG15	Hs.PT.58.39185901.g
Other	*MPC1*	Hs.PT.58.40015928
	MPC2	Hs.PT.58.2805713
	SLC25a1 (CIC)	Hs.PT.58.22671354
	ACLY	Hs.PT.56a.4367337.g
House keeping	*TBP*	HS.PT.39a.22214825

	Mice	
	Name	ref
CDKI	*Cdkn2a (p16)*	Mm.PT.58.42804808
	Cdkn2b (p21)	Mm.PT.58.5884610
SASP	*Ccl2*	Mm.PT.58.42151692
	Cxcl1	Mm.PT.58.8767901
	Cxcl10	Mm.PT.58.43575827
	Cxcl13	Mm.PT.58.42142828
	Edn3	Mm.PT.58.32657883
	Gdf15	Mm.PT.58.13112185
	Igfbp2	Mm.PT.58.21635888
	Il1a	Mm.PT.58.32778767
	Il1b	Mm.PT.58.41616450
	Il6	Mm.PT.58.10005566
	Mmp3	Mm.PT.58.9719290
	Tnfa	Mm.PT.58.12575861
Macrophage	*Ccr2*	Mm.PT.58.14116710
	CD45	Mm.PT.58.7583849
	Cd68	Mm.PT.58.32698807
Lipid Metabolism	*Mcat*	Mm.PT.58.5230608
	Acaca	Mm.PT.58.12492865
	Fasn	Mm.PT.58.14276063
House keeping	*Hprt*	Mm.PT.39a.22214828

Supplementary Table 2: qPCR Primer Probes used from IDT

Western blot			
Name	Vendor	Ref	Dilution
UQCRC2	Abcam	ab14745	1/1000
NDUFB8	Abcam	ab110242	1/1000
p16	Cell Signalling	92803S	1/1000
p21	Cell Signalling	2947S	1/1000
H3K27ac	Abcam	ab4729	1/1000
H3K9ac	Abcam	ab4441	1/1000
H2BK20ac	Cell Signalling	34156S	1/1000
H3 total	Abcam	ab1791	1/1000
MPC1	Cell Signaling	14462S	1/1000
CyclinA	Santa Cruz	sc-271682	1/500
SLC25A1	Protein Tech	15235-1-AP	1/1000
STING	Cell Signalling	13647S	1/1000
BAX (D2E11)	Cell Signalling	5023T	1/1000
BAK (D4E4)	Cell Signalling	12105	1/1000
β-Tubulin	Cell Signalling	5568BC	1/1000
GAPDH	Cell Signalling	5174S	1/1000
Actin	Sigma	A2066	1/1000

Secondary			
HRP rabbit	R&D Systems	HAF005	1/5000
HRP mouse	R&D Systems	HAF007	1/5000

ChIP			
Name	Vendor	ref	Dilution
Histone H3	Abcam	ab1791	5µg
H3K27ac	Abcam	ab4729	5µg

Immunofluorescence			
Name	Vendor	ref	Dilution
Acetylated Lysine	Cell Signaling	9441S	1/200
TOMM20	BD Biosciences	612278	1/200
TOMM20	Sigma-Aldrich	HPA011562	1/200
Click-iT™ EdU	Invitrogen	C10339	N/A
KI67	Abcam	ab15580	1/500
HMGB1	Cell Signalling	3935S	1/500
γH2AX	Millipore	05-636	1/500
WGA AF647	Invitrogen	W32466	1/200
DNA	Progen	690014S	1/250
p16	Abcam	ab211542	1/1000
p21	Abcam	ab188224	1/1000
PCNA	Abcam	ab29	1/500

Secondary			
AlexaFlour 488 goat anti-rabbit IgG	Invitrogen	A11034	1/1000
AlexaFluor 488 goat anti-mouse IgG	Invitrogen	A11001	1/1000
AlexaFluor 594 goat anti-mouse IgG	Invitrogen	A11032	1/1000
AlexaFluor 594 goat anti-rabbit IgG	Invitrogen	A11037	1/1000

TSA amplification			
SignalStain® Boost IHC Detection Reagent (HRP, Rabbit)	Cell Signaling	8114S	N/A
OPAL 570	Perkin Elmer	FP1488A	1/200
OPAL 650	Perkin Elmer	OP001005	1/200

Supplementary Table 3: Antibodies used.
