# Supplementary material for: Mitochondrial metabolism and epigenetic crosstalk drive SASP
Source: Nature. 2026 Jul 29;656(8129):980–92. doi: 10.1038/s41586-026-10791-2 (PMC13518223; doi:10.1038/s41586-026-10791-2)
Supplement: Supplementary file 2 [file 41586_2026_10791_MOESM2_ESM.pdf]

nature portfolio

Corresponding author(s): Joao F. Passos, Peter D. Adams

Last updated by author(s): May 27, 2026

Reporting Summary

Nature Portfolio wishes to improve the reproducibility of the work that we publish. This form provides structure for consistency and transparency in reporting. For further information on Nature Portfolio policies, see our Editorial Policies and the Editorial Policy Checklist.

Statistics

For all statistical analyses, confirm that the following items are present in the figure legend, table legend, main text, or Methods section.

n/a	Confirmed	
☐	☒	The exact sample size (*n*) for each experimental group/condition, given as a discrete number and unit of measurement
☐	☒	A statement on whether measurements were taken from distinct samples or whether the same sample was measured repeatedly
☐	☒	The statistical test(s) used AND whether they are one- or two-sided<br>*Only common tests should be described solely by name; describe more complex techniques in the Methods section.*
☐	☒	A description of all covariates tested
☐	☒	A description of any assumptions or corrections, such as tests of normality and adjustment for multiple comparisons
☐	☒	A full description of the statistical parameters including central tendency (e.g. means) or other basic estimates (e.g. regression coefficient) AND variation (e.g. standard deviation) or associated estimates of uncertainty (e.g. confidence intervals)
☐	☒	For null hypothesis testing, the test statistic (e.g. *F*, *t*, *r*) with confidence intervals, effect sizes, degrees of freedom and *P* value noted<br>*Give P values as exact values whenever suitable.*
☒	☐	For Bayesian analysis, information on the choice of priors and Markov chain Monte Carlo settings
☒	☐	For hierarchical and complex designs, identification of the appropriate level for tests and full reporting of outcomes
☐	☒	Estimates of effect sizes (e.g. Cohen's *d*, Pearson's *r*), indicating how they were calculated

Our web collection on statistics for biologists contains articles on many of the points above.

Software and code

Policy information about availability of computer code

Data collection	Image J (v1.54p), SenoQuant (v1.0.0-beta1)
Data analysis	GraphPad Prism v10, Galaxy website (https://usegalaxy.org/), CellRanger-ARC (v2.0.2), Seurat (v5.3.1) and Signac (1.16.0), IsoPat2 (2007), Agilent MassHunter Workstation Analysis (v.11), Agilent MSD ChemStation Data Analysis software (version F.01.03), Deeptools bigwigCompare (v3.5.5), deepTools (v3.4.3), Integrative Genomics Viewer (https://igv.org/app/, v2.4.3), Quantist software (v2.0.0), Trimmomatic (v0.36), STAR aligner (v2.5.2b), Finite Element-Software Version 1.13,

For manuscripts utilizing custom algorithms or software that are central to the research but not yet described in published literature, software must be made available to editors and reviewers. We strongly encourage code deposition in a community repository (e.g. GitHub). See the Nature Portfolio guidelines for submitting code & software for further information.

Data

Policy information about availability of data

All manuscripts must include a data availability statement. This statement should provide the following information, where applicable:

- Accession codes, unique identifiers, or web links for publicly available datasets
- A description of any restrictions on data availability
- For clinical datasets or third party data, please ensure that the statement adheres to our policy

The RNA-seq and ChIP-seq datasets generated and analyzed during the current study are available in the GEO repository

GSE279410
GSE279411
GSE319937
GSE324238
GSE326624

GTEx human dataset : Analysis V11; gene-level TPM values (https://gtexportal.org/home/downloads/adult-gtex/bulk_tissue_expression)

Research involving human participants, their data, or biological material

Policy information about studies with human participants or human data. See also policy information about sex, gender (identity/presentation), and sexual orientation and race, ethnicity and racism.

Reporting on sex and gender	No
Reporting on race, ethnicity, or other socially relevant groupings	No
Population characteristics	Age range (data publicly available)
Recruitment	No
Ethics oversight	No

Note that full information on the approval of the study protocol must also be provided in the manuscript.

Field-specific reporting

Please select the one below that is the best fit for your research. If you are not sure, read the appropriate sections before making your selection.

☒ Life sciences ☐ Behavioural & social sciences ☐ Ecological, evolutionary & environmental sciences

For a reference copy of the document with all sections, see nature.com/documents/nr-reporting-summary-flat.pdf

Life sciences study design

All studies must disclose on these points even when the disclosure is negative.

Sample size	For in vivo mouse studies, we have projected group size estimates based on power analyses and previous experiences.<br>For in vitro studies at least 3 independent experiment were performed which was sufficient for sample variability. For in vitro studies, at least three independent experiments were performed. For selected key experiments, the number of replicates was increased to ensure the robustness and reproducibility of the findings.
Data exclusions	no data was excluded
Replication	we tested our hypotheses using multiple approaches when feasible to enhance scientific rigor and reproducibility.<br>in vitro all experiments were performed at least 3 times independently.<br><br>For in vivo, two separate cohorts of males and females were performed, and key behavior tested were repeated (frailty, grip strength, and pole test) and show the same finding. Showing the robustness of the data.
Randomization	Animals were randomly assigned numbers before experimentations.
Blinding	Investigators were blinded to allocation during experiments and outcome assessments, and data was collected and analyzed in a blinded fashion.

Reporting for specific materials, systems and methods

We require information from authors about some types of materials, experimental systems and methods used in many studies. Here, indicate whether each material, system or method listed is relevant to your study. If you are not sure if a list item applies to your research, read the appropriate section before selecting a response.

Materials & experimental systems

n/a	Involved in the study	
☐	☒	Antibodies
☐	☒	Eukaryotic cell lines
☒	☐	Palaeontology and archaeology
☐	☒	Animals and other organisms
☒	☐	Clinical data
☒	☐	Dual use research of concern
☒	☐	Plants

Methods

n/a	Involved in the study	
☐	☒	ChIP-seq
☒	☐	Flow cytometry
☒	☐	MRI-based neuroimaging

Antibodies

Antibodies used

Western Blot
UQCRC2, Abcam ab14745, 1/1000
NDUFB8 ,Abcam ab110242, 1/1000
p16,Cell Signalling 92803S, 1/1000
p21, Cell Signalling 2947S, 1/1000
H3K27ac, Abcam ab4729, 1/1000
H3K9ac, Abcam ab4441, 1/1000
H2BK20ac, Cell Signalling, 34156S 1/1000
H3 total, Abcam ab1791, 1/1000
MPC1, Cell Signaling 14462S, 1/1000
CyclinA, Santa Cruz sc-271682, 1/500
SLC25A1, Protein Tech 15235-1-AP, 1/1000
STING,Cell Signalling 13647S, 1/1000
BAX (D2E11), Cell Signalling 5023T, 1/1000
BAK (D4E4), Cell Signalling 12105, 1/1000
β-Tubulin, Cell Signalling 5568BC, 1/1000
GAPDH, Cell Signalling 5174S, 1/1000
Actin, Sigma A2066, 1/1000

Secondary
HRP rabbit, R&D Systems HAF005, 1/5000
HRP mouse, R&D Systems HAF007, 1/5000

ChIP
Histone H3, Abcam ab1791, 5ug
H3K27ac, Abcam ab4729, 5ug

Immunofluorescence
Acetylated Lysine, Cell Signaling 9441S, 1/200
TOMM20, BD Biosciences 612278, 1/200
TOMM20, Sigma-Aldrich HPA011562, 1/200
Click-iT™ EdU Invitrogen, C10339
KI67, Abcam ab15580, 1/500
HMGB1, Cell Signalling 3935S, 1/500
γH2AX, Millipore 05-636, 1/500
WGA AF647, Invitrogen W32466, 1/200
DNA, Progen 690014S, 1/250
p16, Abcam ab211542, 1/1000
p21, Abcam ab188224, 1/1000
PCNA, Abcam ab29, 1/500

Secondary
AlexaFlour 488 goat anti-rabbit IgG Invitrogen A11034, 1/1000
AlexaFluor 488 goat anti-mouse IgG Invitrogen A11001, 1/1000
AlexaFluor 594 goat anti-mouse IgG Invitrogen A11032, 1/1000
AlexaFluor 594 goat anti-rabbit IgG Invitrogen A11037, 1/1000

TSA amplification
SignalStain® Boost IHC Detection Reagent (HRP, Rabbit) Cell Signaling 8114S
OPAL 570 Perkin Elmer FP1488A, 1/200
OPAL 650 Perkin Elmer OP001005, 1/200

Validation

All antibodies used were purchased from commercial sources and reputable vendors (eg. Abcam, Cell Signaling). We selected them based on cross-reactivity with mouse and human (depending on our research needs) and carefully evaluated the validation data provided by both the vendors and literature. In several instances, we performed further validation of antibodies by examining the

molecular weight of the band by Western blotting and Knocking out protein of interest. Antibodies were aliquoted and stored as recommended by the manufacturer to minimize freeze thaw cycles and associated loss in performance.

For p16 immunofluorescence, we validated the antibody staining in p16KO mice model.

Eukaryotic cell lines

Policy information about cell lines and Sex and Gender in Research

Cell line source(s)	IMR90, MRC5 and HEK293T cells were acquired from ATCC
Authentication	none of the cells have been authenticated
Mycoplasma contamination	all cell lines used have been tested negative for Mycoplasma contamination. (test performed regularly)
Commonly misidentified lines (See ICLAC register)	No commonly misidentified line were used for this study

Animals and other research organisms

Policy information about studies involving animals; ARRIVE guidelines recommended for reporting animal research, and Sex and Gender in Research

Laboratory animals	For this study, we used males and females from two different strain:<br>- For in vivo study: C57BL/6 mice 19 months-old acquired from the National Institute on Aging (NIA) and treated with CTPI2 or vehicle until 22 months-old (endpoint)<br>- For p16 antibody validation: 8 months-old p16 luciferase reporter mice (p16Luc/Luc) Alb.C57Bl6 and littermate control p16+/+ Alb.C57Bl6 mice were used.
Wild animals	No wild animals were used in this study
Reporting on sex	Mice from both sexes have been studies and are represented in the same graph with a different symbol between male and female. Disaggregated data were presented when sex differences are evident.
Field-collected samples	No Field-collected sample were used in this study
Ethics oversight	All animal experiments were performed according to protocols approved by the Institutional Animal Care and Use Committee (IACUC) at Mayo Clinic.

Note that full information on the approval of the study protocol must also be provided in the manuscript.

Plants

Seed stocks	N/A
Novel plant genotypes	N/A
Authentication	N/A

ChIP-seq

Data deposition

☒ Confirm that both raw and final processed data have been deposited in a public database such as GEO.

☒ Confirm that you have deposited or provided access to graph files (e.g. BED files) for the called peaks.

Data access links<br>*May remain private before publication.*	GSE279410<br>GSE326624
Files in database submission	Processed Files<br>Parkin_Prolif_Processed.bigWig<br>Parkin_IR_Processed.bigWig<br>Parkin_IR_CCCP_Processed.bigWig<br>MRC5_IR_DMSO_Processed.bigWig

MRC5_IR_CTPI2_Processed.bigWig
Bax_BAK_IR_Average.bw
Plenti_IR_Average.bw

Raw files:
APH_7_8_9_R1.fastq.gz
APH_7_8_9_R2.fastq.gz
APH_10_11_12_R1.fastq.gz
APH_10_11_12_R2.fastq.gz
APH_28_R1.fastq.gz
APH_28_R2.fastq.gz
APH_29_R1.fastq.gz
APH_29_R2.fastq.gz
APH_32_R1.fastq.gz
APH_32_R2.fastq.gz
APH_33_R1.fastq.gz
APH_33_R2.fastq.gz

H3_IR_CTPI2_1_T1_1.fastq.gz
H3_IR_CTPI2_1_T1_2.fastq.gz
H3_IR_CTPI2_2_T1_1.fastq.gz
H3_IR_CTPI2_2_T1_2.fastq.gz
H3_IR_CTPI2_3_T1_1.fastq.gz
H3_IR_CTPI2_3_T1_2.fastq.gz
H3_IR_DMSO_1_T1_1.fastq.gz
H3_IR_DMSO_1_T1_2.fastq.gz
H3_IR_DMSO_2_T1_1.fastq.gz
H3_IR_DMSO_2_T1_2.fastq.gz
H3_IR_DMSO_3_T1_1.fastq.gz
H3_IR_DMSO_3_T1_2.fastq.gz
K27ac_IR_CTPI2_1_T1_1.fastq.gz
K27ac_IR_CTPI2_1_T1_2.fastq.gz
K27ac_IR_CTPI2_2_T1_1.fastq.gz
K27ac_IR_CTPI2_2_T1_2.fastq.gz
K27ac_IR_CTPI2_3_T1_1.fastq.gz
K27ac_IR_CTPI2_3_T1_2.fastq.gz
K27ac_IR_DMSO_1_T1_1.fastq.gz
K27ac_IR_DMSO_1_T1_2.fastq.gz
K27ac_IR_DMSO_2_T1_1.fastq.gz
K27ac_IR_DMSO_2_T1_2.fastq.gz
K27ac_IR_DMSO_3_T1_1.fastq.gz
K27ac_IR_DMSO_3_T1_2.fastq.gz

IR_CCCP_1_H3K27ac_T1_1.fastq.gz
IR_CCCP_1_H3K27ac_T1_2.fastq.gz
IR_CCCP_1_H3_T1_1.fastq.gz
IR_CCCP_1_H3_T1_2.fastq.gz
IR_CCCP_3_H3K27ac_T1_1.fastq.gz
IR_CCCP_3_H3K27ac_T1_2.fastq.gz
IR_CCCP_3_H3_T1_1.fastq.gz
IR_CCCP_3_H3_T1_2.fastq.gz
IR_CCCP_4_H3K27ac_T1_1.fastq.gz
IR_CCCP_4_H3K27ac_T1_2.fastq.gz
IR_CCCP_4_H3_T1_1.fastq.gz
IR_CCCP_4_H3_T1_2.fastq.gz
Parkin_IR_1_H3K27ac_T1_1.fastq.gz
Parkin_IR_1_H3K27ac_T1_2.fastq.gz
Parkin_IR_1_H3_T1_1.fastq.gz
Parkin_IR_1_H3_T1_2.fastq.gz
Parkin_IR_3_H3K27ac_T1_1.fastq.gz
Parkin_IR_3_H3K27ac_T1_2.fastq.gz
Parkin_IR_3_H3_T1_1.fastq.gz
Parkin_IR_3_H3_T1_2.fastq.gz
Parkin_IR_4_H3K27ac_T1_1.fastq.gz
Parkin_IR_4_H3K27ac_T1_2.fastq.gz
Parkin_IR_4_H3_T1_1.fastq.gz
Parkin_IR_4_H3_T1_2.fastq.gz
Parkin_Prolif_1_H3K27ac_T1_1.fastq.gz
Parkin_Prolif_1_H3K27ac_T1_2.fastq.gz
Parkin_Prolif_1_H3_T1_1.fastq.gz
Parkin_Prolif_1_H3_T1_2.fastq.gz
Parkin_Prolif_2_H3K27ac_T1_1.fastq.gz
Parkin_Prolif_2_H3K27ac_T1_2.fastq.gz
Parkin_Prolif_2_H3_T1_1.fastq.gz
Parkin_Prolif_2_H3_T1_2.fastq.gz
Parkin_Prolif_3_H3K27ac_T1_1.fastq.gz

Parkin_Prolif_3_H3K27ac_T1_2.fastq.gz
Parkin_Prolif_3_H3_T1_1.fastq.gz
Parkin_Prolif_3_H3_T1_2.fastq.gz

Genome browser session
(e.g. UCSC)

https://genome.ucsc.edu/cgi-bin/hgTracks?db=hg38&lastVirtModeType=default&lastVirtModeExtraState=&virtModeType=default&virtMode=0&nonVirtPosition=&position=chr7%3A155799529%2D155812871&hgsid=2362406411_jIJjMEkbDlUc7c9CnYGYSsZauWA1

Methodology

Replicates

For Parkin and CTPI2 Each CHIP-seq have been run in 3 independent replicates for each conditions and each histone mark
For Bax/Bak average of two independent replicate for H3K27ac and average of 3 replicates for the H3 for normalization

Sequencing depth

10M read in average

Antibodies

Histone H3 Abcam ab1791
H3K27ac Abcam ab4729

Peak calling parameters

N/A

Data quality

Run with nf-core/chipseq analysis pipeline MultiQC

Software

ANNOTATE_BOOLEAN_PEAKS sed 4.7
BAM_FILTER bamtools 2.5.2
samtools 1.15.1
BEDTOOLS_GENOMECOV bedtools 2.30.0
BOWTIE2_ALIGN bowtie2 2.4.4
pigz 2.6
samtools 1.15.1
CUSTOM_DUMPSOFTWAREVERSIONS python 3.10.5
yaml 6.0
CUSTOM_GETCHROMSIZES custom 1.15.1
DEEPTOOLS_COMPUTEMATRIX deeptools 3.5.1
DEEPTOOLS_PLOTFINGERPRINT deeptools 3.5.1
DEEPTOOLS_PLOTHEATMAP deeptools 3.5.1
DEEPTOOLS_PLOTPROFILE deeptools 3.5.1
FASTQC fastqc 0.11.9
FRIP_SCORE bedtools 2.30.0
samtools 1.15.1
GENOME_BLACKLIST_REGIONS bedtools 2.30.0
GTF2BED perl 5.26.2
HOMER_ANNOTATEPEAKS_CONSENSUS homer 4.11
HOMER_ANNOTATEPEAKS_MACS2 homer 4.11
IGV python 3.8.3
MACS2_CALLPEAK macs2 2.2.7.1
MACS2_CONSENSUS python 3.10.0
r-base 4.1.1
PHANTOMPEAKQUALTOOLS phantompeakqualtools 1.2.2
PICARD_COLLECTMULTIPLEMETRICS picard 2.27.4-SNAPSHOT
PICARD_MARKDUPLICATES picard 2.27.4-SNAPSHOT
PICARD_MERGESAMFILES picard 2.27.4-SNAPSHOT
PLOT_HOMER_ANNOTATEPEAKS r-base 4.0.3
PLOT_MACS2_QC r-base 4.0.3
PRESEQ_LCEXTRAP preseq 3.1.1
SAMPLESHEET_CHECK python 3.8.3
SAMTOOLS_FLAGSTAT samtools 1.15.1
SAMTOOLS_IDXSTATS samtools 1.15.1
SAMTOOLS_INDEX samtools 1.15.1
SAMTOOLS_STATS samtools 1.15.1
SUBREAD_FEATURECOUNTS subread 2.0.1
TRIMGALORE cutadapt 3.4
trimgalore 0.6.7
UCSC_BEDGRAPHTOBIGWIG ucsc 377
Workflow Nextflow 23.10.0
nf-core/chipseq 2.0.0

Final analysis with Galaxy website and deeptools
