# Supplementary material for: Mitochondrial metabolism and epigenetic crosstalk drive SASP
Source: Nature. 2026 Jul 29;656(8129):980–92. doi: 10.1038/s41586-026-10791-2 (PMC13518223; doi:10.1038/s41586-026-10791-2)
Supplement: Supplementary file 3 [file 41586_2026_10791_MOESM3_ESM.pdf]

nature portfolio

Peer Review File

Mitochondrial metabolism and epigenetic crosstalk drive the SASP

Corresponding Author: Professor Joao Passos

Any redactions in this file are there to maintain patient confidentiality, the confidentiality of unpublished data, or to remove third-party material.

This file contains all reviewer reports in order by version, followed by all author rebuttals in order by version.

Version 0:

Reviewer comments:

Referee #1

(Remarks to the Author)
In this paper Martini and colleagues describe interesting findings on the role of the mitochondrial citrate carrier and ATP-citrate lyase in regulating the SASP phenotype. The key process here is histone acetylation on SASP gene loci. Importantly, in vivo data are also presented which indicate that inhibiting SLC25A1 has an anti-inflammatory effect in vivo and also promotes healthspan in mice based on various metrics. Overall these results are interesting and potentially important. I have however a number of issues that need to be addressed to strengthen the conclusions being drawn.

1. The authors deploy a technique to deplete mitochondria using Parkin and CCCP. They have reported on this previously but how can they be sure there aren't non-specific effects happening here that impact on the SASP? The main control presented is for GAPDH expression as shown in FIg 1b. This seems inadequate. What about cell viability, respiration being impaired, rate of glycolysis? Some more controls need to be shown here.

2. Figure 2 shows how acetate supplementation can partially restore the expression of SASP components. Why is this effect only partial if acetyl-CoA is so central here? The authors should measure levels of acetyl-CoA in response to the acetate as it might be limiting here.

3. Similarly we have data on SLC25A1 knockout cells and also the small molecule CTPI1, which is a key reagent here. I think it is important to measure cytosolic citrate and acetyl-CoA in these experiments to ensure it's levels are being decreased using these approaches, otherwise we are relying on the specificity of the knockout and small molecule here.

4. I must say that I find the effect of CTPI2 in vivo somewhat surprising in it's specificity. U suspect that it was these remarkable in vivo effects that prompted the authors to submit this work to Nature, any they are indeed impressive. However we need a lot more data to be sure that it's effect is due to inhibition of histone acetylation on SASP loci. First, an inhibition of that process needs to be shown in samples taken from the mice, possibly muscle or other tissue. Secondly surely inhibiting SLC25A1 will have all kinds of metabolic consequences here? What about fatty acid synthesis? Is that being impaired and might that in part explain the in vivo effects here? What are the treated mice like metabolically, eg in terms of insulin resistence or other metabolic parameters. We need more evidence that CTPI2 is working in vivo in the way the authors claim.

Referee #2

(Remarks to the Author)
The manuscript from Martini et al. investigates how ‘mitochondrial metabolism and epigenetic crosstalk drive the SASP’. Inflammation as part of the SASP is a key mediator of the damage caused by senescent cells. Research investigating how the SASP is regulated is of potential relevance as a way to target senescent cells in ageing. There are numerous examples of mechanisms that regulate and can be used to target the SASP, including those involving metabolism, mitochondria, and

epigenetic regulation. Some of that preexisting research has been authored by the Passos and Adams labs (e.g. Dasgupta et al., NPJ Aging 2024; Dasgupta et al., Mol Cell Biol 2024; Victorelli et al., Nature 2023; Vizioli, Genes Dev 2020, etc.). Others have also connected the SASP with metabolism (e.g. Laberge et al., Nat Cell Biol 2016; Herranz et al., Nat Cell Biol 2016) or epigenetic control (Tasdemir et al., Cancer Discovery 2016) before. Therefore, the manuscript might be more suitable for publication in a more specialised journal such as Nat Metabolism, Nat Aging or Nat Cell Biol. Comments:
1. Many of the observed effects that are inferred as causal are likely correlation: the increase or decrease in H3K27 acetylation levels observed in SASP genes correlates with the expression/repression of SASP genes, and a similar relation might be observed if the SASP is inhibited by other means.
2. The changes in H3K27 acetylation are suggested to be linked to changes in metabolites. No explanation is given on whether there is any specificity to H3K27 acetylation or whether similar effects are observed in other acetylation marks or protein acetylation.
3. Activation of cGAS/STING is a key element to induce the SASP. No data is presented on whether the activation is decreased or not or why.
4. The authors have previously described many alterations in mitochondria, including the release of mtDNA that affect the SASP, but how or whether those are affected has not been studied.

Referee #3

(Remarks to the Author)
The manuscript by Martini et al describes a series of experiments to link acetyl-CoA production to the expression of genes often associated with senescence-associated secretory phenotype (SASP). The approaches included removal of mitochondria, inhibition of the citrate transporter and pyruvate carrier, as well as gene knockouts (like ACLY, ATP-citrate lyase). Not surprisingly, when the authors manipulated the ability of the cells to produce acetyl-CoA there was a corresponding decrease in chromatin acetylation and gene expression. The authors focused on the expression of SASP genes, which as reported previously, are controlled by histone acetylation, along with most responsive genes. The authors note that inhibiting these metabolic pathways generating acetylCoA does not affect cell cycle arrest, which is classically associated with senescence and SASP. Moving to a mouse model, the authors show that SLC25A1 inhibition reduces inflammation and extends healthspan in aged mice. Overall, the study appears to be carefully performed and interpreted. Also, the mouse studies showing that inhibition of the citrate transporter improved inflammatory endpoints and other age-associated physiological changes is an important finding. However, two relatively significant weaknesses dampen enthusiasm. Histone acetylation and increased gene expression (of SASP and other genes) being dependent on production of acetyl-CoA is well established. The second is the lack mechanistic insight on why/how SASP genes would be preferentially targeted, if they are at all.

Overall highlights
The authors careful use of a host of reagents and methods to demonstrate the role of acetyl-CoA in gene expression.
The use of acetate to partially rescue the phenotype is interesting and was not entirely expected, as ACSS2 would have to be expressed at sufficient amounts, perhaps induced after an acetyl-CoA deficit.
The in vivo studies are promising.
The lack of cell cycle effect may suggest that the gene expression changes are not really indicative of SASP, but a general response to acetyl-CoA availability.
Questions/Concerns
The fact that acetyl-CoA metabolism is connected to histone acetylation and gene expression is well-established. What makes this specific for SASP genes? If it truly is, then what is the mechanism? Do ACLY and ACSS2 sit at the enhancer/promoters of only SASP genes and directs p300 K27 acetylation? Why only those loci?
If other the classic markers of senescence are not changing, then is this really SASP. The authors narrowly focus on SASP genes, but what is the direct evidence that this is not just a global metabolic stress response?
What is the direct evidence of actual secretion of factors in cell culture media and mouse serum?

Version 1:

Reviewer comments:

Referee #1

(Remarks to the Author)
The authors have adequately addressed my concerns.

Referee #2

(Remarks to the Author)
The authors have addressed most of the reviewers' comments, and the story is compelling. The question remains whether what they are describing (mitochondrial and epigenetic control of the SASP) is considered novel to grant publication in Nature in view of the existing literature, but that is an editorial call!

Open Access This Peer Review File is licensed under a Creative Commons Attribution 4.0 International License, which permits use, sharing, adaptation, distribution and reproduction in any medium or format, as long as you give appropriate credit to the original author(s) and the source, provide a link to the Creative Commons license, and indicate if changes were made.
In cases where reviewers are anonymous, credit should be given to 'Anonymous Referee' and the source.
The images or other third party material in this Peer Review File are included in the article's Creative Commons license, unless indicated otherwise in a credit line to the material. If material is not included in the article's Creative Commons license and your intended use is not permitted by statutory regulation or exceeds the permitted use, you will need to obtain permission directly from the copyright holder.
To view a copy of this license, visit https://creativecommons.org/licenses/by/4.0/

Point by point response to referees.

We thank all referees for their constructive comments, which have helped improve both the quality and conceptual clarity of our manuscript.

Referee #1 (Remarks to the Author):

The authors deploy a technique to deplete mitochondria using Parkin and CCCP. They have reported on this previously but how can they be sure there aren't non-specific effects happening here that impact on the SASP? The main control presented is for GAPDH expression as shown in FIg 1b. This seems inadequate. What about cell viability, respiration being impaired, rate of glycolysis? Some more controls need to be shown here.

We appreciate this concern and have added controls to address potential non-specific effects of mitochondrial depletion. Mitochondrial depletion did not affect viability. Cell numbers remained stable by direct counting (Extended Data Fig. 1a), and LDH release showed no increase in cytotoxicity (Extended Data Fig. 1b). We confirmed mitochondrial depletion. Mitochondrial proteins are lost, and respiration is abolished. Parkin-expressing senescent cells treated with CCCP show no mitochondrial respiration and are insensitive to mitochondrial inhibitors (Extended Data Fig. 1c). We agree that metabolic compensation is an important consideration. We have previously shown in this model that ATP levels are maintained following mitochondrial depletion due to compensatory glycolysis (PMID: 26848154). We have replaced GAPDH with Actin as a loading control in Figure 1b.

We fully agree that this model is non-physiological and may have pleiotropic effects. We therefore use this system primarily as an initial discovery tool rather than as sole evidence for mechanism. For this reason, all key conclusions were validated using complementary approaches targeting mitochondrial citrate metabolism without removing mitochondria, including inhibition of the citrate transporter, inhibition of the mitochondrial pyruvate carrier, and genetic perturbation of ACLY.

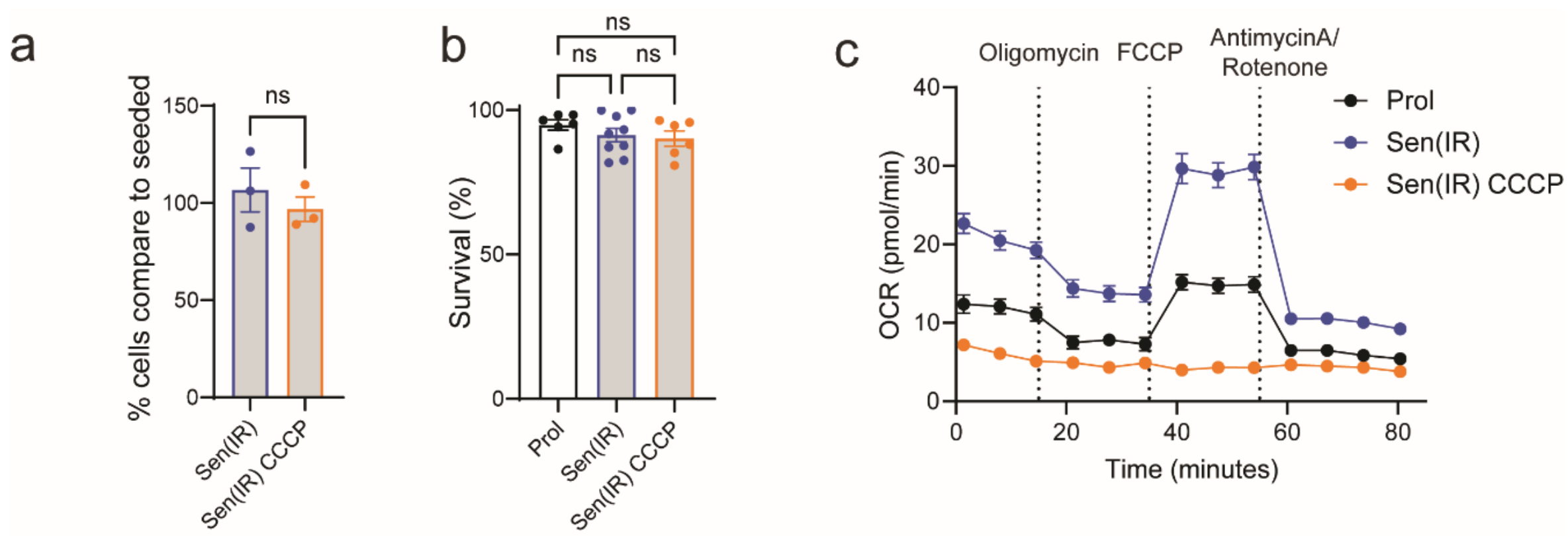

Extended Data Figure 1:
(a) Percentage of cells D12 post irradiation compared to the number of cells seeded at D0 (n=3). **(b)** % of survival cells assess by LDH assay (n=6-9). **(c)** Seahorse analysis showing oxygen consumption rate (OCR) profiles (n=4-14).

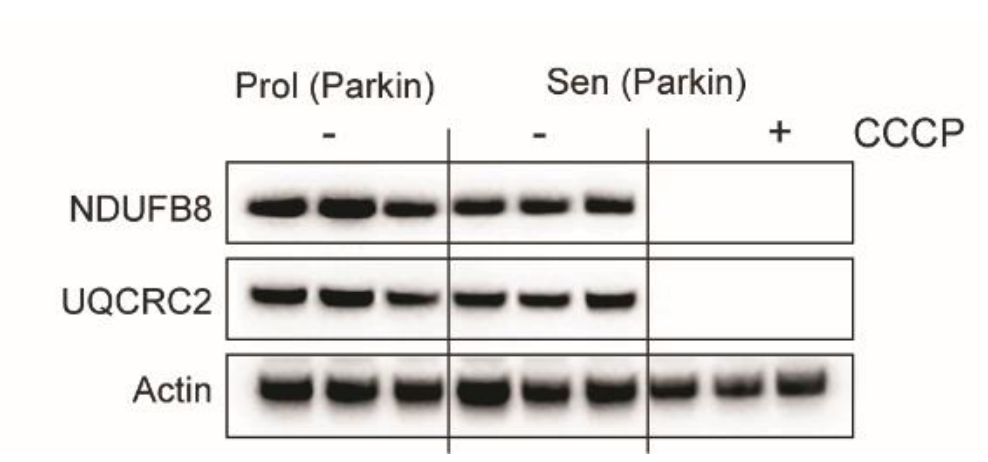

Figure 1b:
(b) Western blot showing mitochondrial proteins UQCRC2 and NDUFB8, confirming loss of mitochondria in IMR90 Parkin senescent cells following CCCP treatment (Sen+CCCP).

Figure 2 shows how acetate supplementation can partially restore the expression of SASP components. Why is this effect only partial if acetyl-CoA is so central here? The authors should measure levels of acetyl-CoA in response to the acetate as it might be limiting here.

We thank the reviewer for this insightful comment. In response, we performed a more comprehensive analysis of acetate supplementation in mitochondria-depleted senescent cells, including RNA-seq (revised Fig. 1i). These experiments show that acetate restores only a partial SASP program: a subset of SASP genes is induced, but their expression does not reach the levels observed in senescent control cells with intact mitochondria.

We considered whether this incomplete rescue reflects insufficient restoration of acetyl-CoA. Several observations argue against acetate availability being limiting. In proliferating cells of the same type, ^{13}C-acetate supplementation produced a rapid and robust increase in acetyl-CoA, demonstrating efficient conversion of exogenous acetate (Extended Data Fig.3b). Acetate treatment also strongly increased H3K27ac and nuclear acetyl-lysine signals (Extended Data Fig.3c-e). In mitochondria-depleted senescent cells, acetate similarly increased histone acetylation and nuclear acetyl-lysine (Fig.1g,h), indicating that acetate remains available for acetylation reactions in this context.

These findings suggest that acetate alone may be insufficient because mitochondria provide an additional signal required for full SASP activation. Parkin-mediated mitophagy removes not only mitochondrial metabolic activity but also cytosolic mitochondrial DNA (mtDNA), which we previously showed activates the SASP via cGAS-STING signaling (PMID: 37821702).

New experiments support a model in which full SASP induction requires convergence of two mitochondrial outputs: (i) mtDNA-driven innate immune signaling and (ii) acetyl-CoA-dependent histone acetylation. Acetate restores chromatin acetylation but does not fully restore SASP transcription in the absence of mtDNA signaling, whereas combined restoration of acetate-dependent acetylation and mtDNA signaling produces a stronger rescue (see new Fig. 5 and Extended Data Fig. 9, see response to reviewer 2). Thus, we suggest that the partial effect of acetate reflects the requirement for complementary mitochondrial inputs rather than insufficient acetate utilization.

Fig.1i

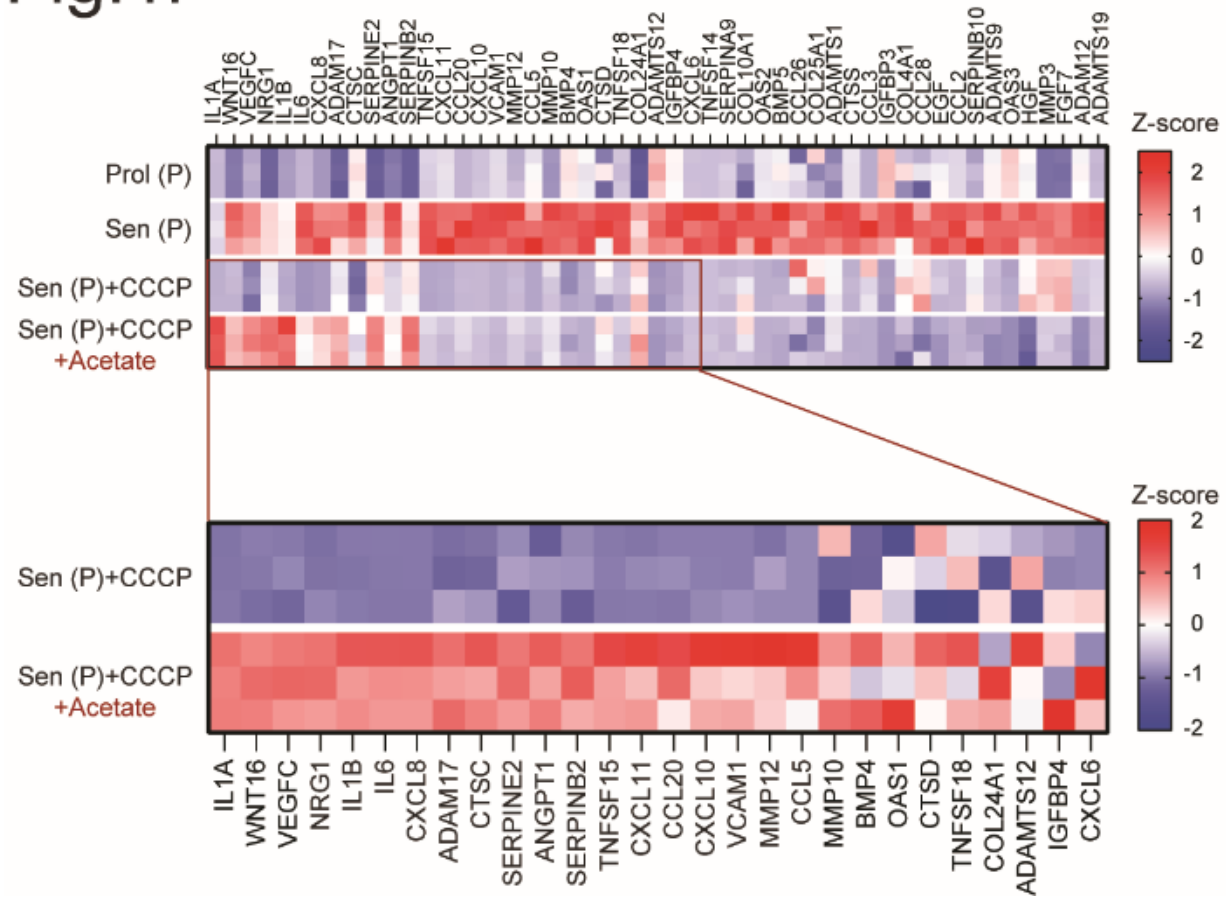

Extended Data Fig.3b

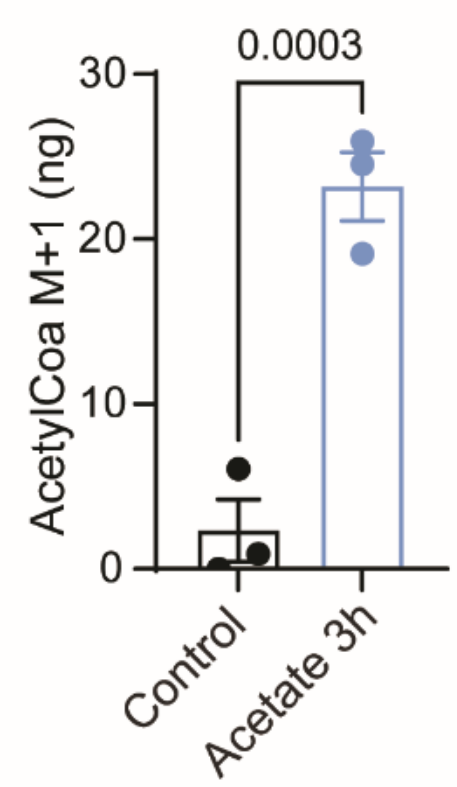

Figure 1i: Upper panel : Heatmap of SASP genes in senescent cells (Sen(P)), senescent cells in absence of mitochondria (Sen(P) + CCCP) and after Acetate treatment (Sen(P) + CCCP + Acetate). Lower Panel: Heatmap of the SASP genes which show increase expression in cells without mitochondria after Acetate treatment. The color intensity represents column Z-score.

Extended Data Fig.3b: Incorporation of ^{13}C-labeled acetate into acetyl-CoA (M+1) measured 3 h after supplementation (n=3).

Similarly we have data on SLC25A1 knockout cells and also the small molecule CTPI1, which is a key reagent here. I think it is important to measure cytosolic citrate and acetyl-CoA in these experiments to ensure it's levels are being decreased using these approaches, otherwise we are relying on the specificity of the knockout and small molecule here.

We thank the reviewer for this important suggestion. To directly assess whether SCL25A1 inhibition reduce cytosolic citrate metabolism and acetyl-CoA availability, we performed targeted metabolomic analyses following

CTPI2 treatment. Metabolomic profiling revealed that CTPI2 selectively decreased tricarboxylic acid (TCA) cycle intermediates linked to citrate metabolism, including citrate, cis-aconitate, and isocitrate (Extended Data Fig. 8j).

To determine compartment-specific effects, we conducted subcellular fractionation coupled to metabolite quantification. These experiments indicate that CTPI2 reduced citrate and acetyl-CoA abundance in the cytosolic compartment (Extended Data Fig. 8k-l). This is consistent with inhibition of citrate export and reduced substrate availability for ATP-citrate lyase-dependent acetyl-CoA production, thereby limiting histone acetylation.

Extended Data Figure 8:

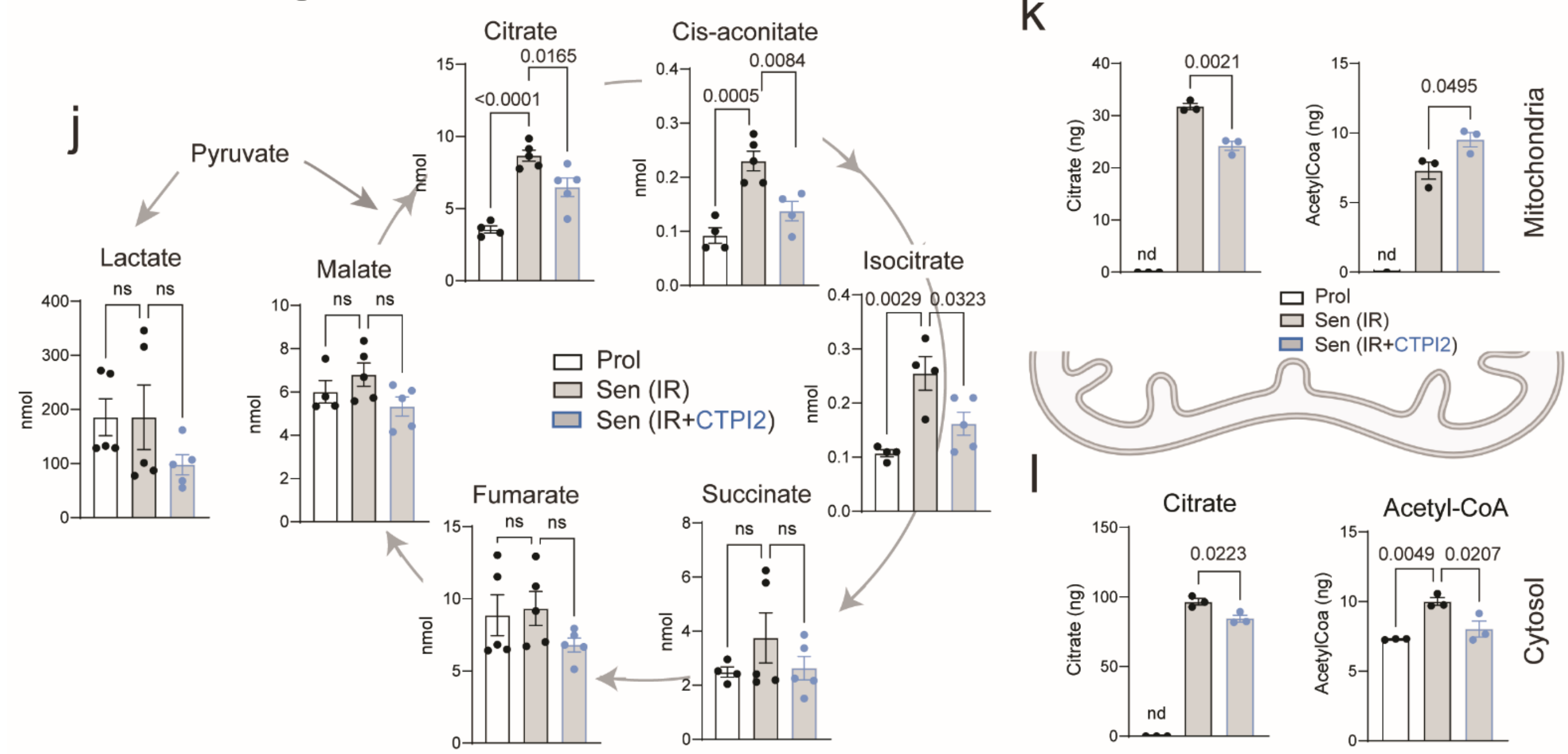

Extended Data Figure 8: **(j)** TCA cycle metabolite levels measured in proliferative and senescent cells with or without CTPI2 treatment, expressed in nmol per 5 million cells (n=4-5). **(k-l)** Citrate and Acetyl-CoA measurement in the mitochondrial (k) or cytosolic (l) fraction in proliferative and senescent cells treated or not by CTPI2 (n=3). Data are shown as Mean ± s.e.m. Statistical tests: One-way ANOVA with Tukey's multiple comparison test (j,l) or Student's t-test (k,l). (n= independent experiment)

I must say that I find the effect of CTPI2 in vivo somewhat surprising in it's specificity. U suspect that it was these remarkable in vivo effects that prompted the authors to submit this work to Nature, any they are indeed impressive. However we need a lot more data to be sure that it's effect is due to inhibition of histone acetylation on SASP loci. First, an inhibition of that process needs to be shown in samples taken from the mice, possibly muscle or other tissue. Secondly surely inhibiting SLC25A1 will have all kinds of metabolic consequences here? What about fatty acid synthesis? Is that being impaired and might that in part explain the in vivo effects here? What are the treated mice like metabolically, eg in terms of insulin resistence or other metabolic parameters. We need more evidence that CTPI2 is working in vivo in the way the authors claim.

We thank the reviewer for this thoughtful critique and for considering the *in vivo* effects of CTPI2 to be impressive. To address these points, we performed additional in vivo experiments, including single-nucleus multiome profiling, analysis of nuclear acetylation using 4i, and comprehensive metabolic phenotyping.

To determine whether CTPI2 alters transcriptional output and chromatin accessibility in vivo, we performed single-nucleus multiome (RNA + ATAC) profiling of quadriceps muscle from aged treated mice (new Fig. 7 and Extended Data Fig. 13). We focused on skeletal muscle, where SLC25A1 inhibition improved neuromuscular coordination and balance (pole test), endurance (hanging test), and forelimb strength (Fig. 6e-g).

Single-nucleus multiome analysis, which profiles gene expression and chromatin accessibility in the same cells, showed that senescent-like nuclei exhibit increased expression of SASP genes together with increased

chromatin accessibility at these loci. Following CTPI2 treatment, both SASP expression and chromatin accessibility were reduced within this population, while the overall abundance of senescent-like nuclei was unchanged.

To further evaluate nuclear acetylation *in vivo*, we analyzed liver tissue using iterative indirect immunofluorescence (4i) (Fig.7l-q). Senescent cells ($p16^+$ and/or $p21^+/PCNA^-$) were present at similar levels in treated and control animals. These cells showed higher nuclear pan-acetyl-lysine signal than non-senescent cells, which was reduced following CTPI2 treatment.

Finally, we assessed whether SLC25A1 inhibition induces broader metabolic effects. Comprehensive metabolic phenotyping showed that CTPI2-treated aged mice maintained glucose tolerance comparable to controls, with modest reductions in fasting and fed glucose levels (Extended Data Fig. 15a-c). Body weight, body composition, energy expenditure, respiratory exchange ratio, and activity were unchanged (Extended Data Fig. 15e-l). Lipid and fatty-acid metabolic pathways in liver, muscle, and heart were also not altered (Extended Data Fig. 15m-s), consistent with results in senescent fibroblasts in vitro (Extended Data Fig. 15t,u).

The *in vivo* intervention was repeated in an independent cohort of aged mice with similar functional outcomes.

Figure 6

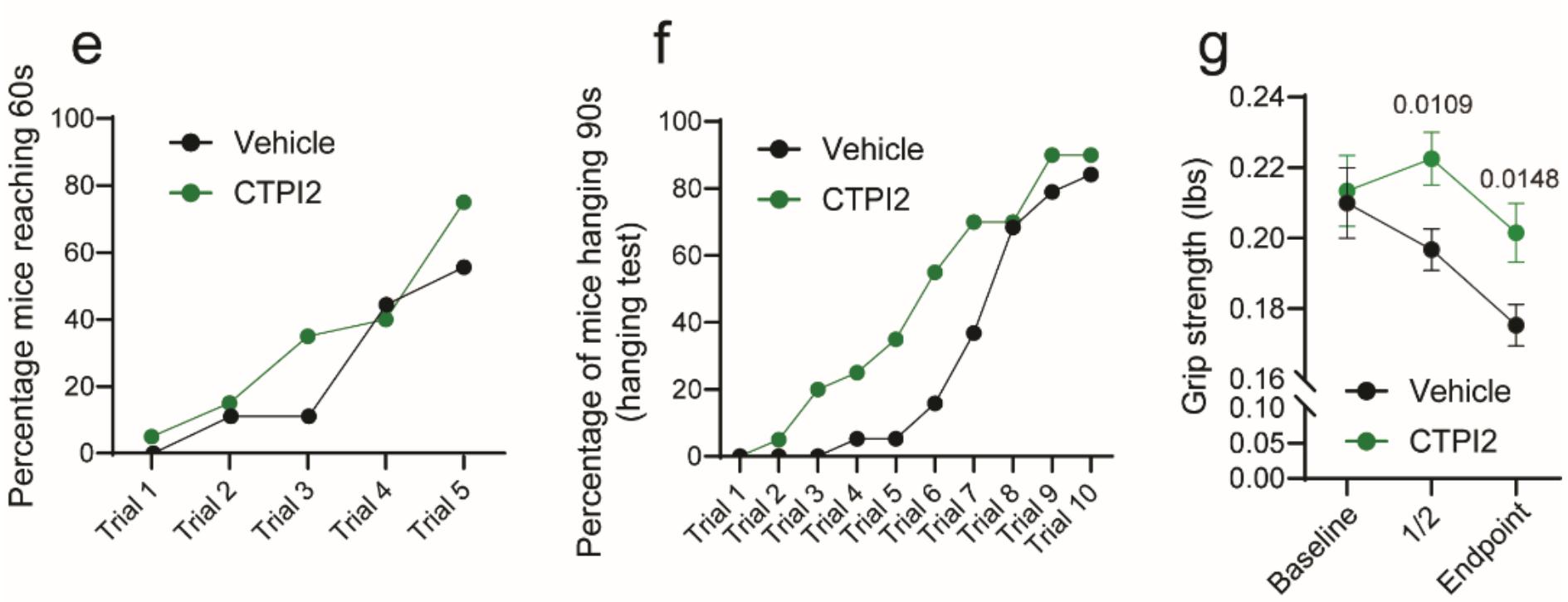

Figure 6: (e) Cumulative percentage of mice succeeding in tightrope test by staying on the pole for 60s over the 5 trials (n=18 vehicle, n=20 CTPI2 treated mice, male and females combined). **(f)** Cumulative percentage of mice succeeding in the hanging test by hanging 90s over the 10 trials (n=19 vehicle, n=20 CTPI2-treated mice, male and female combine) **(h)** Forelimb grip strength measured at baseline, 1.5 months, and 3 months (final) in each group (n=19 per group).

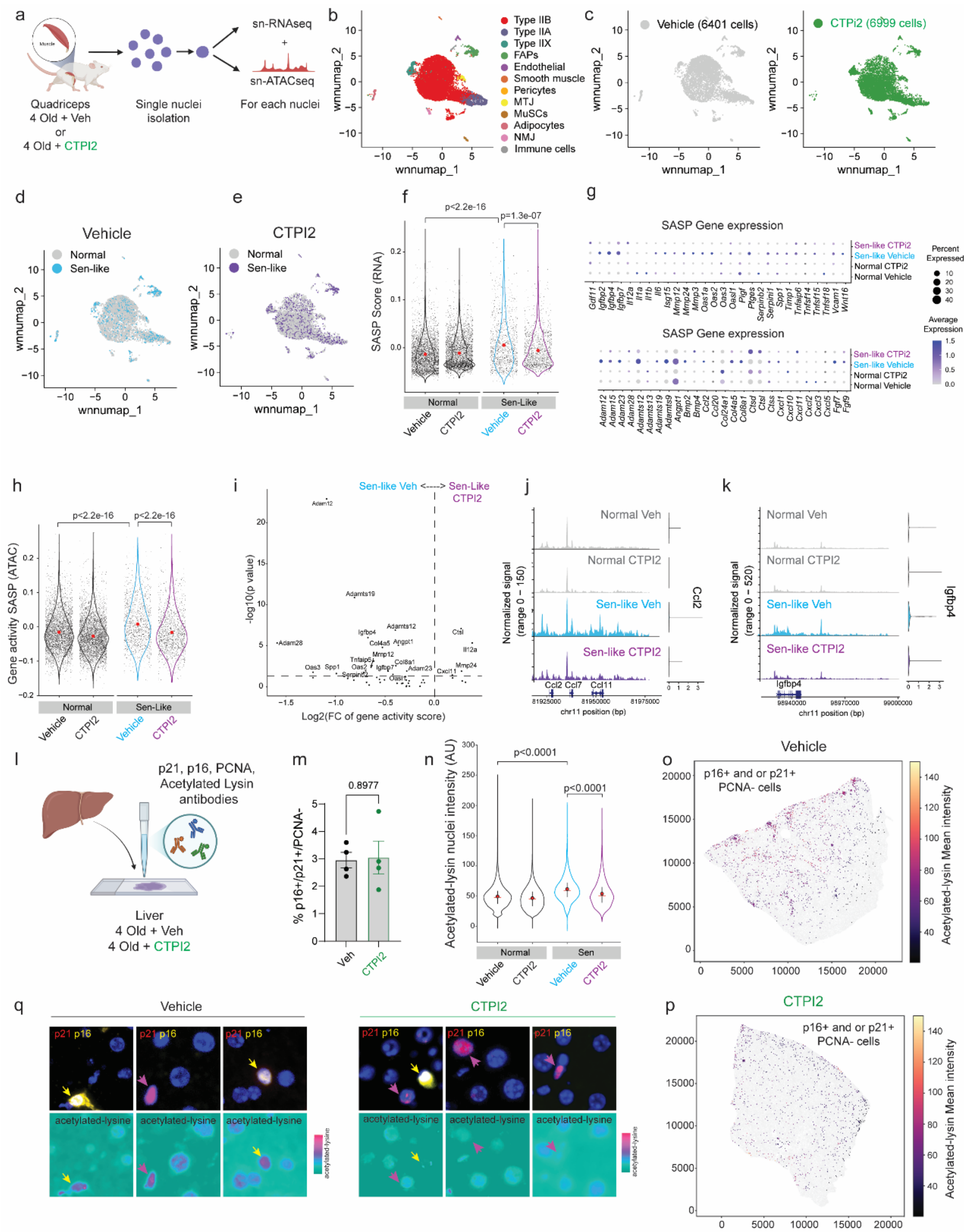

Figure 7: Pharmacological inhibition of SLC25A1 suppresses SASP programs and chromatin accessibility in senescent cells *in vivo*.

(a) Schematic of the multiome experimental workflow. **(b)** Weighted nearest neighbor (WNN) UMAP showing cell-type clusters. **(c)** WNN UMAP illustrating the number and distribution of cells from Vehicle and CTPI2-treated samples. **(d-e)** WNN UMAP showing the distribution of senescent-like cells across clusters in Vehicle (d) and CTPI2-treated (e) samples. **(f)** Violin plot of per-nucleus SASP score based on RNA expression. **(g)** Dot plot showing mean RNA expression and the proportion of cells expressing each SASP factor across the four conditions. **(h)** Violin plot of per-nucleus gene activity scores for SASP factors derived from ATAC-seq, reflecting chromatin accessibility. **(i)** Volcano plot comparing gene activity scores of individual SASP genes in senescent-like cells from Vehicle versus CTPI2-treated samples. **(j-k)** Coverage plots for representative SASP genes *Ccl2* (j) and *Igfbp4* (k). **(l)** Schematic of the experimental workflow for histological analyses. **(m)** Percentage of cells classified as p16^{+} and/or p21^{+} and PCNA^{-} (non-proliferative) per sample. **(n)** Nuclear acetyl-lysine intensity in p16^{+}/p21^{+}/PCNA^{-} cells (senescent) and normal cells in Vehicle- and CTPI2-treated groups. **(o-p)** Representative spatial plots of liver sections from Vehicle (o) and CTPI2-treated (p) mice showing the distribution of p16^{+}/p21^{+}/PCNA^{-} cells and their nuclear acetyl-lysine intensity. **(q)** Representative immunofluorescence images of p21, p16, and acetyl-lysine staining in Vehicle and CTPI2-treated livers. Data in (d, g, i) are shown as mean values (red dots). Statistical analyses were performed using the Wilcoxon test (d, g, i), one-way ANOVA with Beferroni's multiple comparison test (n), or Student's *t*-test (m).

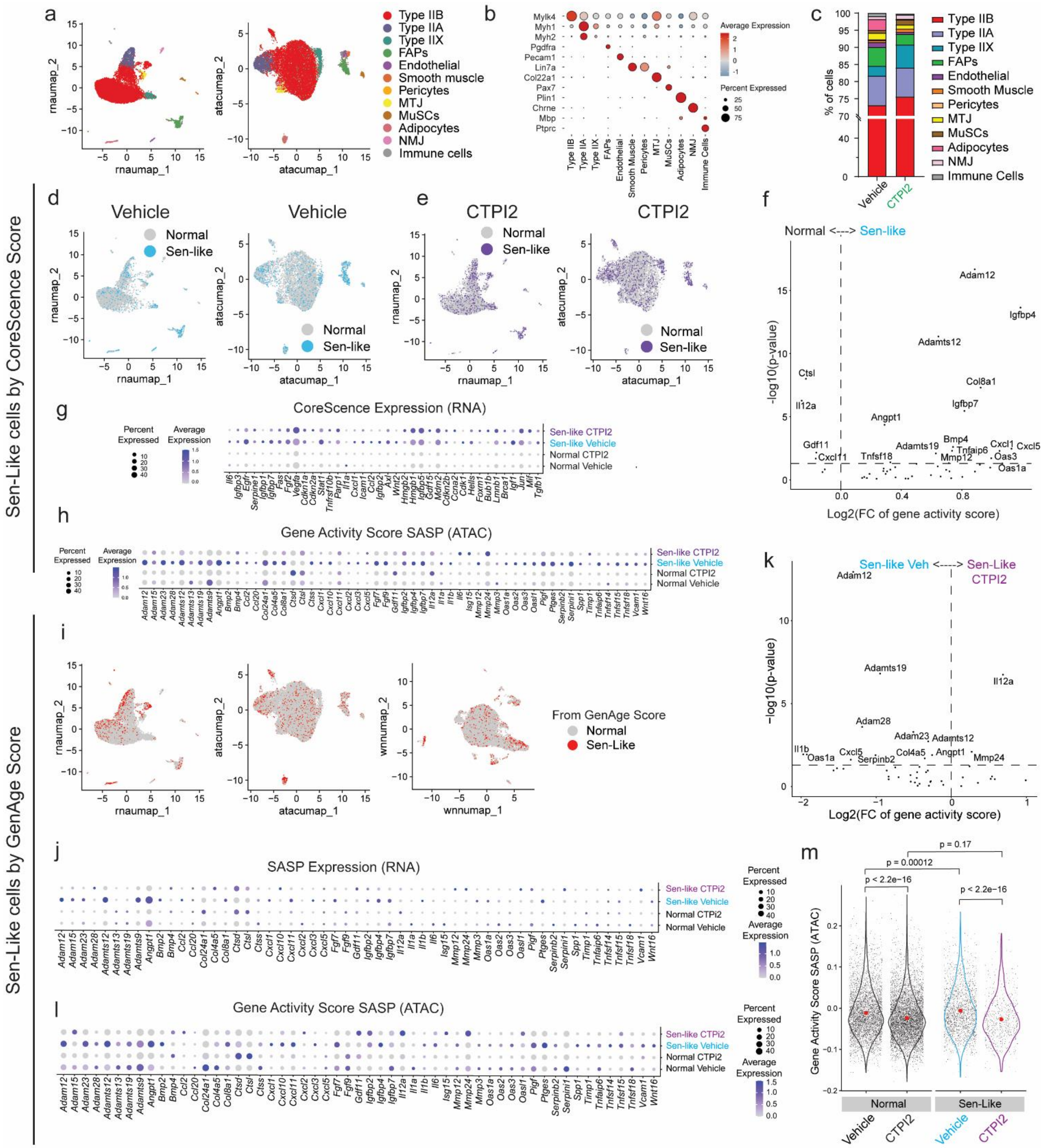

Extended Data Figure 13: Pharmacological inhibition of SLC25A1 suppresses SASP programs and chromatin accessibility in senescent cells *in vivo*.

(a) UMAPs representing the different cell type clusters from sn-RNA data set (left panel) or sn-ATAC-seq (right panel) **(b)** Dot plot showing the expereession of the differents markers used for cell type identification **(c)** Histogram showing the cell type repartition between Vehicle or CTPI2 sample. **(d-e)** UMAPs showing the repartition of Sen-like cells in Vehicle **(d)** or CTPI2 **(e)** samples from sn-RNA data set (left panel) or sn-ATAC-seq (right panel). **(f)** Violin plot of gene activity of the SASP genes in normal vs Sen-like cells in the vehicle group. **(g)** Dot plot of average expression and abundance of the different CoreScence genes between normal and Sen-like cells in the samples treated or not by CTPI2. **(h)** Dot plot of average gene activity and abundance of different SASP genes. **(i)** UMAPs abundance and repartition of senescent cells likes identified by high GeneAge Score. **(j-l)** Dot plot of average and abundance of gene expression **(j)** or gene activity **(l)** of SASP genes in Sen-like cells identified by GeneAge Score. **(k)** Violin plot of the individual nuclei Score for GeneAge expereession showing the selection of Sen-like vs normal cells. **(m)** Violin plot of individual nuclei gene activity of SASP genes. Data are represented as Mean (red dots). Statistical analysis was performed using Wilcoxon test.

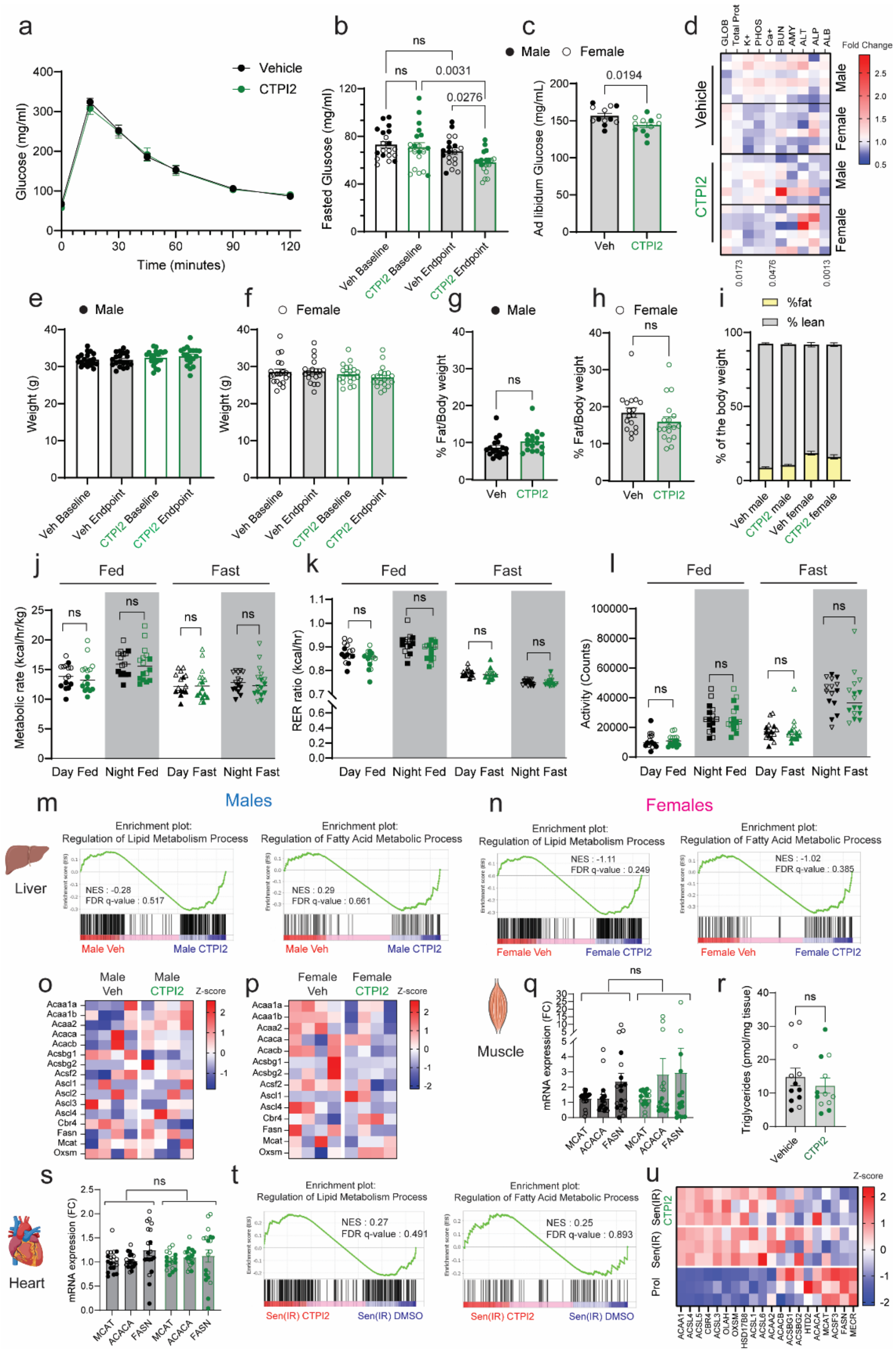

Extended Data Figure 15: Pharmacological inhibition of the mitochondrial citrate carrier (SLC25A1) does not induce systemic metabolic reprogramming in mice. (a) Intraperitoneal glucose tolerance test (IP-GTT) showing blood glucose levels at baseline (0 min) and 15, 30, 45, 60, 90, and 120 minutes after glucose injection in Vehicle- and CTPI2-treated mice (Vehicle *n=19*; CTPI2 *n*=20; males and females combined). **(b)** Fasting blood glucose levels at baseline and after 3 months of Vehicle or CTPI2 treatment. **(c)** Ad libitum blood glucose levels (*n*=6 per group per sex). **(d)** Plasma chemistry parameters in Vehicle- or CTPI2-treated mice (*n*=6 per group per sex). **(e–f)** Body weight of male (e) and female (f) mice at baseline and after 3 months of treatment.**(g-h)** Fat mass normalized to body weight in male (g) and female (h) mice. **(i)** Whole-body composition normalized to body weight. **(j-l)** Comprehensive Lab Animal Monitoring System (CLAMS) measurements, including metabolic rate (j), respiratory exchange ratio (RER) (k), and locomotor activity (l), across light/dark cycles under fed and fasting conditions (*n* = 8 per sex per group). **(m-n)** Gene set enrichment analysis (GSEA) plots comparing liver transcriptomes of Vehicle- and CTPI2-treated males (m) and females (n). **(o-p)** RNA-seq heatmaps of key lipid metabolism genes in male (o) and female (p) livers (Z-score representation; *n=4* per condition). **(q)** Relative mRNA expression of key lipid metabolism enzymes in quadriceps muscle from male and female mice treated with CTPI2 or Vehicle, expressed as fold change relative to Vehicle controls. **(r)** Triglyceride content in quadriceps muscle from Vehicle- and CTPI2-treated mice. **(s)** Relative mRNA expression of lipid metabolism enzymes in heart tissue from male and female mice treated with CTPI2 or Vehicle, expressed as fold change relative to Vehicle controls. **(t)** GSEA plot comparing proliferative versus senescent cells with or without CTPI2 treatment. **(u)** RNA-seq heatmap of lipid metabolism genes in proliferative and senescent cells ± CTPI2 (Z-score representation; *n=3*). Data are presented as mean ± s.e.m. Statistical analyses were performed using one-way ANOVA with Tukey's multiple comparison test (b) or Student's *t*-test. Male data points are shown as filled symbols and female data points as open symbols.

Referee #2 (Remarks to the Author):

The manuscript from Martini et al. investigates how 'mitochondrial metabolism and epigenetic crosstalk drive the SASP'. Inflammation as part of the SASP is a key mediator of the damage caused by senescent cells. Research investigating how the SASP is regulated is of potential relevance as a way to target senescent cells in ageing. There are numerous examples of mechanisms that regulate and can be used to target the SASP, including those involving metabolism, mitochondria, and epigenetic regulation. Some of that preexisting research has been authored by the Passos and Adams labs (e.g. Dasgupta et al., NPJ Aging 2024; Dasgupta et al., Mol Cell Biol 2024; Victorelli et al., Nature 2023; Vizioli, Genes Dev 2020, etc.). Others have also connected the SASP with metabolism (e.g. Laberge et al., Nat Cell Biol 2016; Herranz et al., Nat Cell Biol 2016) or epigenetic control (Tasdemir et al., Cancer Discovery 2016) before. Therefore, the manuscript might be more suitable for publication in a more specialised journal such as Nat Metabolism, Nat Aging or Nat Cell Biol.

We thank the reviewer for this important point and agree that multiple studies, including work from our laboratories and others, have identified metabolic, mitochondrial, and chromatin-based mechanisms that influence the SASP.

However, our study addresses a different question. Rather than identifying an additional regulator of the SASP, we examine how mitochondrial outputs contribute to the organization and regulation of SASP-associated chromatin states.

Specifically, our data show that:

1-Mitochondria-dependent, acetylation-associated chromatin activity is enriched at SASP-associated loci. Mitochondria-dependent chromatin accessibility and transcriptional changes in senescent cells are enriched at loci associated with the SASP. These findings suggest that mitochondrial control of acetyl-CoA availability preferentially supports acetylation-dependent chromatin activity at SASP genes.

2. Mitochondrial citrate-derived acetyl-CoA is required to sustain the SASP. We show that the mitochondrial pyruvate–citrate–acetyl-CoA pathway is upregulated in senescent cells, providing a critical source of cytosolic acetyl-CoA to support histone acetylation and transcriptional activation at SASP loci. Perturbation of this pathway, through inhibition of mitochondrial citrate export, suppresses SASP gene expression without reversing the senescent state, indicating that acetyl-CoA availability is a limiting factor for SASP maintenance.

3. mtDNA signaling requires acetyl-CoA to activate the SASP. Building on our previous work demonstrating that cytosolic mtDNA activates the SASP via cGAS-STING signaling, we find that mtDNA signaling alone is not sufficient to drive the SASP. When citrate transport is inhibited, cytosolic mtDNA remains detectable, yet SASP gene expression is markedly reduced. Conversely, inhibition of mtDNA-cGAS-STING signaling suppresses the SASP without affecting histone acetylation, indicating that acetyl-CoA availability alone is insufficient in the absence of inflammatory input. Together, these findings show that mtDNA-dependent signaling requires a permissive chromatin state sustained by mitochondrial-derived acetyl-CoA. Thus, mitochondrial outputs are functionally hierarchical: mtDNA provides the inflammatory signal, whereas acetyl-CoA availability determines whether SASP genes can be transcriptionally activated.

4. This pathway can be targeted in vivo to modulate inflammaging. Inhibition of the citrate pathway *in vivo* reduces inflammatory gene expression while preserving the senescent state and without broadly altering systemic metabolism. These findings indicate that the inflammatory component of senescence can be selectively modulated. Consistent with this, analysis of human tissues shows that SLC25A1 expression is strongly correlated with mitochondrial-dependent SASP gene signatures across multiple organs, including muscle, heart, brain, and kidney (Extended Figure 16). Notably, this association is independent of chronological age and

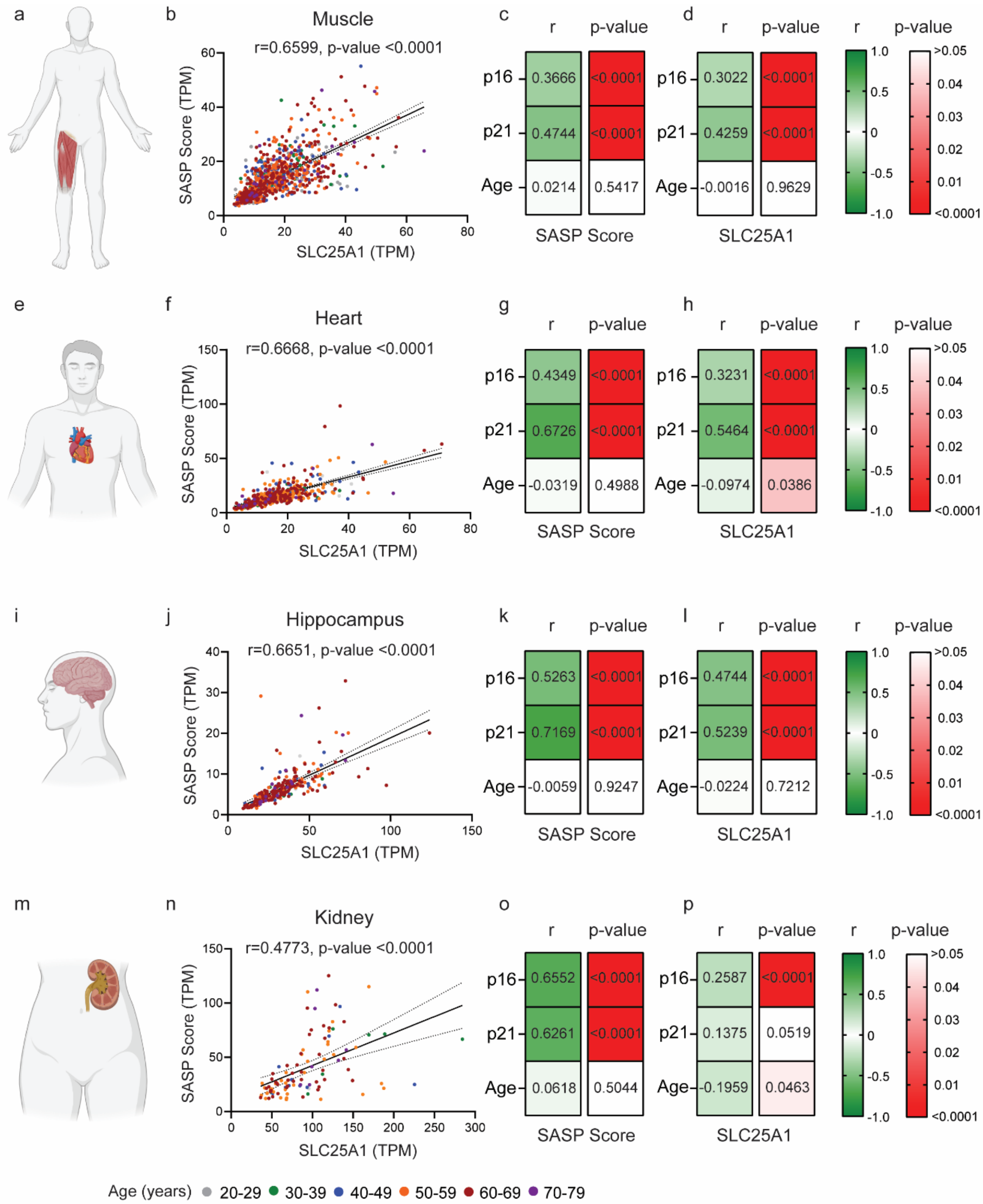

Extended Data Figure 16. SLC25A1 expression correlates with SASP and senescence markers in human tissues. Schematic illustrations of the analyzed organs: skeletal muscle **(a),** heart **(e),** hippocampus **(i),** and kidney **(m).** Correlation analysis between *SLC25A1* expression and mitochondrial dependent SASP score in each human tissue **(b, f, j, n).** Pearson correlation coefficients and associated p values between SASP score and p16, p21, and age in each tissue **(c, g, k, o).** Pearson correlation coefficients and associated p values between *SLC25A1* expression and *p16*, *p21*, and age in each tissue **(d, h, l, p).** Sample sizes: muscle (n=818), heart

correlates with senescence markers such as p16 and p21, supporting the relevance of this metabolic node for SASP regulation in humans.

Taken together, our study supports a framework in which the SASP is not a uniform output, but a selectively regulated program that depends on mitochondrial-derived acetyl-CoA to enable chromatin-based transcriptional responses. To our knowledge, the identification of a metabolic node that uncouples chromatin-driven inflammatory signaling from the senescent state *in vivo* represents a significant advance beyond prior studies focused on individual regulatory pathways.

Comments:

1. Many of the observed effects that are inferred as causal are likely correlation: the increase or decrease in H3K27 acetylation levels observed in SASP genes correlates with the expression/repression of SASP genes, and a similar relation might be observed if the SASP is inhibited by other means.

We agree that changes in histone acetylation can accompany transcriptional alterations without necessarily being causative. To address this, we provide several independent lines of evidence linking citrate-derived acetyl-CoA to chromatin regulation of the SASP.

First, we perturbed the acetyl-CoA pathway at two independent points: mitochondrial citrate export (SLC25A1) and its downstream conversion to acetyl-CoA (ACLY). In both cases, inhibition reduced SASP gene expression together with histone acetylation. Most likely, suppression of acetyl-CoA targets histone acetylation directly, not secondary to a decrease in gene expression.

Second, stable-isotope tracing demonstrated direct incorporation of glucose-derived carbon into histone acetylation in senescent cells. This labeling was markedly reduced by SLC25A1 inhibition (Extended Data Fig. 6f,g,h), providing biochemical evidence that mitochondrial citrate export supplies acetyl-CoA for histone acetylation in this context.

Third, epigenomic analyses revealed that H3K27ac-marked enhancer regions in senescent cells are strongly enriched near inflammatory and immune pathways, including core SASP genes. Since enhancers are known regulators of gene expression, this supports a role for histone acetylation driving SASP, and not vice versa. Consistent with a regulatory role at the chromatin level, *in vivo* single-nucleus multiome data showed selective reduction of chromatin accessibility at SASP loci following SLC25A1 inhibition (Fig.7a-k, Extended Data Fig.13, see in reviewer 1 response).

Importantly, we also examined conditions in which the SASP is reduced through an independent mechanism, namely disruption of the mtDNA/cGAS/STING pathway (BAX/BAK knockout or STING inhibition). In these settings, histone acetylation at SASP loci did not consistently track with changes in SASP expression (Fig. 5 and Extended Data Fig. 9, see response to reviewer 2), indicating that a decrease in SASP gene expression does not automatically result in a decrease in histone acetylation.

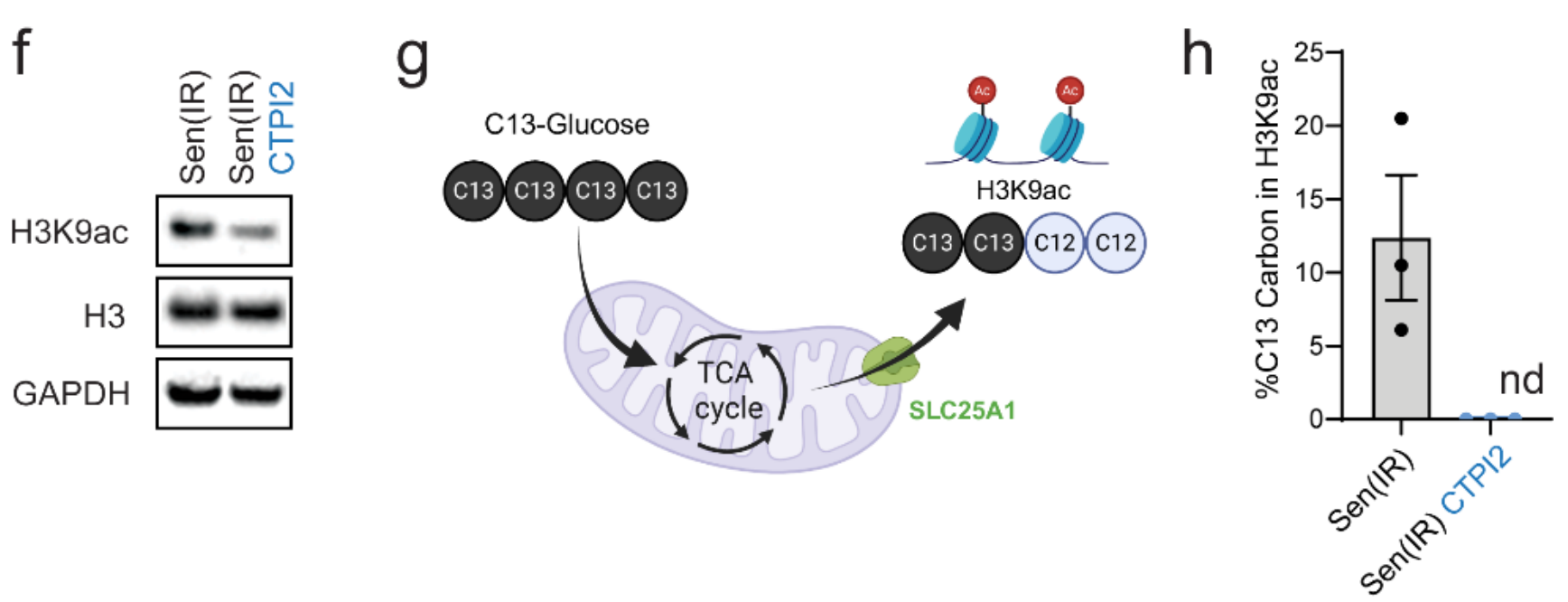

Extended Data Figure 6. (f) Western blot showing reduced histone acetylation (H3K9ac) following CTPI-2 treatment. **(g)** Schematic illustrating the ^{13}C-glucose tracing experimental approach. **(h)** Percentage of ^{13}C incorporation into H3K9ac after 24 hours in irradiation-induced senescent cells (Sen(IR)) treated with or without CTPI-2 (n=3 independent experiments). nd, not detected. Data are presented as mean ± s.e.m. Statistical analyses were performed using one-way ANOVA with Tukey's multiple comparison test.

2. The changes in H3K27 acetylation are suggested to be linked to changes in metabolites. No explanation is given on whether there is any specificity to H3K27 acetylation or whether similar effects are observed in other acetylation marks or protein acetylation.

We thank the reviewer for raising this point. Our data do not indicate specificity for H3K27ac alone. Mitochondrial-dependent SASP genes are enriched for multiple histone acetylation marks, including H3K18ac, H3K23ac, H3K122ac, H4K5ac, and H4K16ac, in addition to H3K27ac (Extended Data Fig. 2). Consistent with this, SLC25A1 inhibition reduced additional histone acetylation marks, including H3K9ac and H2BK20ac (Extended Data Fig. 6f and Extended Data Fig. 9c), and decreased nuclear acetylation at the protein level as assessed by

pan–acetyl-lysine immunofluorescence (Fig. 7l-p). H3K27ac was used as a representative mark of active regulatory chromatin.

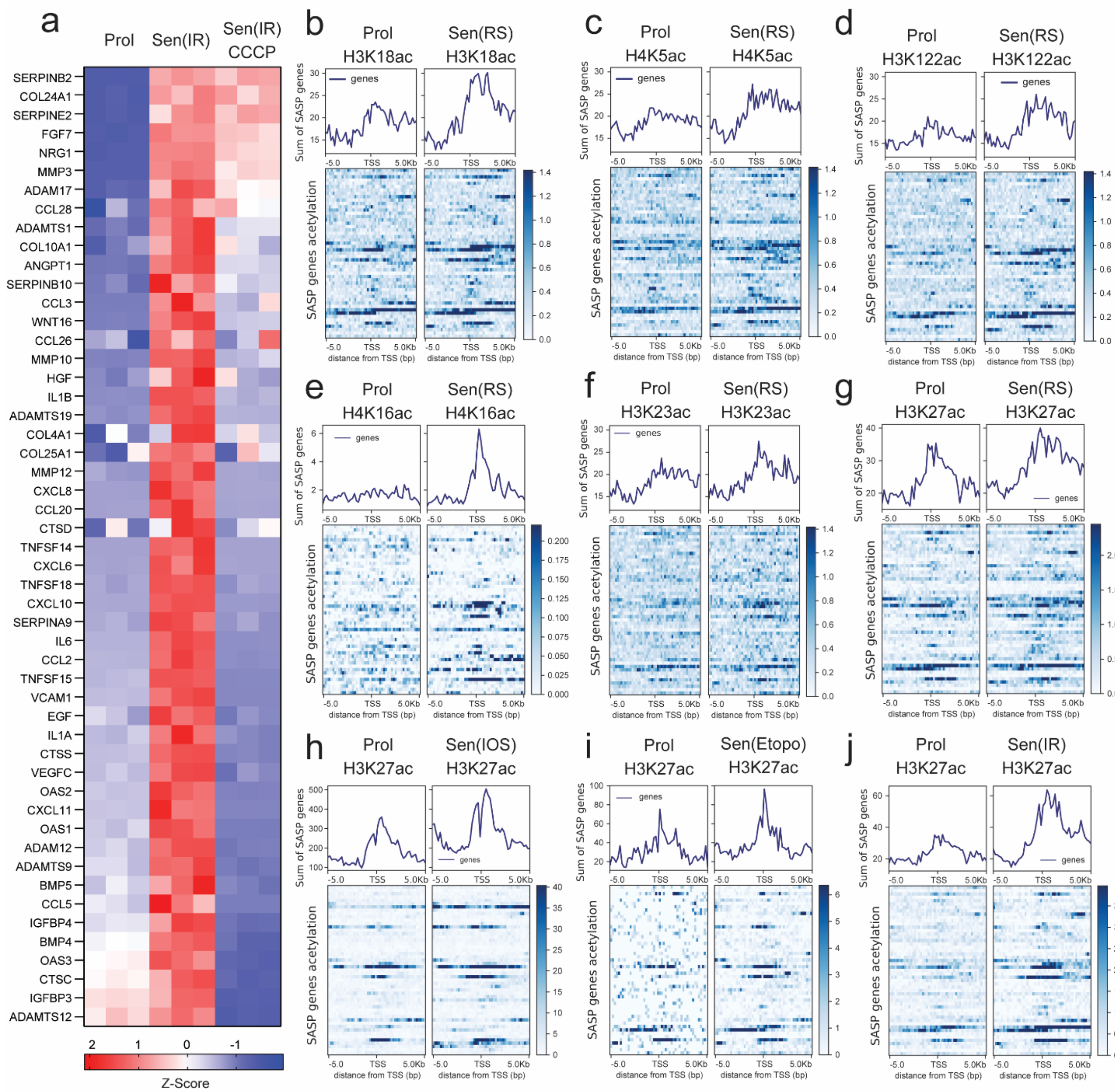

Extended Data Figure 2: Mitochondria-dependent histone acetylation patterns (H3K18ac, H3K5ac, H3K122ac, H4K16ac, H3K23ac, and H3K27ac) at SASP genes.

(a) Heatmap of 51 SASP genes differentially expressed in senescent IMR90 cells (Sen(IR)) and reduced following mitochondrial depletion via CCCP-induced mitophagy (Sen(IR)+CCCP). Color scale indicates column Z-score (red, high; blue, low expression). **(b-f)** Distribution of histone acetylation marks (H3K18ac, H3K5ac, H3K122ac, H4K16ac, and H3K23ac) at SASP genes in replicative senescent (RS) IMR90 fibroblasts. Top: Metaplot showing the composite signal across all 51 genes. Bottom: Heatmap of acetylation enrichment at individual genes (±5 kb from transcription start sites, TSS). Data reanalyzed from GSE106146 and GSE56307. **(g-j)** H3K27ac enrichment at SASP genes in IMR90 cells induced into senescence by distinct triggers. Top: Metaplot showing the composite signal across all 51 genes. Bottom: Heatmap of acetylation patterns at individual genes (±5 kb from TSS). Data reanalyzed from GSE74238. GSE103590. and GSE106146.

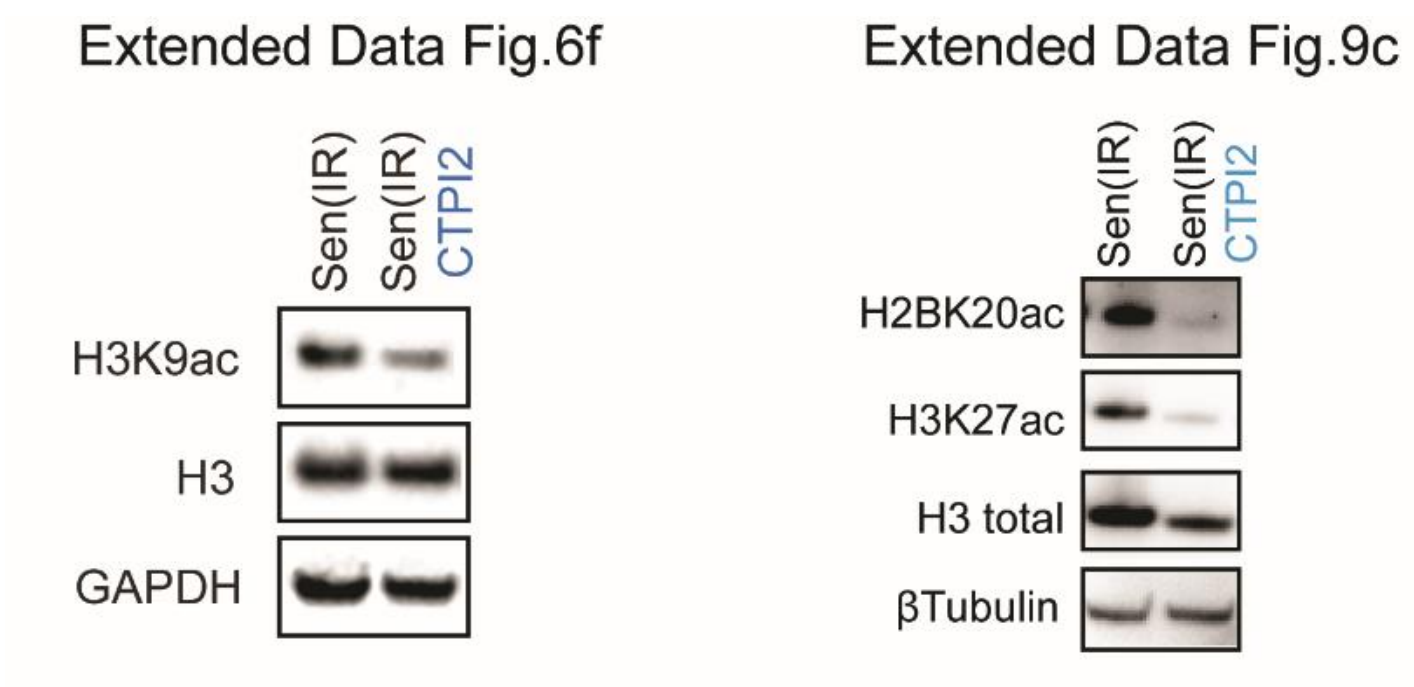

Extended Data Fig.6f: Western blot showing reduced histone acetylation (H3K9ac) following CTPI-2 treatment
Extended Data Fig.9c: Western blot showing reduced histone acetylation (H2B20ac, H3K27ac) after CTPI2 treatment in senescent (Sen(IR)) IMR90.

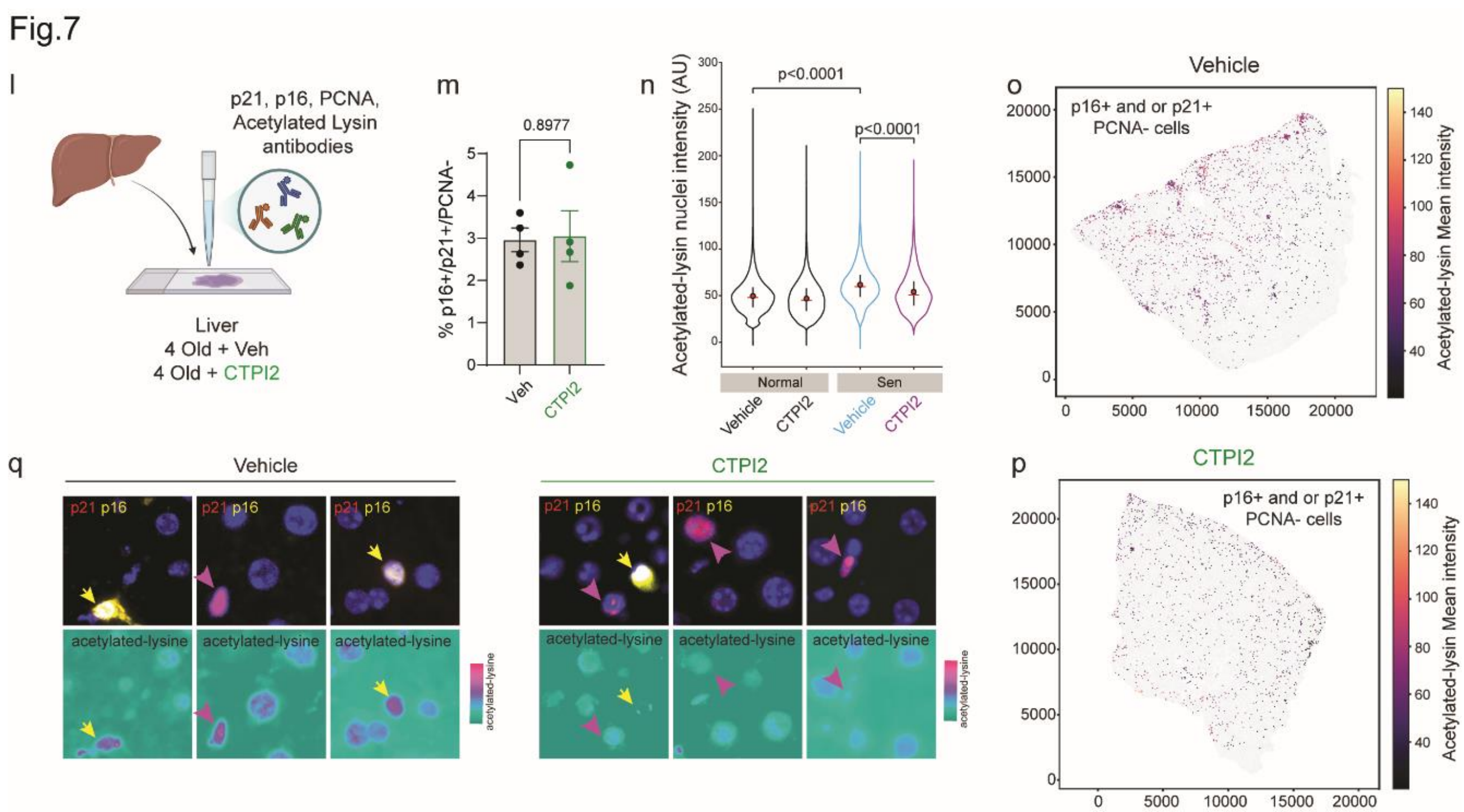

(l) Schematic of the experimental workflow for histological analyses. **(m)** Percentage of cells classified as p16$^+$ and/or p21$^+$ and PCNA$^-$ (non-proliferative) per sample. **(n)** Nuclear acetyl-lysine intensity in p16$^+$/p21$^+$/PCNA$^-$ cells (senescent) and normal cells in Vehicle- and CTPI2-treated groups. **(o-p)** Representative spatial plots of liver sections from Vehicle (o) and CTPI2-treated (p) mice showing the distribution of p16$^+$/p21$^+$/PCNA$^-$ cells and their nuclear acetyl-lysine intensity. **(q)** Representative immunofluorescence images of p21, p16, and acetyl-lysine staining in Vehicle and CTPI2-treated livers. Data in (d, g, i) are shown as mean values (red dots). Statistical analyses were performed using the Wilcoxon test (d, g, i), one-way ANOVA with Beferroni's multiple comparison test (n), or Student's *t*-test (m).

3. Activation of cGAS/STING is a key element to induce the SASP. No data is presented on whether the activation is decreased or not or why. The authors have previously described many alterations in mitochondria, including the release of mtDNA that affect the SASP, but how or whether those are affected has not been studied.

We thank the reviewer for this important comment regarding the relationship between cGAS/STING signaling and the acetate dependence of the SASP. Our previous work identified cytosolic mtDNA-mediated activation of

cGAS/STING as a key upstream driver of inflammatory signaling in senescent cells (PMID: 37821702). In the present study, we sought to determine how this pathway relates to mitochondrial regulation of histone acetylation.

To address whether inhibition of mitochondrial citrate export affects mtDNA/cGAS/STING signaling, we quantified cytosolic mtDNA following SLC25A1 inhibition (Fig. 5a-d). Cytosolic mtDNA levels were unchanged in senescent fibroblasts, indicating that this pathway remains intact under these conditions.

We next examined the reciprocal relationship. Genetic suppression of mtDNA release (BAX/BAK-deficient cells) or pharmacological inhibition of STING reduced SASP gene expression but did not alter global histone acetylation or H3K27ac levels, including at SASP loci (Fig. 5e-l; Extended Data Fig. 9e-k).

These observations prompted us to test whether the two pathways functionally interact. Acetate supplementation increased histone acetylation even when STING signaling was inhibited but did not restore SASP gene expression (Extended Data Fig. 9h-k). Conversely, mtDNA signaling did not alter histone acetylation levels.

Finally, in mitochondria-depleted senescent cells, combined supplementation of acetate and mtDNA more effectively restored SASP gene expression than either treatment alone (Fig. 5m-q; Extended Data Fig. 9l,m).

Together, these data indicate that mitochondrial citrate–dependent acetylation and mtDNA/cGAS-STING signaling represent distinct but complementary inputs into SASP regulation. mtDNA activates the cGAS/STING pathway, whereas SLC25A1-dependent acetyl-CoA availability acts as a key metabolic node that enables SASP gene expression.

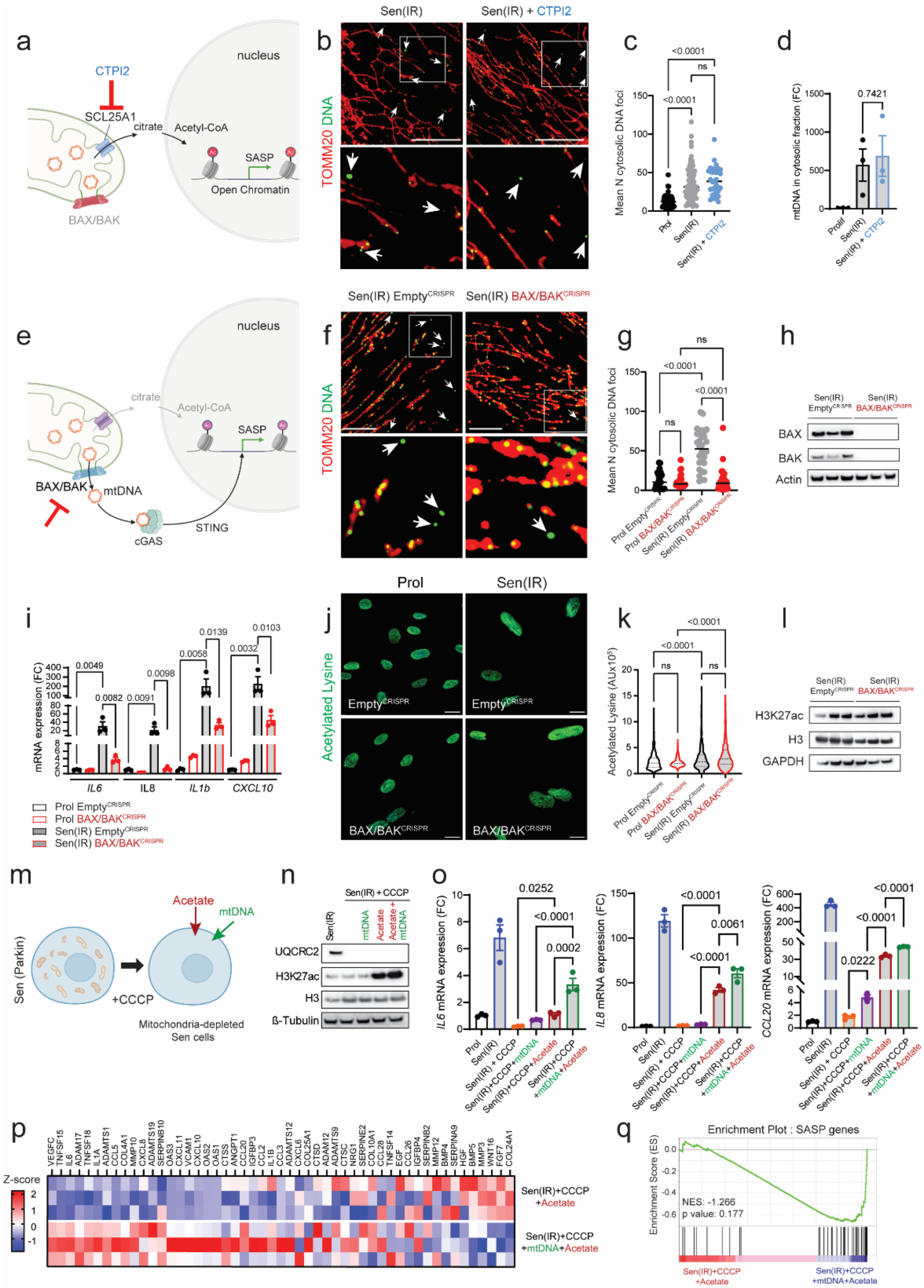

Figure 5: Mitochondrial citrate metabolism and mtDNA/cGAS/STING signaling independently regulate the SASP. (a) Schematic illustrating mitochondrial citrate export influencing histone acetylation at SASP genes. **(b)** Top panel: Representative immunofluorescence (IF) images of the mitochondrial network (TOMM20, red) and DNA staining (mtDNA, green) in senescent cells treated or not with CTPI-2 (MRC5 cells). White arrows indicate DNA foci outside the mitochondrial network. Scale bar = 20 µm. Bottom panel: Magnification of the white square. **(c)** Quantification of DNA foci outside the mitochondrial network per cell (n=3). **(d)** Cytosolic mtDNA levels expressed as fold change compared to proliferative cells (n=3). **(e)** Schematic illustrating mtDNA leakage and activation of the cGAS/STING pathway in senescent cells driving SASP expression. **(f)** Top panel: Representative IF of the mitochondrial network (TOMM20, red) and DNA staining (mtDNA, green) in senescent cells with CRISPR-Cas9 knockout of BAX and BAK and control (empty vector). White arrows indicate DNA foci outside the mitochondrial network. Scale bar = 10 µm. Bottom panel: Magnification of the white square. **(g)** Quantification of DNA foci outside the mitochondrial network per cell (n=3). **(h)** Western blot showing the efficiency of BAX and BAK knockout in senescent cells. **(i)** mRNA expression of SASP genes in proliferative (Prol) or irradiation-induced senescence (Sen(IR)) CRISPR-Cas9 BAX/BAK KO cells and controls. Data expressed as fold change relative to Prol (n=3). **(j)** Representative IF of nuclear acetylated lysine (green). Scale bar = 30 µm. **(k)** Quantification of nuclear acetylated lysine integrated density per cell (n=3). **(l)** Western blot showing no change in histone acetylation (H3K27ac) in Sen(IR) CRISPR-Cas9 BAX/BAK KO cells compared to controls. **(m)** Schematic representation of the experimental setup. **(n)** Western blot confirming the absence of mitochondria (UQCRC2) in mitochondria-depleted conditions (Sen(IR) + CCCP) and showing histone acetylation (H3K27ac). **(o)** mRNA expression of SASP genes under the indicated conditions. Data expressed as fold change relative to proliferative cells (Prol) (n=3). One-way ANOVA with Tukey's multiple comparison test was performed on the last four Sen(IR)+CCCP groups. **(p)** Heatmap of SASP factors in Sen(IR) cells lacking mitochondria supplemented with acetate alone or acetate plus mtDNA. Z-score representation (n=3). **(q)** GSEA enrichment plot of SASP factors showing higher expression in mitochondria-depleted senescent cells supplemented with acetate and mtDNA compared to acetate alone. Data are shown as Mean ± s.e.m. Statistical tests: One-way ANOVA with Tukey's multiple comparison test (c,g,k,o), uncorrected Fisher's LSD (i), or Student's t-test (d). (n= independent experiment)

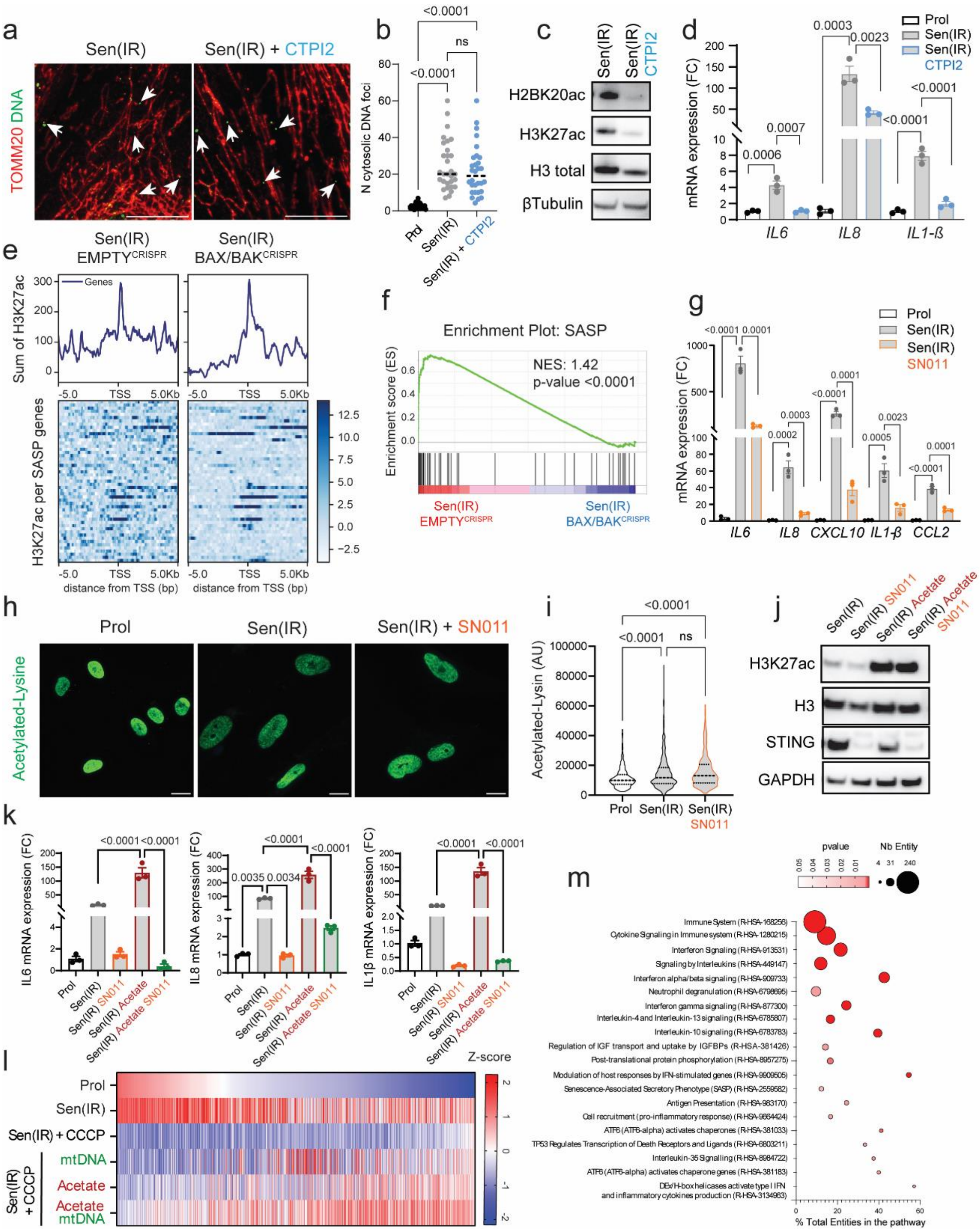

Extended Data Figure 9: Mitochondrial citrate metabolism and mtDNA/cGAS/STING signaling independently regulate the SASP. (a) Representative immunofluorescence images of the mitochondrial network (TOMM20, red) and DNA staining (mtDNA, green) in senescent cells treated with or without CTPI2 in IMR90 cells. White arrows show DNA foci outside the mitochondria network. Scale bar = 20µm **(b)** Quantification of number of DNA foci outside the mitochondria network per cell, from n=3. **(c)** Western blot showing reduced histone acetylation (H2B20ac, H3K27ac) after CTPI2 treatment in senescent (Sen(IR)) IMR90. **(d)** mRNA expression of SASP genes in proliferative (Prol) or irradiation-induced senescence (Sen(IR)) IMR90 treated or not by CTPI2. Data expressed as fold change relative to proliferative cells (Prol). (n=3). **(e)** Metaplot and heatmap of H3K27ac at SASP genes in senescent cells lacking BAX and BAK, and control. Top: Metaplot depicting a composite sum of all normalized H3K27ac enrichment at SASP gene signals; Bottom: heatmap of H3K27ac enrichment normalized by total H3 (±5kb from TSS). Data are averaged from n=2. **(f)** GSEA enrichment plot for the SASP factors between Sen(IR) Empty^{CRISPR} and BAX/BAK^{CRISPR}. **(g)** mRNA expression of SASP genes in Prol or Sen(IR) MCR5 treated or not by SN011. Data expressed as fold change relative to proliferative cells (Prol). (n=3). **(h)** Representative immunofluorescence images of nuclear acetylated lysine (green) in proliferatives (Prol) and senescent cells (Sen(IR)) treated or not with the STING inhibitor SN011. Scale bar = 20 µm. **(i)** Quantification of nuclear acetylated-lysine (integrated density) *per* cell (n=3). **(j)** Western blot showing that senescent IMR90 increased histone acetylation (H3K27ac) when treated by acetate with modification when treated with STING inhibitor SN011 while STING expression is reduced after SN011 treatment. **(k)** mRNA expression of SASP genes in proliferative (Prol) or irradiation-induced senescence (Sen(IR)) IMR90 treated or not by SN011 or acetate (n=3). **(l)** Heatmap of all genes showing an increase by acetate supplementation and mtDNA transfection compared to senescent cells lacking mitochondria. The color intensity represents the column Z-score. Data are represented with the average of the 3 z-scores per conditions (n=3). **(m)** Reactome pathways analysis of genes in (l). Data are shown as Mean ± s.e.m. Statistical tests: One-way ANOVA with Tukey's multiple comparison test. (n= independent experiment).

Referee #3 (Remarks to the Author):

The manuscript by Martini et al describes a series of experiments to link acetyl-CoA production to the expression of genes often associated with senescence-associated secretory phenotype (SASP). The approaches included removal of mitochondria, inhibition of the citrate transporter and pyruvate carrier, as well as gene knockouts (like ACLY, ATP-citrate lyase). Not surprisingly, when the authors manipulated the ability of the cells to produce acetyl-CoA there was a corresponding decrease in chromatin acetylation and gene expression. The authors focused on the expression of SASP genes, which as reported previously, are controlled by histone acetylation, along with most responsive genes. The authors note that inhibiting these metabolic pathways generating acetylCoA does not affect cell cycle arrest, which is classically associated with senescence and SASP. Moving to a mouse model, the authors show that SLC25A1 inhibition reduces inflammation and extends healthspan in aged mice. Overall, the study appears to be carefully performed and interpreted. Also, the mouse studies showing that inhibition of the citrate transporter improved inflammatory endpoints and other age-associated physiological changes is an important finding. However, two relatively significant weaknesses dampen enthusiasm. Histone acetylation and increased gene expression (of SASP and other genes) being dependent on production of acetyl-CoA is well established. The second is the lack mechanistic insight on why/how SASP genes would be preferentially targeted, if they are at all.

We thank the reviewer for the careful evaluation of our work and for highlighting both the importance of the *in vivo* findings. We agree that the dependence of histone acetylation and transcription on acetyl-CoA availability is well established. The focus of our study is on how a mitochondrial pathway supplying cytosolic acetyl-CoA influences the inflammatory program of senescent cells.

Across multiple independent perturbations, mitochondrial removal, inhibition of the citrate transporter (SLC25A1), inhibition of the mitochondrial pyruvate carrier, and genetic disruption of ACLY, SASP gene expression was consistently reduced, while p16/p21 expression and cell-cycle arrest remained intact. These observations separate the inflammatory output of senescence from the growth-arrest program.

In vivo, SLC25A1 inhibition in aged mice improved inflammatory and functional endpoints without major changes in body composition, energy expenditure, glucose homeostasis, or lipid metabolism (Extended Data Fig. 15, see response to reviewer 1).

To examine how these effects manifest at the chromatin level, we performed single-nucleus multiome profiling in aged muscle. Senescent-like nuclei showed increased chromatin accessibility and transcription at SASP genes, both of which were reduced following SLC25A1 inhibition, while broader transcriptional programs were largely preserved. In parallel, histone acetylation in vivo was reduced preferentially within senescent cells rather than uniformly across tissues (Fig. 7; Extended Data Fig. 13, see response to reviewer 1).

We then examined how this metabolic input relates to inflammatory signaling. In the revised manuscript, acetate supplementation increased histone acetylation but only partially restored SASP gene expression. This rescue remained limited in the absence of mtDNA/cGAS-STING signaling, whereas combined restoration of acetate-dependent acetylation and mtDNA signaling produced a stronger SASP response (Extended Data Fig. 9l,m, see response to reviewer 2).

Together, our data identify mitochondrial citrate flux as a metabolic node that influences the inflammatory arm of senescence during aging. Rather than a general effect of acetyl-CoA on transcription, the results indicate that SASP intensity can be modulated independently of senescence arrest, linking mitochondrial metabolism to regulation of age-associated inflammation *in vivo*.

Overall highlights

The authors careful use of a host of reagents and methods to demonstrate the role of acetyl-CoA in gene expression.

The use of acetate to partially rescue the phenotype is interesting and was not entirely expected, as ACSS2 would have to be expressed at sufficient amounts, perhaps induced after an acetyl-CoA deficit.

We thank the reviewer for this observation. We agree that acetate-mediated rescue requires sufficient ACSS2 activity to convert acetate into acetyl-CoA. To directly assess its metabolic use, we performed stable isotope tracing with ^{13}C-acetate. This analysis showed rapid incorporation into acetyl-CoA, with maximal labeling reached at approximately 3 hours after supplementation (Extended Data Fig.3b) and maintained for at least 24 hours. These data indicate efficient conversion of acetate into acetyl-CoA in this system.

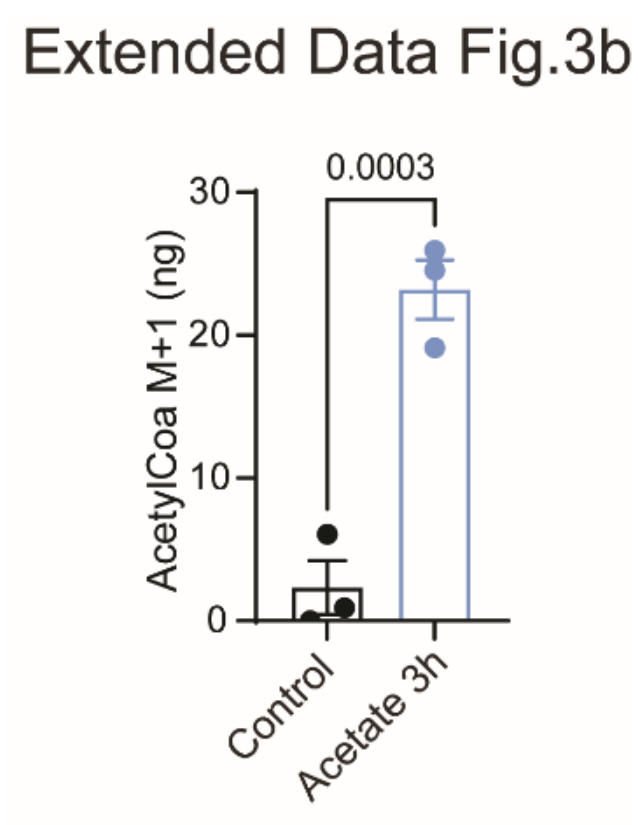

Extended Data Fig.3b: Incorporation of ^{13}C-labeled acetate into acetyl-CoA (M+1) measured 3 h after supplementation (n=3).

The in vivo studies are promising.

We thank the reviewer for this encouraging comment. We independently replicated the intervention in a separate cohort of aged mice and observed consistent improvements across functional and inflammatory measures, supporting the robustness and reproducibility of the phenotype.

Questions/Concerns

The fact that acetyl-CoA metabolism is connected to histone acetylation and gene expression is well-established. What makes this specific for SASP genes? If it truly is, then what is the mechanism? Do ACLY and ACSS2 sit at the enhancer/promoters of only SASP genes and directs p300 K27 acetylation? Why only those loci?

We thank the reviewer for raising this important question of specificity. To test whether ACLY might directly regulate SASP loci, we examined its subcellular localization in senescent cells. Biochemical fractionation showed that ACLY remains predominantly cytoplasmic in both proliferating and senescent cells, with no detectable enrichment in the nuclear fraction. These data do not support a model in which ACLY is selectively recruited to specific promoters or enhancers, although we cannot exclude that other factors may contribute to locus-specific recruitment of acetylation machinery (Figure 1a).

We therefore examined why reduced acetyl-CoA availability would preferentially affect SASP genes. Analysis of our ChIP-seq datasets showed that H3K27ac-marked enhancer regions (distal regulatory elements that control gene transcription) in senescent cells are strongly enriched near inflammatory and immune pathways, including core SASP components (Figure 1b). This suggests that senescence establishes an enhancer landscape that is biased toward inflammatory gene regulation. Consistent with this, our in vivo single-nucleus multiome data show that chromatin accessibility at SASP loci is reduced in senescent-like cells following SLC25A1 inhibition, while the senescent state itself is preserved.

Together, these observations suggest that the apparent specificity does not arise from selective targeting of acetyl-CoA producing enzymes to SASP loci, but from the increased dependence of these loci on an H3K27ac-enriched enhancer network. Further dissection of locus-specific recruitment mechanisms will require additional study and is beyond the scope of the present work. For this reason, and given that this question warrants a more detailed investigation, we have included these data for the reviewers' consideration but have not incorporated them into the manuscript.

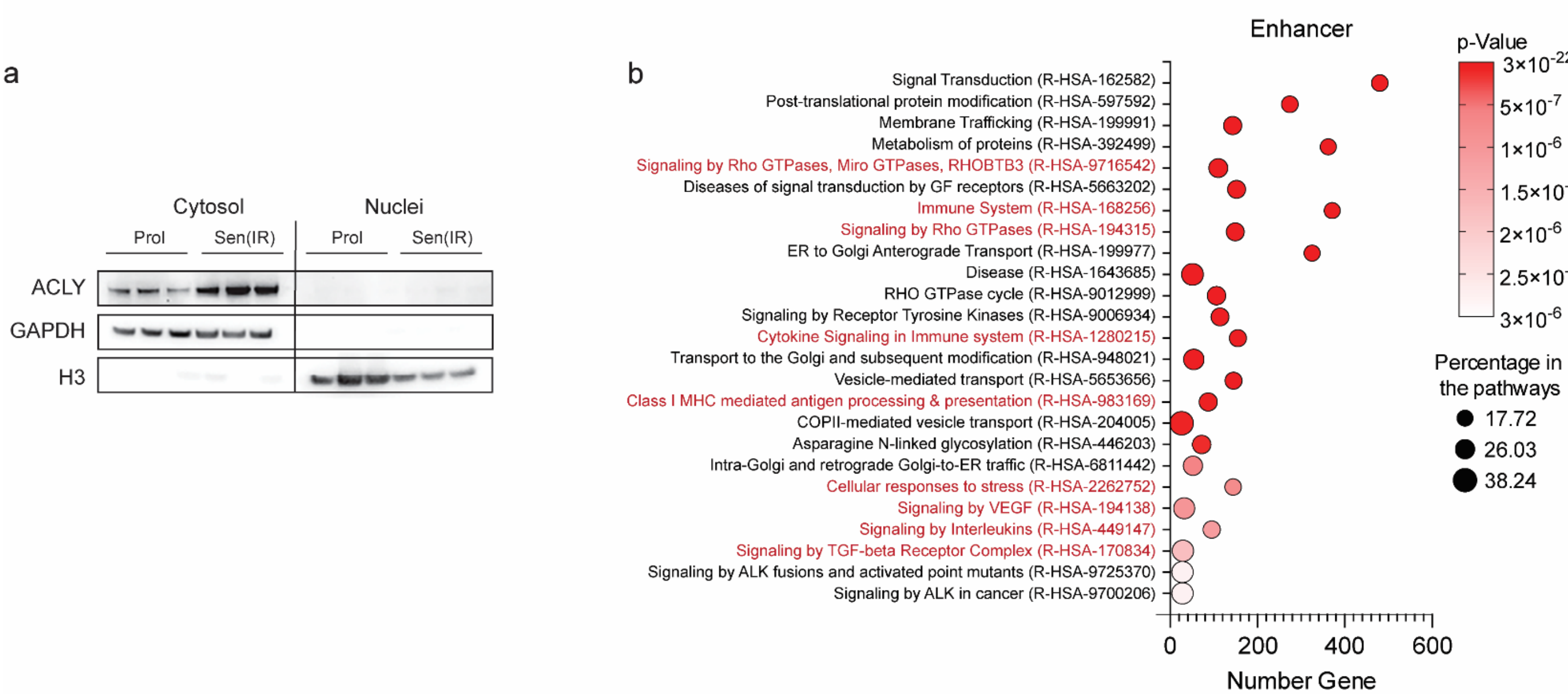

Figure 1 a. Immunoblot analysis of ACLY localization in proliferating (Prol) and senescent (Sen(IR)) cells. Cytosolic and nuclear fractions are shown. **b**. Pathway enrichment analysis of genes associated with senescence-linked enhancer regions. Dot plot showing significantly enriched Reactome pathways.

If other the classic markers of senescence are not changing, then is this really SASP. The authors narrowly focus on SASP genes, but what is the direct evidence that this is not just a global metabolic stress response?

We thank the reviewer for this important question. We consider cell senescence to be minimally comprised of two key phenotypes: proliferation arrest and SASP. When we state in our models that SASP is affected, we mean that expression of SASP genes is affected without affecting proliferation arrest (repression of cell cycle genes and expression of CDK inhibitors, e.g. p21 and p16).

To determine whether the observed effects reflect a general metabolic stress response or modulation of the SASP within senescent cells, we analyzed our *in vivo* single-nucleus multiome data.

This analysis allowed us to distinguish senescent-like cells from non-senescent populations in aged tissues. Senescent-like nuclei exhibited increased expression of SASP genes together with increased chromatin accessibility at SASP loci compared to non-senescent cells. Following SLC25A1 inhibition, these same cells retained canonical features of senescence, including cell cycle arrest, but showed reduced SASP gene expression and decreased chromatin accessibility at SASP loci. These changes therefore occur within senescent cells themselves rather than reflecting a global transcriptional response across cell types (Fig.7 and Extended Data Fig.13, see response reviewer 1).

We also examined this at the protein level in an independent tissue. In liver, RNA-sequencing showed broad suppression of SASP factors following SLC25A1 inhibition, while the proportion of $p16^+$ and $p21^+$ cells at the protein level remained unchanged. At the same time, nuclear pan-acetyl-lysine expression was elevated in these cells and reduced following SLC25A1 inhibition (Fig.7).

To assess whether these effects reflect a global metabolic stress response, we performed comprehensive metabolic phenotyping across tissues in aged mice after SLC25A1 inhibition. Glucose tolerance, body weight, body composition, energy expenditure, respiratory exchange ratio, and activity were largely unchanged, and lipid and fatty acid metabolic pathways were not significantly altered (Extended data Fig.15, see response reviewer 1).

What is the direct evidence of actual secretion of factors in cell culture media and mouse serum?

Following the reviewer's suggestion, we performed a plasma cytokine analysis in aged mice treated with or without CTPI2. We observed that CTPI2 treatment reduces the plasma levels of several cytokines, as shown in Extended Data Fig.12a. In senescent cells in vitro, we observed that inhibition of MPC or SLC25A1 significantly reduced several secreted SASP proteins (see Extended Data Fig. 4f and Extended Data Fig. 6a)

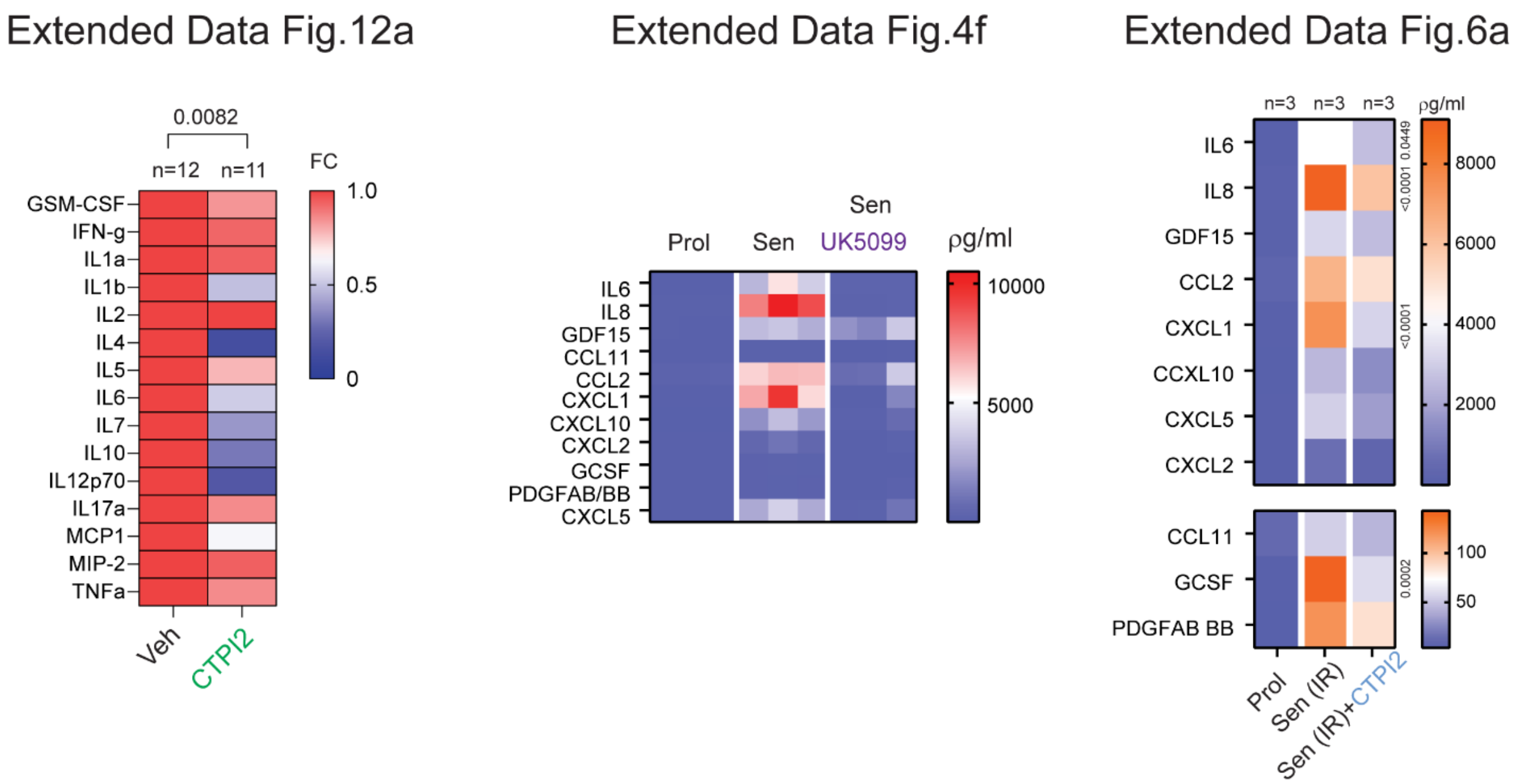

Extended Data Fig.12a: Heatmap of plasma cytokine levels from vehicle- or CTPI2–treated mice. Data are expressed as fold change compared to vehicle-treated animals (n=12 vehicle, n=11 CTPI2, male and female combined).
Extended Data Fig.4f: Cytokine array heatmap of 24-hour conditioned media from senescent cells, showing cytokine expression changes.
Extended Data Fig.6a: Heatmap of cytokine array data from 24-hour conditioned media, presented as the average of n=3 independent experiments *per* condition.
