# Supplementary material for: Mitochondrial metabolism and epigenetic crosstalk drive SASP
Source: Nature. 2026 Jul 29;656(8129):980–92. doi: 10.1038/s41586-026-10791-2 (PMC13518223; doi:10.1038/s41586-026-10791-2)

Figure1b

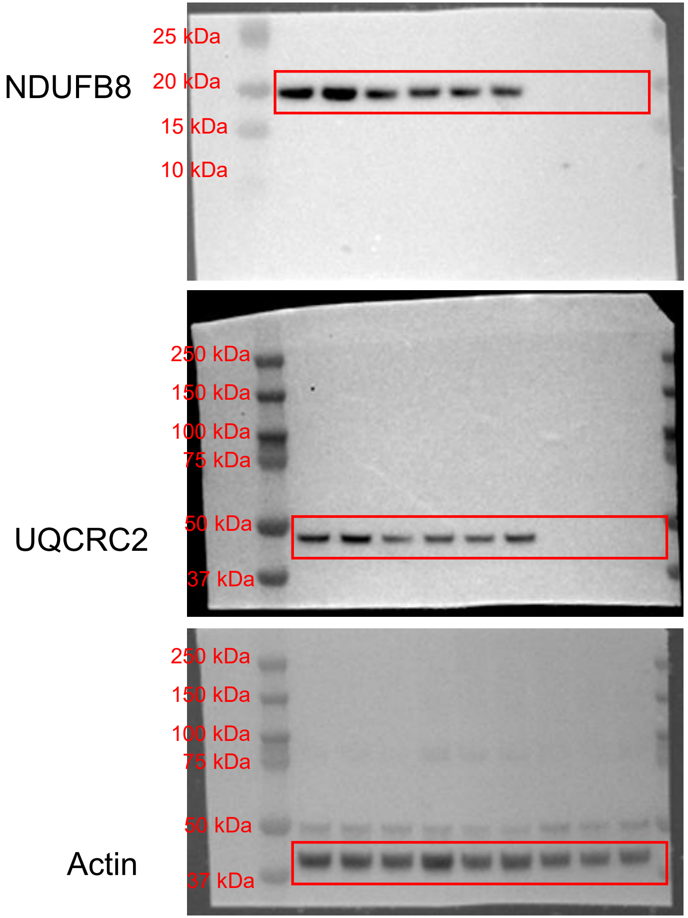
NDUFB8
25 kDa
20 kDa
15 kDa
10 kDa
UQCRC2
250 kDa
150 kDa
100 kDa
75 kDa
50 kDa
37 kDa
Actin
250 kDa
150 kDa
100 kDa
75 kDa
50 kDa
37 kDa

Extended Figure 3e

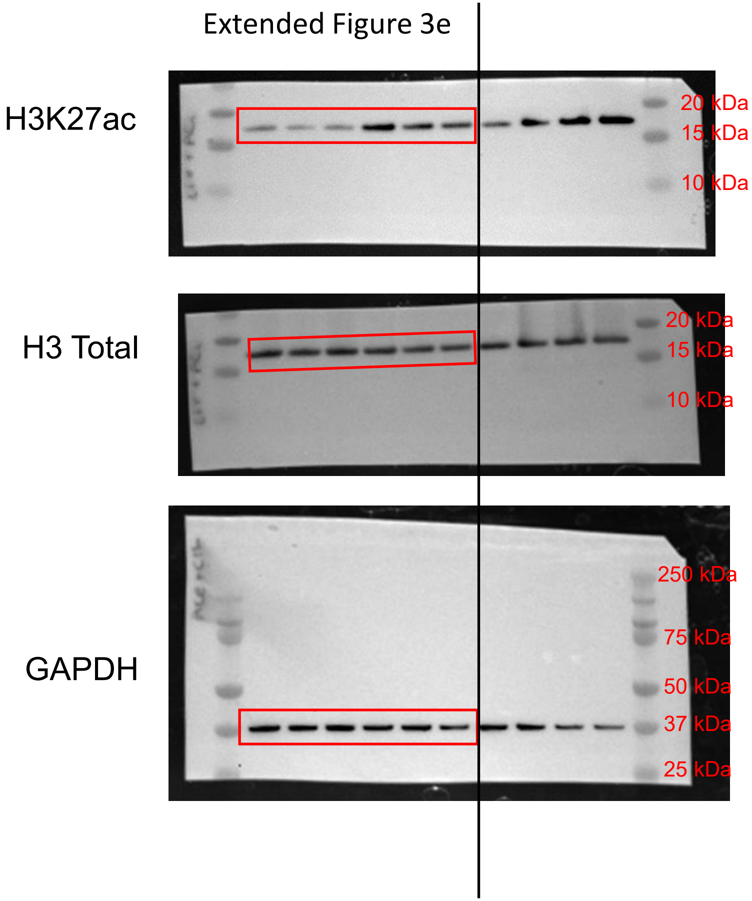

Extended Figure 4b
Sen(IR)

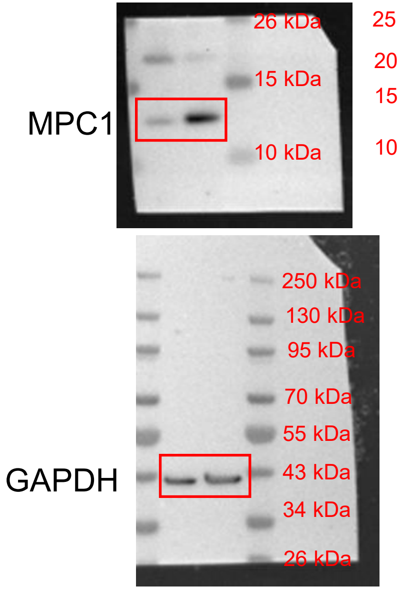

Extended Figure 4b
Sen(Dox)

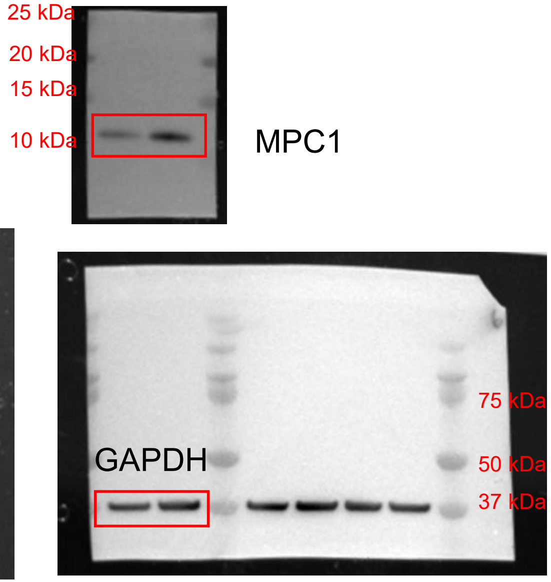

Extended Figure 4b
Sen(RS)

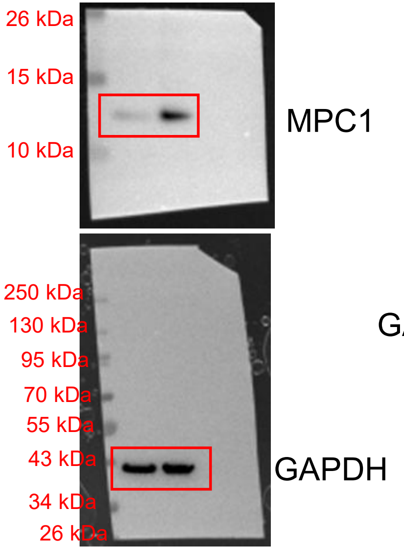

Extended Figure 4h

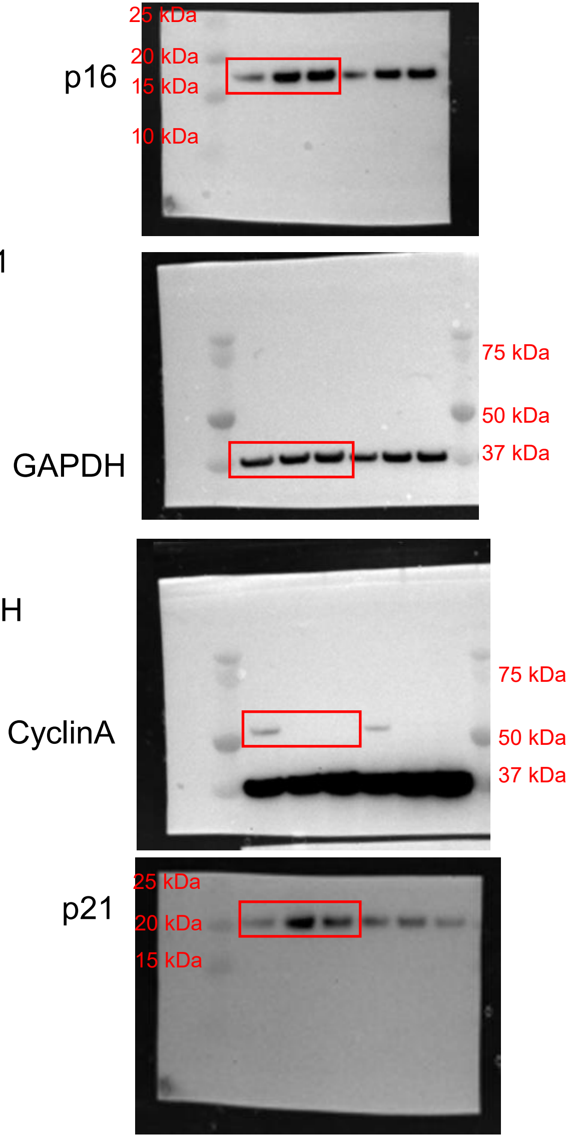

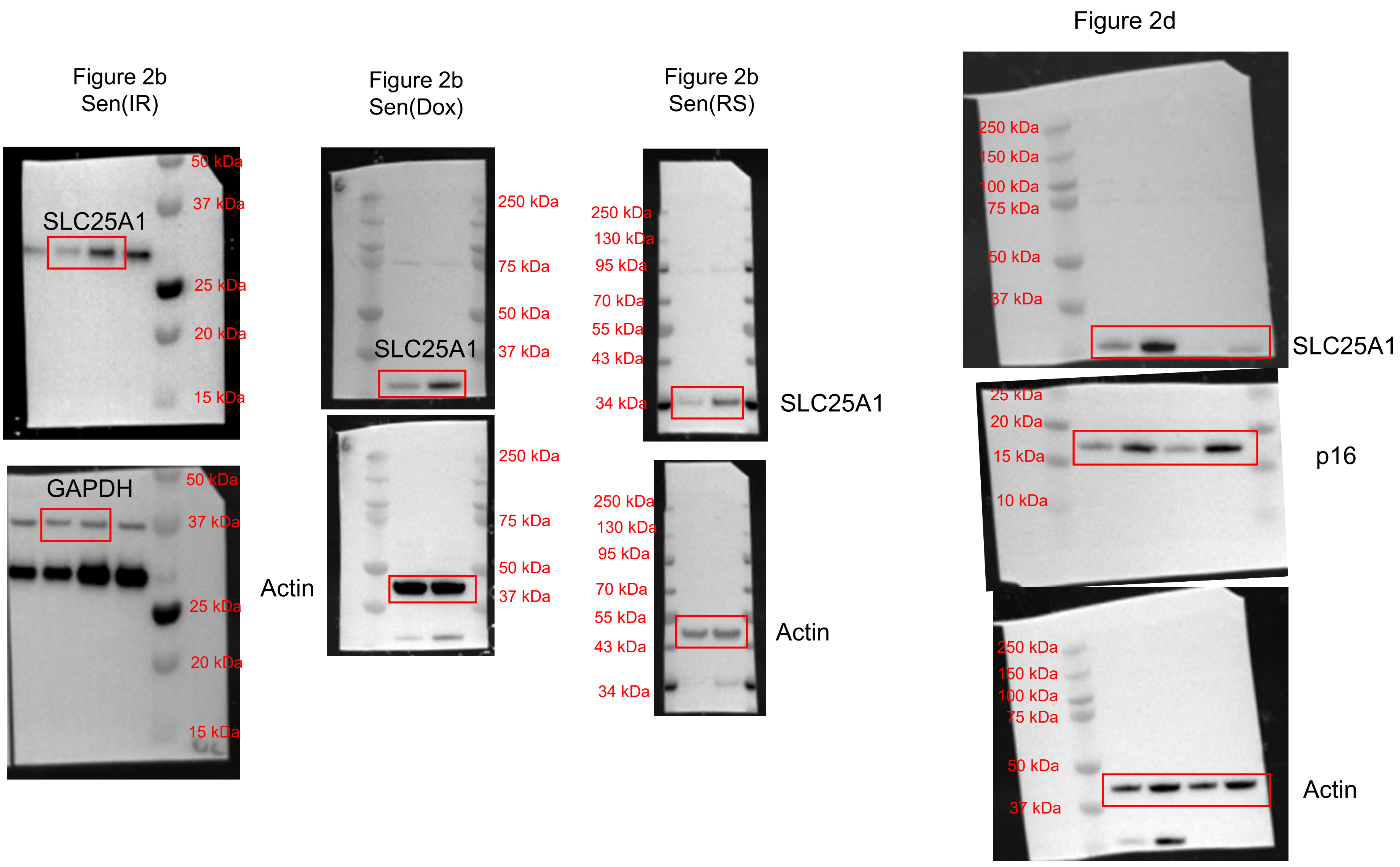
Figure 2b
Sen(IR)
Figure 2b
Sen(Dox)
Figure 2b
Sen(RS)
Figure 2d
SLC25A1
50 kDa
37 kDa
25 kDa
20 kDa
15 kDa
GAPDH
50 kDa
37 kDa
25 kDa
20 kDa
15 kDa
250 kDa
75 kDa
50 kDa
37 kDa
SLC25A1
Actin
250 kDa
75 kDa
50 kDa
37 kDa
250 kDa
130 kDa
95 kDa
70 kDa
55 kDa
43 kDa
34 kDa
SLC25A1
250 kDa
130 kDa
95 kDa
70 kDa
55 kDa
43 kDa
34 kDa
Actin
250 kDa
150 kDa
100 kDa
75 kDa
50 kDa
37 kDa
SLC25A1
25 kDa
20 kDa
15 kDa
10 kDa
p16
250 kDa
150 kDa
100 kDa
75 kDa
50 kDa
37 kDa
Actin

Figure 3h

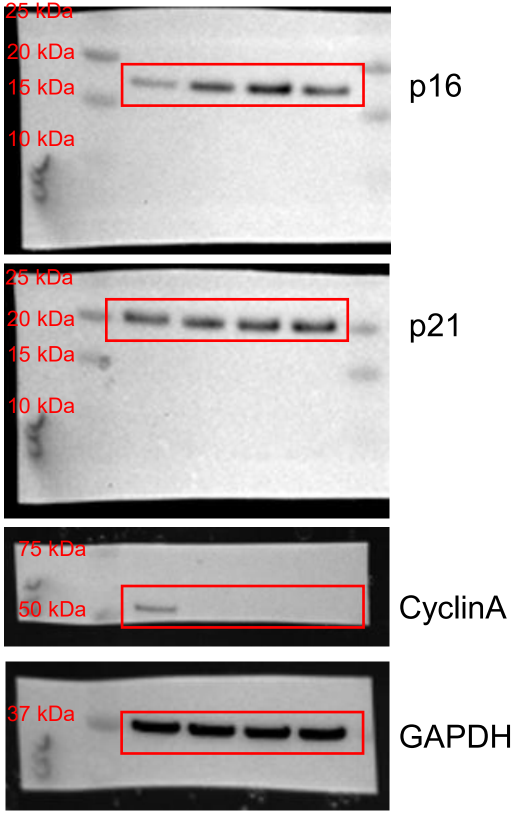

25 kDa
20 kDa
15 kDa
10 kDa
p16
25 kDa
20 kDa
15 kDa
10 kDa
p21
75 kDa
50 kDa
CyclinA
37 kDa
GAPDH

Extended Figure 5k

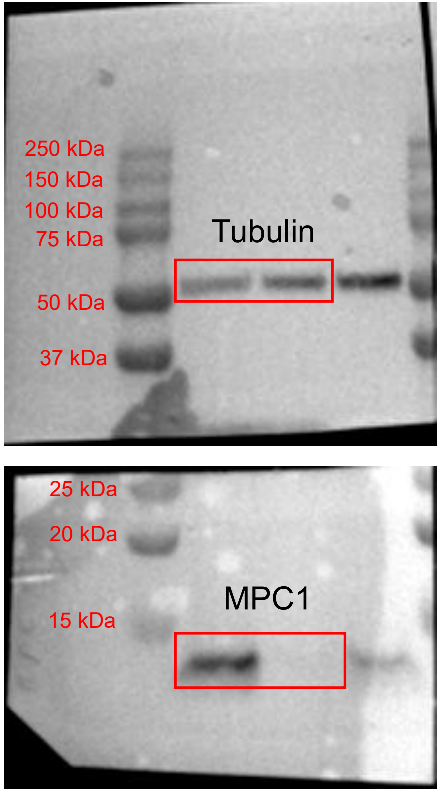

Extended Figure 6f

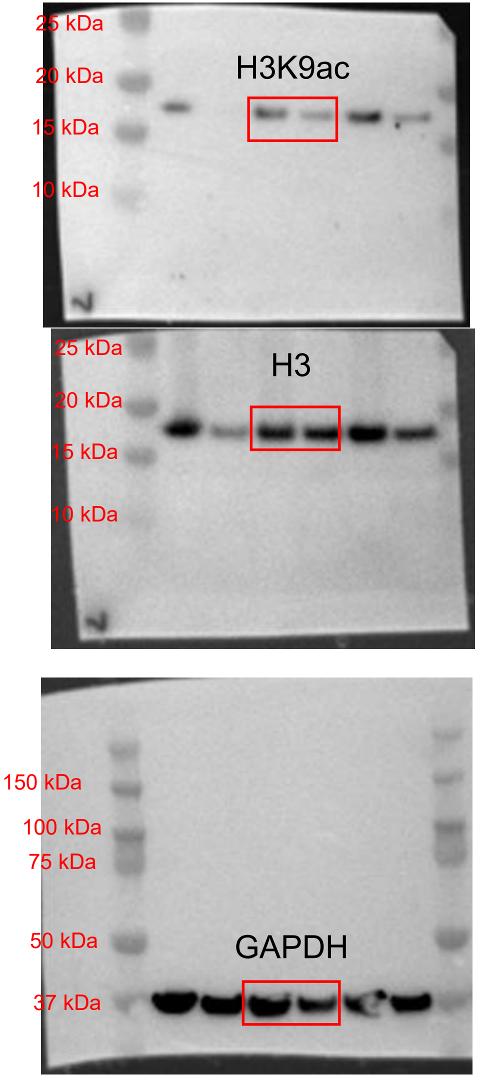

Figure 4c

Figure 4f
Sen(IR)

Figure 4h
Sen(RS)

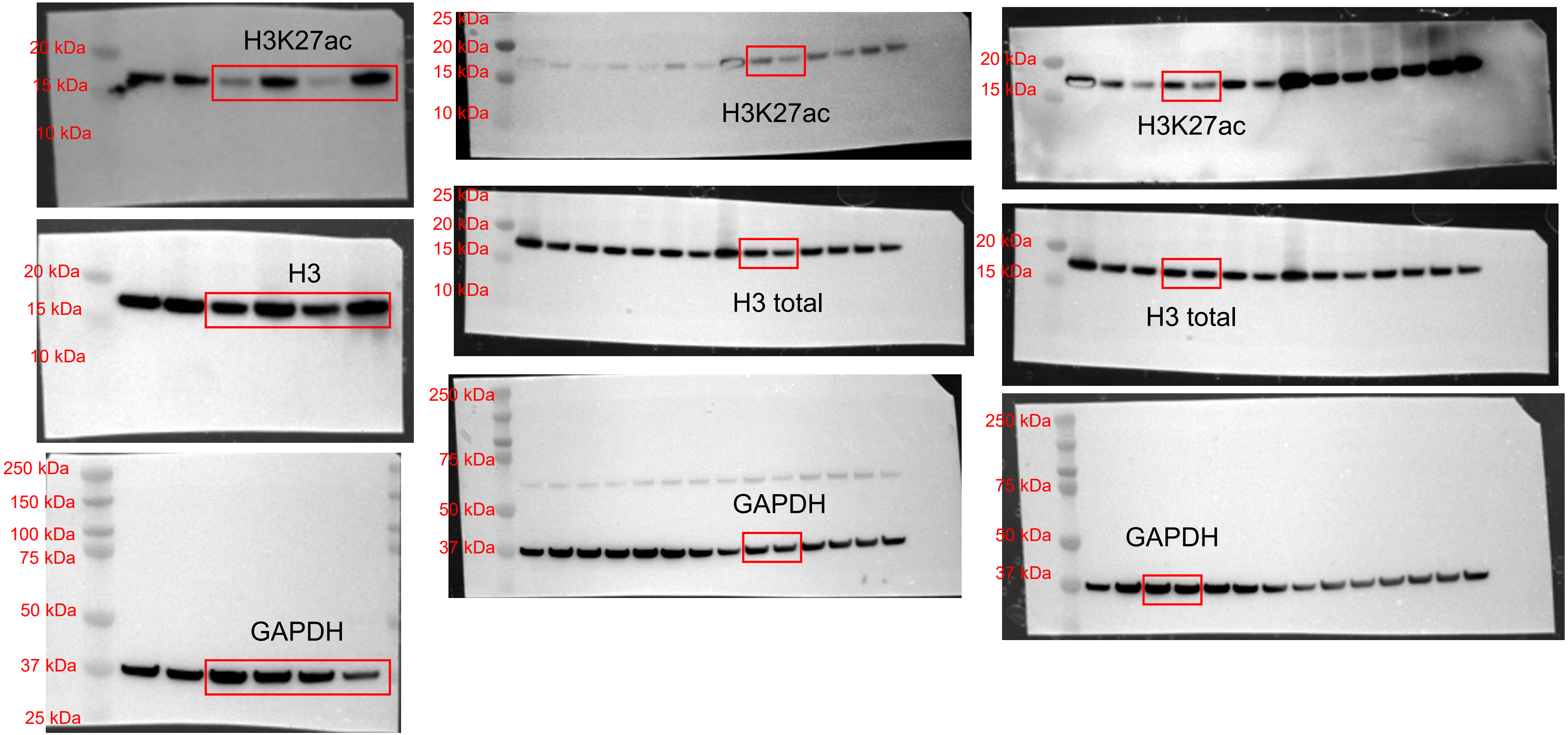

Figure 5h

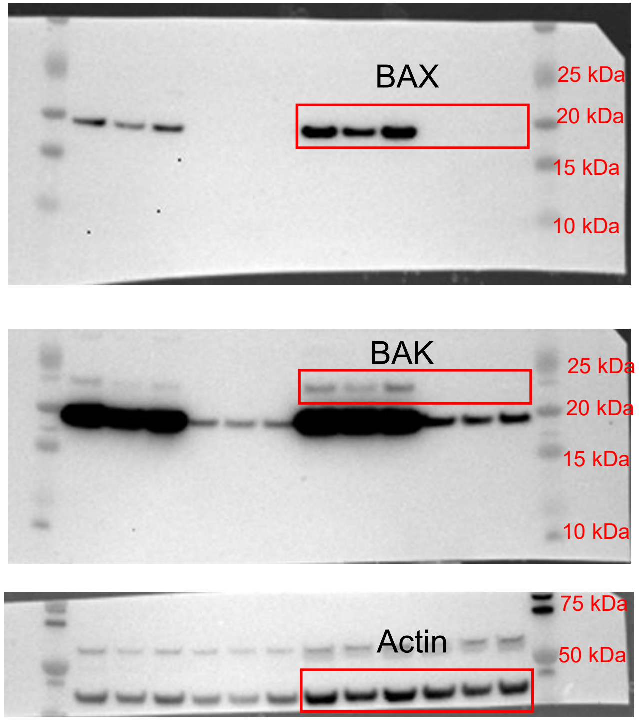

Figure 5l

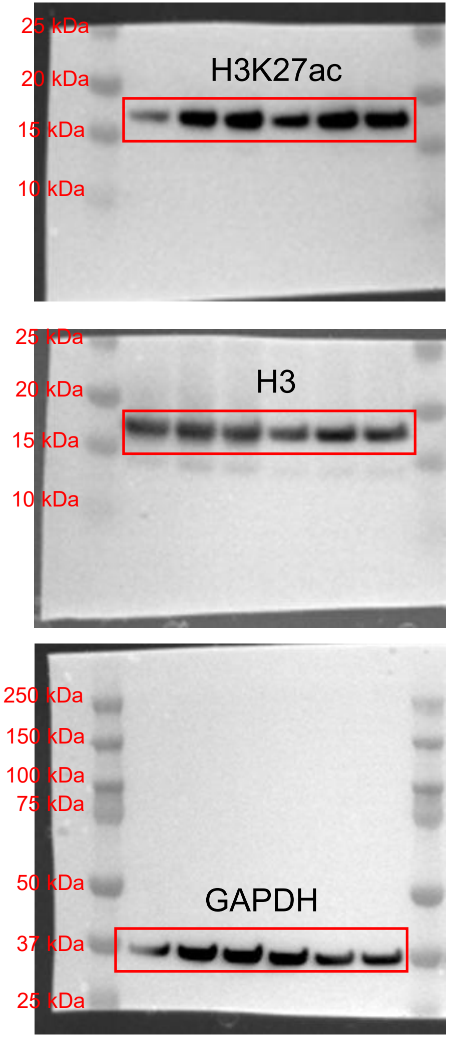

Figure 5n

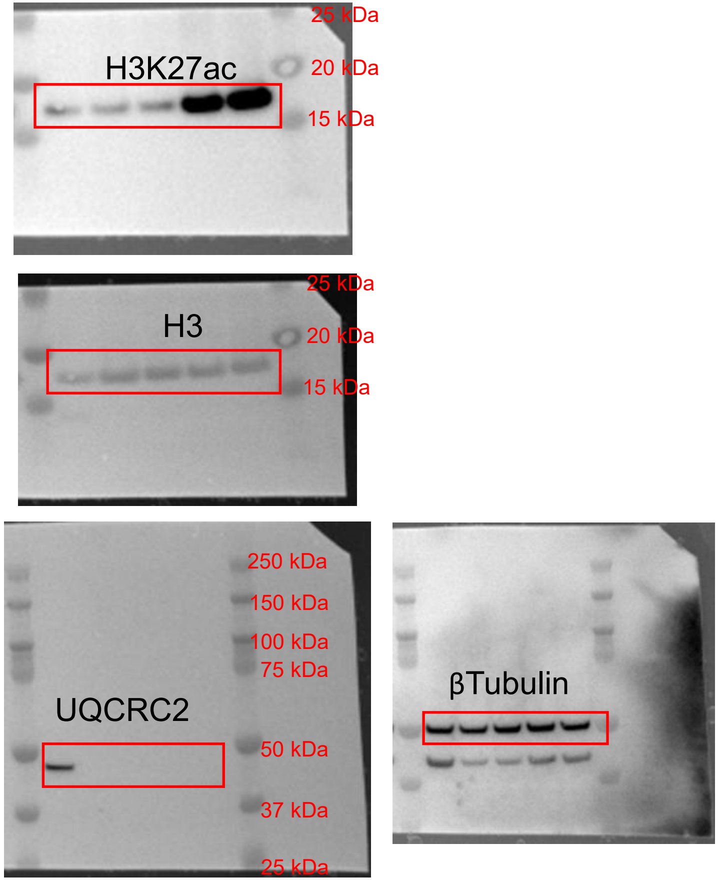

Extended Figure 9c

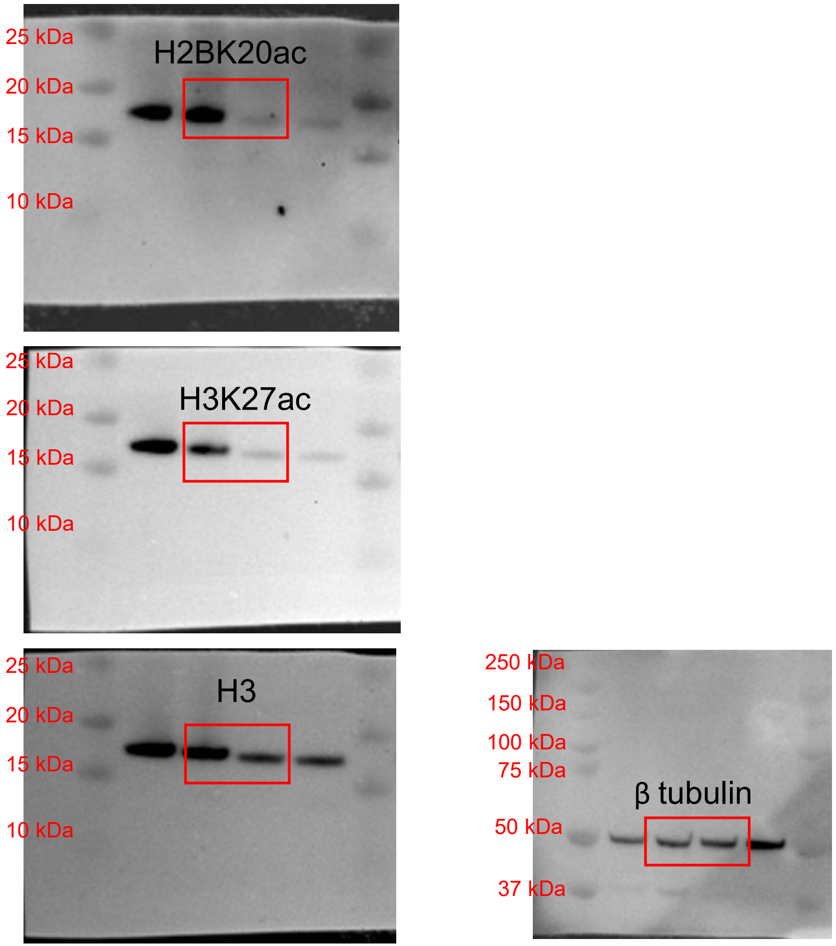

Extended Figure 9j

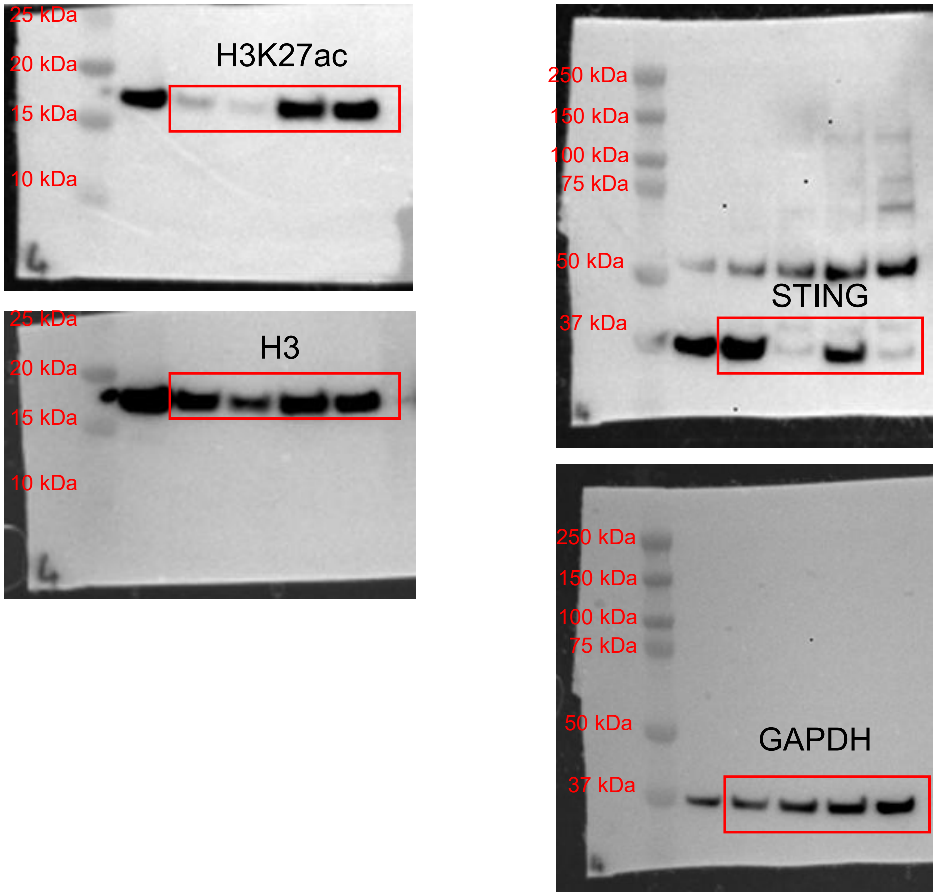

Supplement: Supplementary file 4 [file 41586_2026_10791_MOESM4_ESM.zip › 2024-10-22132B-f1-7;sf1-13/Source Data Western Blots.pdf]
